# Are Reproducible Dietary Patterns Consistently Associated With Disease Outcomes or Their Drivers in Italy? A Systematic Review

**DOI:** 10.1016/j.advnut.2025.100397

**Published:** 2025-02-27

**Authors:** Rachele Bianco, Monica Ferraroni, Michela C Speciani, Maria Parpinel, Valeria Edefonti

**Affiliations:** 1 Department of Medicine—DMED, Università degli Studi di Udine, Udine, Italy; 2 Branch of Medical Statistics, Biometry and Epidemiology ‘G. A. Maccacaro,’ Department of Clinical Sciences and Community Health Dipartimento di Eccellenza 2023-2027, Università degli Studi di Milano, Milano, Italy; 3 Fondazione IRCCS Ca’ Granda Ospedale Maggiore Policlinico, Milano, Italy

**Keywords:** a posteriori dietary patterns, consistent associations between reproducible dietary patterns and disease outcomes, correlates of dietary patterns, cross-study reproducibility of dietary patterns, drivers of dietary patterns, disease outcomes, factor analysis, Italy, principal component analysis, systematic review

## Abstract

The strength, direction, and trend of associations between specific diseases and reproducible a posteriori dietary patterns (DPs) based on principal component analysis (PCA) or exploratory factor analysis (EFA) have rarely been investigated across populations. We conducted a systematic review of PCA/EFA-based DPs identified in Italy to explore 2 methodological issues: *1*) cross-study reproducibility of Italian DPs; *2*) consistency of associations between reproducible DPs and the same/similar disease outcomes/DP drivers/correlates. The systematic review process and findings on DP cross-study reproducibility were published separately. This paper focuses on associations, summarizing the data in figures and tables, with post-hoc criteria for similarity among target variables, statistical methods, and adjustment for confounding. Predefined rules of inference were used to evaluate selected Hill’s causal criteria (consistency, strength, and dose–response effects) and draw valid scientific conclusions on the association between PCA/EFA-based DPs and similar/the same target variables. Fifty-two articles, primarily on EFA-based DPs derived from food frequency questionnaires, were included. Regression models were used to explore the relationships between DPs and disease outcomes/DP drivers, aligning with original research questions, study designs, and literature on confounding. When considering similar target variables, 9 groups of reproducible DPs showed >50% statistically significant associations in the same direction across 1–3 groups of target variables, such as socioeconomic characteristics, incidence of chronic diseases, overall/cause-specific mortality, cardiovascular disease risk factors, pregnancy/breastfeeding-related and elderly-related outcomes. Groups targeting dairies/sweets and vegetable sources of fats showed >50% nonsignificant findings across all similar target variables. Overall, 54% of findings were nonsignificant. When considering the same target variable, the median number of DPs per group was equal to 2 (interquartile range: 2–2.5). Together with population comparability issues, this prevented us from reliably performing any meta-analyses. At this stage, valid scientific conclusions cannot be drawn to inform Italian nutritional recommendations.

This study was registered at PROSPERO as registration number CRD42022341037.

**Statement of Significance**

On the basis of this methodological project from Italy, collected evidence from a carefully designed systematic review was evaluated for reproducibility of a posteriori dietary patterns from PCA/EFA and associations with disease outcomes, DP drivers/correlates. Statistical and nutritional knowledge was used for creating groups of reproducible DPs. A selection of Hill’s criteria with predefined rules of inference (majority-rules criterion and feasibility of meta-analysis for consistency of associations, selected cut-offs for strength of associations, and evaluation of trends for dose–response effects) was evaluated jointly with limitations of included study designs, and statistical methods used (including control for confounding) to reach valid scientific conclusions on DPs from Italy. Causal conclusions, if reached, may contribute to inform the next releases of Italian dietary guidelines.

## Introduction

Dietary patterns (DPs) play a key role in exploring the relationship between diet and health or disease outcomes, capturing the complexity of eating behaviors within populations. Unlike single-nutrient analyses, DPs account for multicollinearity issues and provide stronger associations with disease risks [[1], [2], [3], [4], [5], [6], [7]]. Over the past decade, DPs have become foundational in updating the Dietary Guidelines for Americans, despite the persistence of significant gaps in research [8]. In addition to the extensive efforts of the USDA’s Nutrition Evidence Systematic Review Branch [8], smaller scale methodological projects can address these gaps and offer insights for developing national dietary guidelines.

Focused on promoting reproducible research [9], our group has contributed to clarifying terminology around the reproducibility, validity, and reliability of a posteriori DPs [10,11]—those derived from multivariate statistical methods such as principal component analysis (PCA), exploratory factor analysis (EFA), and cluster analysis [2,12,13]. Recently, we conducted a systematic review focused on a posteriori DPs from PCA/EFA in Italy, our country of origin, to investigate 2 key issues:

1) cross-study reproducibility of Italian DPs (that is, the reproducibility of DPs across populations in Italy), and
2) consistency of associations between reproducible DPs and similar/the same health/disease outcomes in Italy.

We published the first set of results in a companion article [14] presenting the search strategy for the systematic review and evaluating DP cross-study reproducibility [11], along with its sources, including populations, dietary assessment tools, input variables (that is, food groups or nutrients), and methods for identifying DPs. This paper aims to determine whether the identified DPs, organized by reproducibility, are consistently associated with health/disease outcomes, drivers, or correlates of interest across available Italian studies. We also investigated if potential artifacts were minimized by consistent approaches to the statistical methods used, including adjustment for confounding.

Specific projects have shown consistent associations between adherence to a priori DPs—those aligned with benchmark diets [2]—and disease incidence and mortality, employing comparable study designs and food frequency questionnaires (FFQs), as well as standardized definitions of indexes and protocols for statistical analyses [[15], [16], [17], [18], [19], [20], [21], [22], [23]]. Higher index scores for nearly all investigated DPs were associated with lower disease risks or mortality across the articles [[15], [16], [17], [18], [19], [20], [21], [22], [23]]. However, the consistency of associations between reproducible a posteriori DPs and specific health/disease outcomes remains less explored. For instance, one Spanish case-control study examined the link between (original and “reconstructed”) PCA-based DPs and breast cancer risk, revealing a high consistency in DP compositions and relations with risk. Hence, it suggested a framework for applying a posteriori DPs across different populations while studying sources of reproducibility and consistency of the associations [24].

After our investigations into the sources of PCA/EFA-based DP reproducibility in Italy [14], we further explored sources of the consistency of their associations with health/disease outcomes, and the results obtained for Italian researchers or public health professionals. Although evaluating consistency in nutritional epidemiology is challenging [25], collecting evidence across various populations, study designs, and statistical methods must be seen as the first step in assessing the consistency of the associations. Along with other scientific and ethical considerations, evaluating the collected evidence from PCA/EFA-based DPs identified in Italy through predefined causal criteria can provide valid scientific conclusions [25]. These conclusions may, in turn, inform the next releases of nutritional recommendations in Italy.

Our research, therefore, addressed the following questions:

1) Which statistical methods were employed to evaluate the relationship between PCA/EFA-based DPs and disease outcomes, DP drivers, or correlates of interest in Italy?
2) Which confounding variables were accounted for in the relationship between PCA/EFA-based DPs and disease outcomes, DP drivers, or correlates of interest in Italy?
3) Were there available data on similar/the same target variables, including disease outcomes, DP drivers, or correlates of interest related to PCA/EFA-based DPs in Italy?
4) As far as similar/the same target variables were available for reproducible DPs, and consistent statistical approaches were used (including control for confounding), were the relationships between these reproducible DPs and similar/the same disease outcomes, drivers, or correlates of interest consistent?

## Methods

This paper presents a second set of results from a systematic review on PCA/EFA-based DPs in Italy, reported in accordance with the PRISMA 2020 guidelines [26]. Comprehensive details regarding the systematic review process, study quality evaluation [27], study characteristics, DP identification methods, and the cross-study reproducibility of DPs in Italy were provided in the companion article [14]. A brief summary of previous findings is reported in the Methods and Results sections of this paper. Supplemental Figure 1 illustrates the integration of evidence at both article and aggregate levels, along with the corresponding research questions addressed across the 2 companion articles within this project. The pathway links reproducible DPs (as discussed in [14]) to their consistent associations with the same/similar health/disease outcomes/DP drivers/correlates of interest (as explored in this paper). The gray boxes highlight results that have already been stated in the companion article [14].

The review protocol was registered with the PROPERO database and was subsequently updated after the publication of the first article [14] (registration no: CRD42022341037). The review process was efficiently managed using the EndNote 20 software (Thomson Reuters).

### Systematic review process: a brief outline

The electronic literature search was conducted by inserting strings based on keywords and controlled vocabulary terms around the fields of DPs, factor analysis, PCA, and Italy in Medline/PubMed, Embase, and Cochrane CENTRAL and Reviews on 21 December 2022 (Supplemental Methods, text).

Articles were considered eligible for inclusion if: *1*) they were original full-text articles published in peer-reviewed journals; *2*) the study enrolled human subjects residing in Italy; *3*) they identified DPs based on PCA and/or EFA using dietary data, regardless of any further analysis on health or disease outcomes, DP drivers, or correlates. Exclusion criteria are provided in Supplemental Methods, text.

In addition to previously reported data, we extracted information on: *1*) available health/disease outcomes, drivers, or correlates of interest; *2*) statistical methods employed to relate the identified DPs to disease outcomes/DP drivers/correlates, and *3*) key results regarding the relationship between identified DPs and disease outcomes/DP drivers/correlates (corresponding to statistical models adjusted for all available confounders, when applicable).

### Qualitative and quantitative assessment of cross-study reproducibility of identified DPs: a brief outline

In the qualitative assessment of cross-study reproducibility of all available and most recent PCA/EFA-based DPs in Italy [14], similarity plots based on original text descriptions and factor loadings illustrated groups of reproducible DPs in adults described as follows:

1) same row: original text descriptions were materially identical and relevant loadings were very similar;
2) different rows with the same color code: original text descriptions and relevant loadings were similar;
3) different rows with variants of the same color code: original text descriptions or relevant loadings exhibited modest but nutritionally relevant differences within the same group.

In the quantitative assessment of cross-study reproducibility [14], we utilized the congruence coefficient (CC) (–1≤CC≤1), which is the preferred index for measuring the similarity of PCA/EFA-based DPs [28,29]. This assessment was conducted across 18 articles that had adopted the same lists of input variables (that is, either nutrients or food groups) (Supplemental Figure 1).

### Single DPs and disease outcomes, DP drivers, or correlates: aggregate-level synthesis of findings

Collected evidence on the association between single DPs and related target variables was summarized by examining:

1) *target variables*: we categorized target variables (that is, variables potentially related to the identified DPs) based on the original research question as:
  i) *correlates*: when descriptive statistics (for example, correlation coefficients, Cohen's kappa coefficients, or Bland–Altman plots), possibly integrated with hypothesis testing, was proposed;
  ii) *drivers*: when fitted regression models included the driver as the independent variable and each principal component/factor score as the dependent variable. For our purposes, drivers are associated with—or related to—the identified DPs, but not in a causal relationship. A cause-and-effect relationship would require suitable study designs and fulfillment of other criteria outlined by Hill (1965) [30];
  iii) *health/disease outcomes*: when fitted regression models included principal component/factor scores as independent variables and the health/disease outcome as the dependent variable;
In each category, we grouped similar variables on a post-hoc basis. We clarified which variables were similar in a dedicated table and displayed them in adjacent columns in the graphical synthesis of findings;

2) statistical methods used to relate identified DPs and disease outcomes/DP drivers/correlates of interest: we investigated if the statistical analyses reflected the research question and study design introduced in the original article;
3) adjustment for confounding variables in the relationship between DPs and disease outcomes/DP drivers/correlates of interest: we compared the lists of confounding factors accounted for in the groups of disease-oriented, driver-oriented, and correlate-oriented articles. We also compared the lists adopted in groups of similar disease outcomes/DP drivers/correlates. We finally investigated if key confounders selected in the disease-oriented articles reflected the major drivers or correlates investigated in the driver-oriented or correlate-oriented articles;
4) relationships between single DPs and disease outcomes/DP drivers/correlates of interest: while accounting for the original study designs, we evaluated the number and percentages of statistically significant relationships involving single combinations of DPs and:
  i) any disease outcomes or DP drivers/correlates, regardless of the specific variables examined;
  ii) similar disease outcomes or drivers/correlates investigated.

### Reproducible DPs and disease outcomes, DP drivers, or correlates: causal criteria and rules of inference

This paper broadens the previous narrative synthesis to determine whether reproducible DPs were consistently related to the same or similar disease outcomes/DP drivers/correlates of interest. This objective required criteria to be defined for:

1) DP reproducibility: reproducible DPs were indicated with the same group label across rows in the qualitative assessment of DP reproducibility [14]. In this second synthesis, within each group of reproducible DPs, we further condensed in the same row the nutritionally similar DPs that were originally represented with variants of the same color code [14]. This step was informed by evidence from the quantitative assessment of DP reproducibility [14];
2) similarity/equivalence of disease outcomes/DP drivers/correlates of interest: we focused our analysis on either similar target variables, as defined post-hoc in the first part of the analysis, or the same target variables, when available;
3) consistency of the statistical methods used to relate identified DPs and disease outcomes/DP drivers/correlates of interest: to avoid additional model-related artifacts, we investigated if the statistical analyses followed from the original research question and study design;
4) consistency of adjustment for confounding variables in the relationship between DPs and disease outcomes/DP drivers/correlates of interest: we compared the lists of confounding factors accounted for when the same target variable was investigated;
5) consistency of the relationships between reproducible DPs and the same/similar disease outcomes/DP drivers/correlates of interest: following a majority-rules criterion [25], we focused on combinations of groups of reproducible DPs and similar/the same target variables which showed either >50% of nonsignificant findings across all target variables or >50% of significant findings going in the same direction. We defined that direction across articles was the same when:
  i) positive or inverse (linear) relations were observed between the DP and the correlate of interest (correlate-oriented research questions);
  ii) increasing or decreasing trends in the driver means were observed across increasing quantile-based categories of principal component/factor score (driver-oriented research questions);
  iii) higher or lower risks of disease/death/adverse health outcome were associated with increasing DP scores (disease-oriented research questions).

Reported information for each combination included: number of associations/correlations involving each group of reproducible DPs, as well as number and percentage of statistically significant positive findings, statistically significant negative findings, and nonsignificant findings per similar target variable.

When the target variable was the same, we further applied an alternative rule of evidence and evaluated the consistency of associations based on the results of meta-analyses [25]. For these analyses, we collected the following information: group label for reproducible DPs, DP label, first author’s name for reference, study design, population, potential overlap of populations across studies, confounding factors, effect estimates, and the associated confidence intervals for upper categories of DPs or drivers. The feasibility of conducting single meta-analyses was assessed on a case-by-case basis, considering population comparability and overlap, the number of available effect estimates, and the measures used in each comparison, in line with the Cochrane Handbook and relevant references [31,32]. In cases where meta-analyses were not feasible, we provided a narrative synthesis describing the heterogeneity of study designs and adjustments for confounders. Finally, we evaluated the criteria of “strength of association” and “dose–response.” For the “strength of association,” a statistically significant risk estimate that is a >20% increase or decrease in risk was considered a positive finding, with a 40%–50% change considered strong. For the “dose–response,” we assessed the presence of a statistically significant linear or otherwise regularly increasing trend, to further support the evidence for causality [25].

## Results

### Article selection process and study quality: a brief outline

Of 193 eligible full-texts, 52 articles (all in English) [[33], [34], [35], [36], [37], [38], [39], [40], [41], [42], [43], [44], [45], [46], [47], [48], [49], [50], [51], [52], [53], [54], [55], [56], [57], [58], [59], [60], [61], [62], [63], [64], [65], [66], [67], [68], [69], [70], [71], [72], [73], [74], [75], [76], [77], [78], [79], [80], [81], [82], [83], [84]] remained after applying exclusion criteria (Supplemental Figure 2—PRISMA flowchart and [14] for details). Of these, 42 were based on "very good" or “good” quality studies (Supplemental Figure 3 and [14] for details).

### Study characteristics: a brief outline

The selected articles, published between 2001 and 2022 (with 79% released from 2010 onwards), covered 14 of Italy's 20 regions, and 9 research groups were responsible for ∼83% of the publications. The most common study design was the prospective cohort design (19 articles), followed by the cross-sectional design (17 articles), and by the case-control design (13 articles). Notably, 8 articles presented cross-sectional analyses of cohort studies; subsequently, cross-sectional analysis was applied in 25 of the articles included. The general population of adult males and females was included in 24 articles. Three articles focused exclusively on men, whereas 15 articles considered women, 6 of which specifically examined pregnant/breastfeeding women. Additionally, 10 other articles considered apparently healthy children or adolescents, community-dwelling elderly, and the entire household (0–75 y).

Dietary habits were typically assessed using a reproducible and valid FFQ administered at recruitment, with a reference period of 1 or 2 y. The median number of FFQ items was 95 (range: 31–217) (Supplemental Figure 4, details in Supplemental Table 1 and [14]).

### DP identification: statistical methods (brief outline)

Input variables for PCA/EFA were food groups in 33 articles and nutrients in 18 articles, with 1 article using both types of variables. Most analyses involved preprocessing input data, primarily through standardization. Among the included articles, 10 performed PCA, 41 performed EFA, and 1 [63] utilized both methods; EFA was typically applied following the PCA method. The number of components/factors to be retained was mostly determined using a combination of eigenvalue >1 or 2, Scree plot construction, or component/factor interpretability. A varimax rotation was applied in 45 articles. Most articles specified cut-offs for component/factor labeling (Supplemental Figure 4, details in Supplemental Table 2 and [14]).

### DP description and cross-study reproducibility: a brief outline

A total of 186 DPs were identified across all articles included in the systematic review. In the companion article [14], these DPs were collapsed into 113 distinct DPs, providing a 39.3% reduction of overall dietary information (Supplemental Figure 5). When additionally merging nutritionally similar DPs (that is, those with the same color code in Supplemental Figure 5), the 113 DPs from [14] were consolidated into 76 DPs, resulting in an additional 33% reduction (Supplemental Figure 6).

The identified DPs were further organized into 11 distinct groups of reproducible DPs (food-based groups: 6; nutrient-based groups: 5) featuring the following food group combinations (Supplemental Results, text, for details):

1) pasta and meat (2 groups, food-based ***Pasta-and-Meat-oriented*** and nutrient-based ***Starchy*** ***P******atterns***);
2) healthy-protein foods and a side dish (one group, the food-based ***Healthy-Protein Foods and Side Dish*** group);
3) fruit and vegetables (2 groups, the food-based ***Mixed-Salad*** and nutrient-based ***Vegetable-based Patterns***);
4) cheese and deli meats (2 groups, the food-based ***Dairy Products and Sweets*** and nutrient-based ***Animal-based Patterns***);
5) processed and ready-to-eat foods (1 group, the food-based ***Unhealthy Foods and Snacks*** group);
6) animal sources of fats (1 group, the nutrient-based ***Animal-source Fatty Acids*** group);
7) vegetable sources of fats (1 group, the nutrient-based ***Vegetable-source Fatty Acids*** group);
8) legumes, bread, and dairy products (1 group, the food-based ***Traditional Patterns***).

### Single DPs and disease outcomes, drivers, or correlates: statistical analysis

With the exception of 6 [58,67,76,78,79,84] articles, the DPs identified in this systematic review were linked to health/disease outcomes, DP drivers, or correlates. Although one article examined the correlation between DPs and school marks (that is, correlate-oriented research question) [70], the remaining 45 articles employed regression models. Principal component/factor scores were used either as independent variables (for disease-oriented research questions) or as the dependent variable (for driver-oriented research questions) in the regression model. The specific regression model also accounted for the corresponding study design. In the disease-oriented articles, we generally observed that:

1) in longitudinal analyses of cohort studies, Cox proportional hazards models were employed to estimate hazard ratios of disease;
2) in case-control studies, logistic regression models were employed to estimate odds ratios of disease;
3) in cross-sectional studies or in cross-sectional analyses of cohort studies, linear or logistic regression models were employed.

**FIGURE 1:**
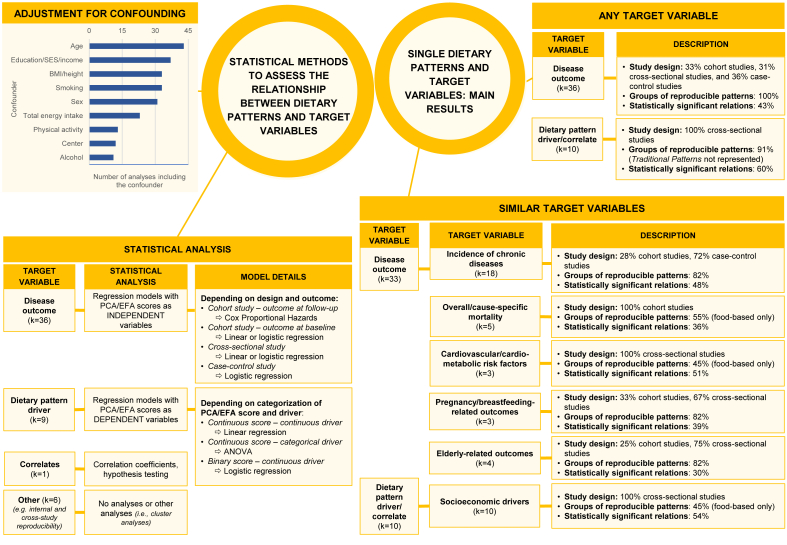
Associations between identified dietary patterns and selected disease outcomes, dietary pattern drivers, and correlates of interest: summary of the second set of findings from the systematic review as presented in this paper. The statistical analysis methods, adjustment for confounding factors, and main results on the associations between single dietary patterns and available target variables were summarized. Abbreviations: ANOVA, analysis of variance; EFA, exploratory factor analysis; PCA, principal component analysis; SES, socioeconomic status.

In the driver-oriented articles, analysis of variance, logistic regression, or linear regression were employed, depending on the categorization used for the principal component/factor scores and the specific driver (Figure 1, left side).

All regression models, except for those applied in 2 articles [55,73], included adjustments for confounding variables. The median number of confounding variables accounted for was 7 (range: 0–14).

Approximately 42% of the 43 articles utilizing multiple regression models included a stratified analysis, typically by age, sex, education, or BMI; heterogeneity across strata was formally tested in only 2 articles [43,45] (Supplemental Table 2).

### Single DPs and disease outcomes, drivers, or correlates: main results

**FIGURE 2:**
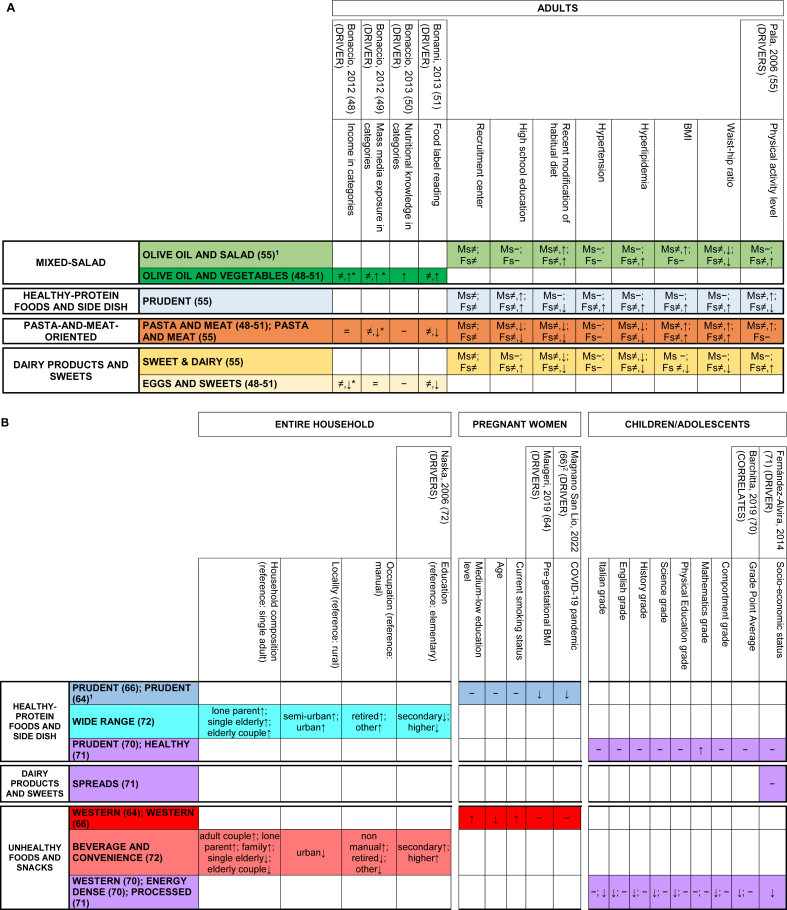
Identified food-based dietary patterns—organized in groups based on text descriptions and original loadings—and related associations with drivers/correlates of interest. Separate panels represented the following target populations: (A) adults and (B) entire household, pregnant women, and children/adolescents.^1,2^^1^In rows, we displayed dietary patterns that look similar (based on text descriptions and original loadings) one close to the other and we consistently indicated them with the same color code. Each row contained dietary patterns showing no or minimal nutritional differences according to their factor loadings; their different names were reported, when present, with the corresponding references, and separated by a “;” symbol; when the dietary pattern name was the same in a row, groups of dietary patterns showing no differences had the corresponding references separated by a “;” symbol. Variants of the same color across rows indicate different subgroups of dietary patterns within the same group, with loadings showing modest but nutritionally relevant differences across color-specific subgroups. Rows left in white indicate patterns that, in our opinion, were too far from any of the previous ones to be indicated as similar to anyone. For additional details on dietary pattern composition we referred to Supplemental Figure 6. Columns related dietary patterns with their drivers/correlates of interest. The background of column headings was color coded to indicate the different study designs and analyses, ranging from trials (black) to cohort studies (dark gray), case-control studies (light gray), and cross-sectional analyses of cohort studies or cross-sectional studies (white); a case-cohort study [57] was identified by a double asterisk in the heading text, together with the dark gray background. Corresponding cells provided significant results from the overall analysis on the main dietary pattern driver/correlate, when identified in the article. In logistic or Cox regression models “↑” indicates a statistically significant risk factor, “↓” indicates a statistically significant protective factor, and “−” indicates a nonstatistically significant association with risk. In analysis of variance models and/or in hypothesis testing alone, “≠” indicates a statistically significant difference in means of the dietary pattern driver/correlate of interest across quantile-based categories of principal component/factor score and “=” indicates the lack of statistically significant difference; when a further analysis investigated the presence of a trend in means, “≠,↑”, “≠,↓”, and “≠,?” indicated an increasing, decreasing, or an unclear trend across quantile-based categories. The addition of an asterisk indicated that a trend was described in the original text, but not formally evaluated from the statistical standpoint. When all the outcomes/correlates were nonsignificantly related with all the identified dietary patterns [53,57], an “−” was indicated to display the corresponding article in the figure. ^2^In Magnano San Lio et al. 2022 article [66], the impact of the COVID-19 pandemic was measured in terms of belonging to the Mamma & Bambino cohort (pregnant women enrolled before the outbreak) or the MAMI-MED cohort (pregnant women enrolled after the outbreak). To make results consistent across articles, we expressed results in terms of COVID-19 pandemic (yes compared with no). Abbreviations: Ca, calcium; Fe, iron; Fs, females; MAMI-MED, Multisettoriale Alla salute Materno-Infantile Mediante valutazione dell’Esposoma nelle Donne; Mg, magnesium; Ms, males; Vit, vitamin; Zn, zinc.

**FIGURE 3:**
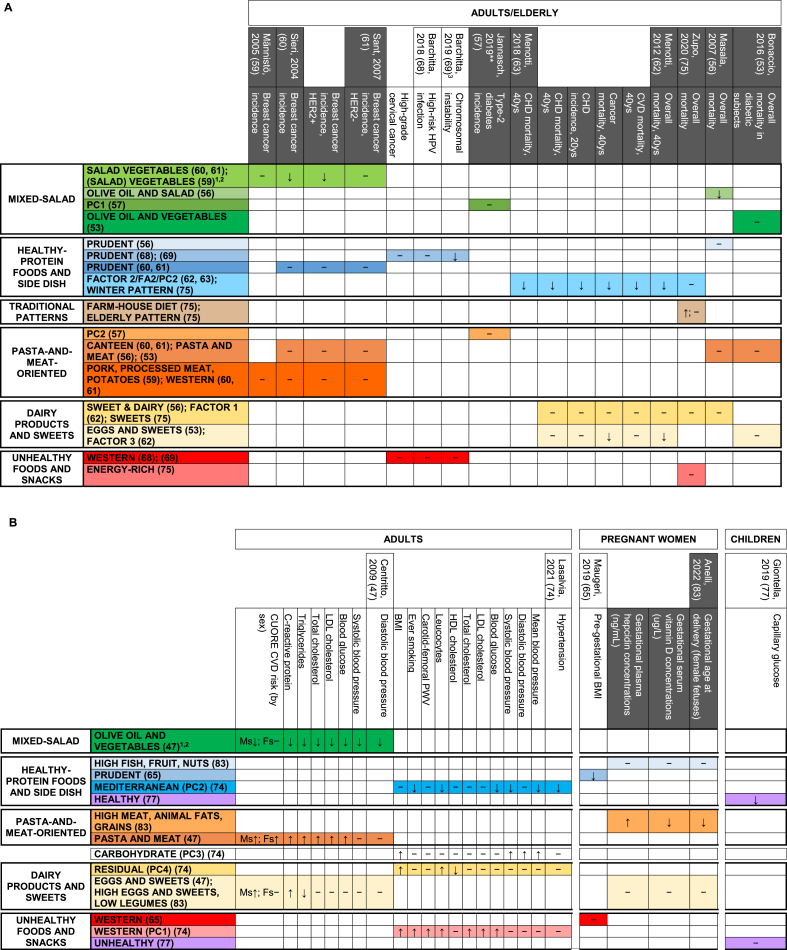

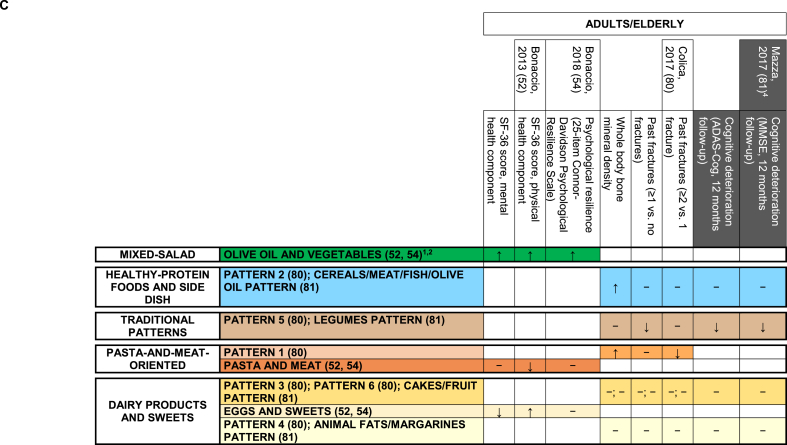
Identified food-based dietary patterns—organized in groups based on text descriptions and original loadings—and related associations with disease outcomes. Separate panels represented the following outcomes: (A) incidence of chronic diseases and overall/cause-specific mortality; (B) cardiovascular/cardiometabolic risk factors and maternal biomarkers, and (C) mental and physical health.^1–4^^1^In rows, we displayed dietary patterns that look similar (based on text descriptions and original loadings) one close to the other and we consistently indicated them with the same color code. Each row contained dietary patterns showing no or minimal nutritional differences according to their factor loadings; their different names were reported, when present, with the corresponding references, and separated by a “;” symbol; when the dietary pattern name was the same in a row, groups of dietary patterns showing no differences had the corresponding references separated by a “;” symbol. Variants of the same color across rows indicate different subgroups of dietary patterns within the same group, with loadings showing modest but nutritionally relevant differences across color-specific subgroups. Rows left in white indicate patterns that, in our opinion, were too far from any of the previous ones to be indicated as similar to anyone. For additional details on dietary pattern composition, we referred to Supplemental Figure 6. Columns related dietary patterns with disease outcomes. The background of column headings was color coded to indicate the different study designs and analyses, ranging from trials (black) to cohort studies (dark gray), case-control studies (light gray), and cross-sectional analyses of cohort studies or cross-sectional studies (white); a case-cohort study [57] was identified by a double asterisk in the heading text, together with the dark gray background. Corresponding cells provided significant results from the overall analysis on the main outcome, when identified in the article. In logistic or Cox regression models, “↑” indicates a statistically significant risk factor, “↓” indicates a statistically significant protective factor, and “−” indicates a nonstatistically significant association with risk. When all the outcomes/correlates were nonsignificantly related with all the identified dietary patterns [53,57], an “−” was indicated to display the corresponding article in the figure. In the lack of further analyses assessing relation with disease outcomes or correlates of interest, an “X” was indicated in the corresponding cell. Results were separately displayed for adults/elderly, pregnant women, and children. ^2^The following dietary patterns snack foods, processed meats and oils [67], legumes, vegetables and fish [67], (salad) vegetables [58], pork, processed meat, potatoes [58], alcohol [58], cooked vegetables [58], DP diabetic [76], DP not diabetic [76], high energy [84], prudent [84], and vegetarian [84], and were not included in the current figure because they were not related to disease outcomes or dietary pattern drivers in the original articles. ^3^In Barchitta et al. 2019 article [69], average methylation of CpG sites within LINE-1 DNA sequences was investigated and reported to be inversely associated with chromosomal instability and aberrant genome function. To make results consistent across articles, we reported results in terms of chromosomal instability. ^4^In Mazza et al. 2017 article [81], higher values of ADAS-cog reflected increasing levels of cognitive deterioration, whereas higher values of MMSE reflected increasing levels of cognitive performance. To make results consistent within and across articles, we adopted “↓” to express a protective effect against cognitive deterioration. Abbreviations: ADAS-Cog, Alzheimer’s Disease Assessment Scale—Cognitive subscale; CAD, coronary artery disease; CVD, cardiovascular disease; DP, dietary pattern; FA, factor analysis (factor name from original articles); Fs, females; HER2, human epidermal growth factor receptor 2; HPV, human papilloma virus; MMSE, Mini-Mental State Examination; Ms, males; PC, principal component analysis (principal component name from original articles); PWV, pulse wave velocity; SF-36, Short Form Healthy Survey 36.

**FIGURE 4:**
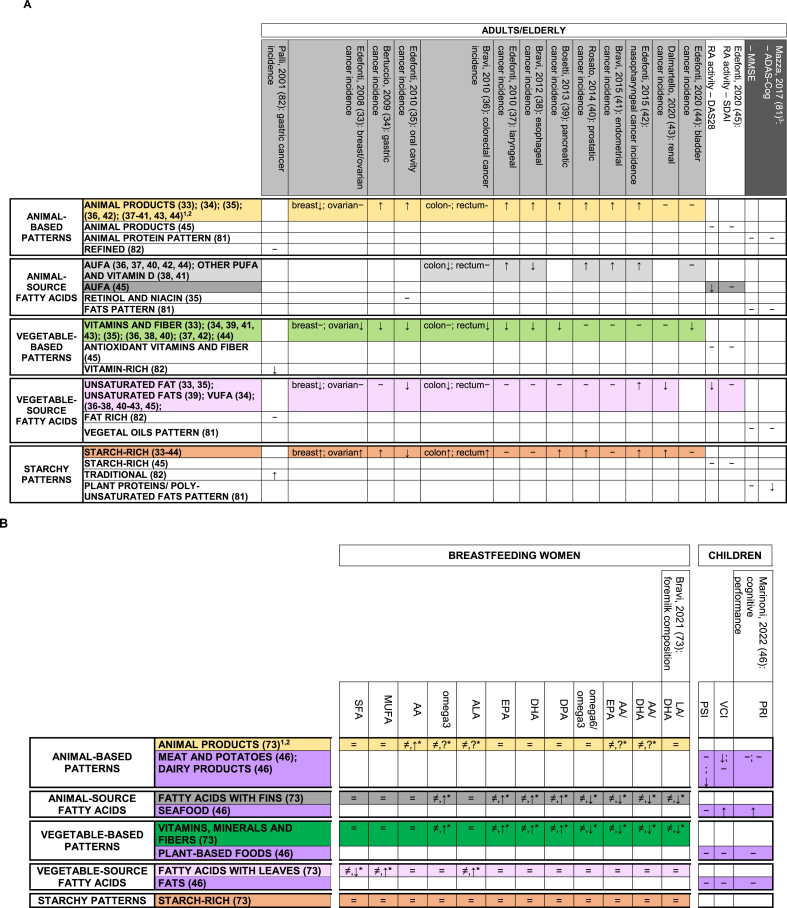
Identified nutrient-based dietary patterns—organized in groups based on text descriptions and original loadings—and related associations with disease outcomes. Separate panels represented the following combinations of target populations: (A) adults/elderly; (B) breastfeeding women and children.^1,2^^1^In rows, we displayed dietary patterns (DPs) that look similar (based on text descriptions and original loadings) one close to the other and we consistently indicated them with the same color code. Each row contained DPs showing no or minimal nutritional differences according to their factor loadings; their different names were reported, when present, with the corresponding references, and separated by a “;” symbol; when the dietary pattern name was the same in a row, groups of DPs showing no differences had the corresponding references separated by a “;” symbol. Variants of the same color across rows indicate different subgroups of DPs within the same group, with loadings showing modest but nutritionally relevant differences across color-specific subgroups. Rows left in white indicate patterns that, in our opinion, were too far from any of the previous ones to be indicated as similar to anyone. For additional details on dietary pattern composition, we referred to Supplemental Figure 6. Columns related DPs with available disease outcomes. The background of column headings was color coded to indicate the different study designs and analyses, ranging from trials (black) to cohort studies (dark gray), case-control studies (light gray), and cross-sectional analyses of cohort studies or cross-sectional studies (white). Corresponding cells provided significant results from the overall analysis on the main outcome, when identified in the article. In logistic or Cox regression models, “↑” indicated a statistically significant risk factor, “↓” indicated a statistically significant protective factor, and “−” indicated a nonstatistically significant association with risk. In ANOVA models and/or in hypothesis testing alone, “≠” indicated a statistically significant difference in means of the outcome/correlate of interest across quantile-based categories of principal component/factor score and “ = ” indicated the lack of statistically significant difference; when a further analysis investigated the presence of a trend in means, “≠,↑”, “≠,↓”, and “≠,?” indicated an increasing, decreasing, or an unclear trend across quantile-based categories. The addition of an asterisk indicated that a trend was described in the original text, but not formally evaluated from the statistical standpoint. ^2^The following DPs animal products [78], vitamins and fiber [78], regional [78], factor 1 [79], factor 2 [79], and factor 3 [79], were not included in the current figure because they were not related to disease outcomes or dietary pattern drivers in the original articles. ^3^In Mazza et al. 2017 article [81], higher values of ADAS-cog reflected increasing levels of cognitive deterioration, whereas higher values of MMSE reflected increasing levels of cognitive performance. To make results consistent within and across articles, we adopted “↓” to express a protective effect against cognitive deterioration. Abbreviations: AA, arachidonic acid; ADAS-cog, Alzheimer’s Disease Assessment Scale—Cognitive subscale; ALA, alpha-linolenic acid; AUFA, animal unsaturated fatty acids; DAS28, Disease Activity Score on 28 joints; DPA, docosapentaenoic acid; LA, linoleic acid; MMSE, Mini-Mental State Examination; PRI, perceptual reasoning index; PSI, processing speed index; RA, rheumatoid arthritis; SDAI, Simplified Disease Activity Index; SFA, saturated fatty acid(s); VCI, verbal comprehension index; VUFA, Vegetable Unsaturated Fatty Acids.

Figure 1 (right side) summarizes the main findings on relationships between single PCA/EFA-based DPs identified in Italy and the corresponding health/disease outcomes, DP drivers, or correlates of interest. Figures 2–**4** provide detailed results by input-variable type for PCA/EFA, target-variable type, population, and study design, for the 61 DPs related to any target variable in the original publications (17 DPs from 6 articles [58,67,76,78,79,84] were not related to any variable). The post-hoc grouping of disease outcomes, DP drivers, or correlates of interest is detailed in Table 1.

**TABLE 1:** Available dietary pattern drivers, correlates of interest, and health or disease outcomes, in groups of similar target variables to facilitate evidence synthesis.

Target variable type	Groups of similar target variables		Same target variable available in ≥2 included articles	Same target variable available for the same group of reproducible dietary patterns
**Disease outcome**	**Incidence of chronic diseases**	Cancer incidence (several sites)	Cancer incidence	Cancer incidence
		CAD incidence	CAD incidence	—
		Type 2 diabetes incidence	—	—
	**Overall andcause-specificmortality**	Overall mortality	Overall mortality	Overall mortality
		CAD mortality	CAD mortality	—
		CVD mortality	—	—
		Cancer mortality	—	—
	**Cardiovascular and/or cardiometabolic risk factors**	Blood glucose	Blood glucose	Blood glucose
		Blood pressure—SBP	Blood pressure—SBP	Blood pressure—SBP
		Blood pressure—DBP	Blood pressure—DBP	Blood pressure—DBP
		Blood pressure—mean	—	—
		Hypertension	—	—
		Inflammatory markers (CRP, leucocytes)	Inflammatory markers (CRP, leucocytes)	—
		Tryglicerides	—	—
		Cholesterol—total	Cholesterol—total	Cholesterol—total
		Cholesterol—LDL	Cholesterol—LDL	Cholesterol—LDL
		Cholesterol—HDL	—	—
		BMI	—	—
		Smoking	—	—
		CUORE CVD risk	—	—
	**Elderly-related outcomes**	Bone mineral density	—	—
		Fractures	—	—
		Cognitive deterioration	—	—
		Psychological resilience	—	—
		Quality of life (physical/mental health)	—	—
	**Pregnancy/breastfeeding-related outcomes**	Pregestational BMI	—	—
		Gestational age at delivery	—	—
		Maternal biomarkers during pregnancy (concentrations of serum vitamin D and plasma hepcidin)	—	—
		Foremilk composition (SFA, MUFA, AA, omega3, ALA, EPA, DHA, DPA)	—	—
	**Childhood-related outcomes**	Cognitive performance	—	—
**Dietary patterndriver/correlate**	**Socioeconomic characteristics**	Education (adults)	Education	Education
		School performance in adolescents (correlate)	—	—
		Socioeconomic status	—	—
		Income	—	—
		Mass media exposure	—	—
		Nutritional knowledge	—	—
		Food label reading	—	—
		Education (household)	Education	Education
		Occupation (household)	—	—
		Locality (household)	—	—
		Composition (household)	—	—
	**Pregnancy-related drivers**	Pregestational BMI	—	—
		Education (pregnancy)	Education	Education
		Age	—	—
		Current smoking status	—	—
		COVID-19 pandemic	—	—
	**Lifestyle drivers**	Physical activity level	—	—
		Dietary modifications	—	—
		Recruitment center	—	—
	**Anthropometric drivers**	BMI	—	—
		Waist–hip ratio	—	—
	**Cardiovascular and/or cardiometabolic drivers**	Hyperlipidemia	—	—
		Hypertension	—	—

Abbreviations: AA, arachidonic acid; ALA, alpha-linolenic acid; CAD, coronary artery disease; CRP, C-reactive protein; CVD, cardiovascular disease; DBP, diastolic blood pressure; DPA, docosapentaenoic acid; SBP, systolic blood pressure; SFA, saturated fatty acid(s).

The evidence on relationships between DPs and any available target variables was described along 455 possible combinations of single DPs and target variables. Of these, 121 combinations concerned DP drivers or correlates, and 334 concerned health/disease outcomes. Statistically significant associations were found in 60% (73/121) of the possible combinations of investigated DPs and any of their drivers/correlates. This percentage increased to 67% (65/97) when we restricted the analysis to drivers only. The identified associations with drivers/correlates were limited to cross-sectional analyses of food-based DPs that did not fall under the ***Traditional Patterns*** group (FIGURE 1, FIGURE 2). Statistically significant associations were found in 42% (139/334) of the possible combinations of identified DPs and any available disease outcomes (food-based DPs: 39% = 68/173; nutrient-based DPs: 44% = 71/161). The identified associations with disease outcomes encompassed all groups of reproducible DPs and available study designs (FIGURE 1, FIGURE 3, FIGURE 4).

When target variables were grouped by similarity, socioeconomic drivers/correlates accounted for 42% (57/121) of combinations regarding DP drivers/correlates. Their group included education, school performance, income, socioeconomic status, mass media exposure, nutritional knowledge and culture, as well as household occupation, composition, and geographical location (Table 1). Statistically significant associations were observed in 54% (31/57) of the combinations of identified DPs and socioeconomic characteristics. When significant, the relationships between the identified DPs and their socioeconomic characteristics followed the expected direction across the various target populations, including children/adolescents [70,71], adults [[48], [49], [50], [51],^64^,^66^,^72^], and the elderly [55]. Specifically, putatively detrimental DPs were related to lower levels of the examined socioeconomic characteristics, whereas putatively protective DPs exhibited the opposite trend (Figure 2A and B, Supplemental Results, text for detailed results). Still, this evidence derived from cross-sectional analyses and included about half of the groups of reproducible DPs (food-based DPs only) (Figure 1). The incidence of chronic diseases, overall/cause-specific mortality, cardiovascular and/or cardiometabolic risk factors, pregnancy/breastfeeding-related outcomes, and elderly-related outcomes accounted for 91% (305/334) of the available combinations concerning health/disease outcomes (Table 1 for details on specific variables). Statistically significant associations were observed in 43% (132/305) of these combinations, including 48% (43/89) for incidence of chronic diseases, 36% (9/25) for overall/cause-specific mortality, 51% (39/77) for cardiovascular and/or cardiometabolic risk factors in adults and children, 39% (28/71) for pregnancy/breastfeeding-related outcomes, and 30% (13/43) for elderly-related outcomes. When associations were statistically significant, groups including potentially detrimental DPs generally presented increased risks of previously mentioned adverse health outcomes. Groups including potentially protective DPs generally revealed the opposite (FIGURE 3, FIGURE 4). Cross-sectional analyses provided all evidence on cardiovascular and/or cardiometabolic risk factors and most evidence from pregnancy/breastfeeding-related and elderly-related outcomes (Figure 1).

### Single DPs and disease outcomes, drivers, or correlates: adjustment for confounding factors

Excluding 2 articles that utilized automatic selection of confounding factors (5 analyses in total) [80,81], all included articles adjusted for age, sex, or center/geographical area, when appropriate. Among socioeconomic factors, education, socioeconomic status, or income were considered either individually or in pairs in 86% of the analyses (37 over 43 analyses appropriately adjusted for these factors). Regarding lifestyle factors, smoking was accounted for in 80% (33/41) of the analyses, followed by physical activity (30% = 13/44, 8 of which from the Moli-sani study), and alcohol consumption (27% = 11/41, 9 of which from case-control studies on diet and cancer at several sites). Anthropometric measures, particularly BMI or height, were included in 75% (33/44) of the analyses. Total energy intake was also adjusted for in 52% of the multiple regression models (23/44, 9 of which from the Moli-sani study). However, 13 case-control studies on diet and cancer at several sites did not adjust for energy intake as DPs were simultaneously entered into the models (Figure 1, left side).

Overall, adjustment for confounding variables in driver-oriented articles included additional socioeconomic characteristics beyond the primary focus (for example, the article on food label use provided analyses adjusted for education, income, and socioeconomic status). Conversely, adjustment in disease-oriented articles predominantly accounted for education as the key socioeconomic driver linking DPs, confounding factors, and health/disease outcomes. The selected confounding factors in both disease-oriented and driver-oriented articles broadly reflected existing evidence (for example, in studies on hormone-related cancer incidence, age at menarche, parity, oral contraceptives, menopausal status, and/or family history were adjusted for) (Supplemental Table 2).

### Reproducible DPs in relation to similar disease outcomes/DP drivers/correlates of interest: main results

**FIGURE 5:**
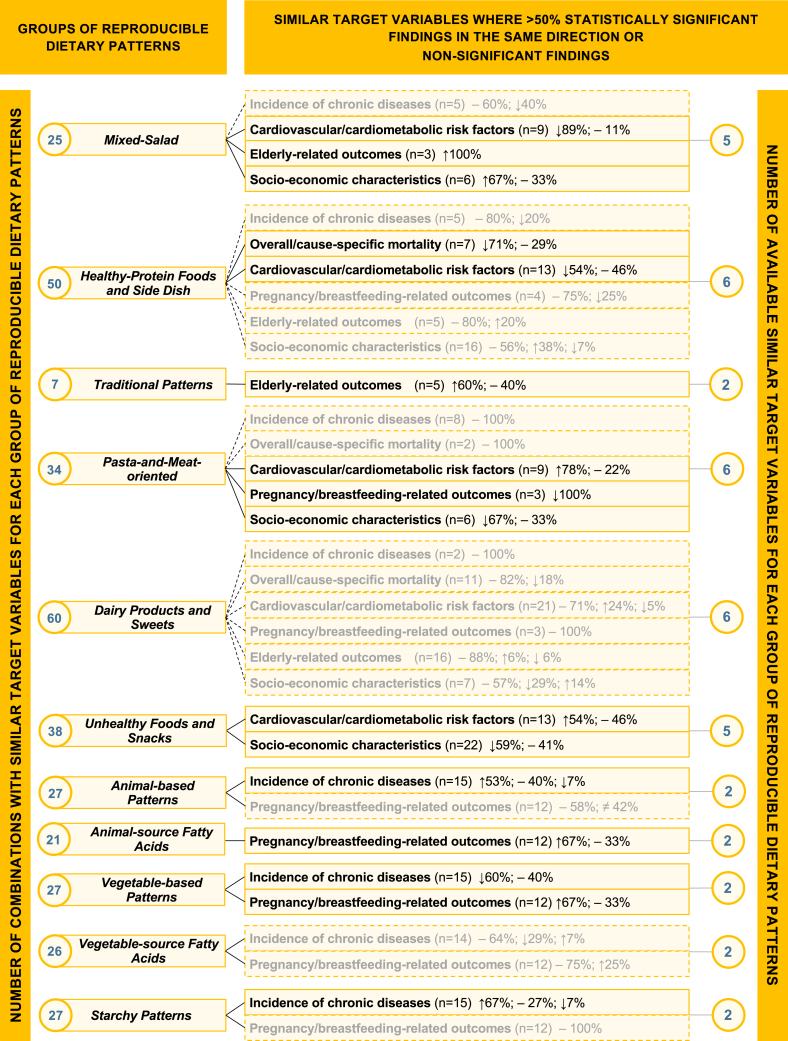
Associations between identified dietary patterns (DPs) and selected disease outcomes, dietary pattern drivers, and correlates of interest: main results on groups of reproducible DPs and similar target variables evaluated using the majority-rules criterion for consistency of the associations.^1,2^^1^For each group of reproducible DPs, we reported the number of available combinations (left side), target variables where either >50% of statistically significant findings going in the same direction or >50% nonsignificant findings were available (central), and the number of available similar target variables (right side). Dashed lines indicate blocks of similar target variables for which >50% nonsignificant findings were identified. The symbol “↑” indicates a statistically significant positive relationship between groups of reproducible DPs and similar/the same target variable, “↓” indicates a statistically significant inverse relationship, “≠” indicates a statistically significant difference, and “−” indicates a nonstatistically significant association. ^2^For the following groups of reproducible DPs, the sum of the combinations indicated for the single groups of similar target variables did not end up into the number of combinations indicated in the left part of the figure: ***Mixed-Salad*** (23 compared with 25), ***Traditional Patterns*** (5 compared with 7), ***Pasta-and-Meat-oriented*** (28 compared with 34), ***Unhealthy Foods and Snacks*** (35 compared with 38), and ***Animal-sourceFatty Acids*** (12 compared with 21). This was due to residual combinations being at 50% significant and 50% nonsignificant findings (***Mixed-Salad***, ***Traditional Patterns***, and ***Pasta-and-Meat-oriented*** groups), or targeting one variable alone (***Unhealthy Foods and Snacks*** group, 3 target variables) or not reaching 50% (***Animal-source Fatty Acids*** group). Abbreviations: PCA, principal component analysis; EFA, exploratory factor analysis.

Combinations of reproducible DPs and similar target variables where >50% of the relationships were either statistically significant and consistent in direction or nonsignificant were reported as follows (Figure 5):

1) the ***Mixed-Salad*** group was positively associated with socioeconomic characteristics in 67% of the combinations (4/6, compared with 2/6 nonsignificant findings) (Figure 2A). It was inversely related to cardiovascular and/or cardiometabolic risk factors in adults (89% = 8/9 compared with 1/9 nonsignificant findings) (Figure 3B). It showed protective effects for elderly-related outcomes (that is, psychological resilience and quality of life) in 100% (3/3) of the combinations (Figure 3C) (15/25 significant associations with similar target variables);
2) the ***Healthy-Protein*** ***Foods and Side Dish*** group showed protective effects against overall/cause-specific mortality in 71% of the combinations (5/7, compared with 2/7 nonsignificant findings) (Figure 3A), and against cardiovascular and/or cardiometabolic risk factors in 54% of the combinations including both adults and children (7/13, compared with 6/13 nonsignificant findings) (Figure 3B) (12/50 significant associations with similar target variables);
3) the ***Traditional Patterns*** group showed protective effects against elderly-related outcomes (that is, cognitive deterioration and the risk of fractures) in 60% of the combinations (3/5 compared with 2/5 nonsignificant findings) (Figure 3C) (3/7 significant associations with similar target variables);
4) the ***Pasta-and-Meat-oriented*** group was related to poorer socioeconomic characteristics (67% = 4/6 of the combinations, adults only, compared with 2/6 nonsignificant findings) (Figure 2A). It was positively related to cardiovascular and/or cardiometabolic risk factors (78% = 7/9 of the combinations, adults only, compared with 2/9 nonsignificant findings) and poorer pregnancy/breastfeeding-related outcomes (100% = 3/3 combinations) (Figure 3B) (14/34 significant associations with similar target variables);
5) the ***Dairy Products and Sweets*** group was primarily nonsignificantly related to the corresponding health/disease outcomes (incidence of chronic diseases (that is, coronary artery disease incidence, 2/2), overall/cause-specific mortality (9/11 nonsignificant compared with 2/11 significantly protective findings), cardiovascular and/or cardiometabolic risk factors (15/21 nonsignificant compared with 5/21 significantly at-risk and 1/21 significantly protective findings), pregnancy/breastfeeding-related outcomes (3/3 nonsignificant findings), elderly-related outcomes (14/16 nonsignificant compared with 1/16 significantly at-risk and 1/16 significantly protective findings)) across both adults and the elderly (Figure 3A–C) and nonsignificantly related to socioeconomic characteristics across both adults and children/adolescents (Figure 2A and B) (4/7 nonsignificant compared with 2/7 inversely-related and 1/7 positively-related findings), giving a total of 47/60 = 78% nonsignificant associations with similar target variables;
6) the ***Unhealthy Foods and Snacks*** group was related with poorer socioeconomic characteristics in adults (including pregnant women), children/adolescents, and the entire household (59% = 13/22 of the combinations compared with 9/22 nonsignificant findings) (Figure 2B). It was positively related with cardiovascular and/or cardiometabolic risk factors in adults (54% = 7/13 of the combinations compared with 6/13 nonsignificant findings) (Figure 3B) (20/38 significant associations with similar target variables);
7) the ***Animal-based*** ***Patterns*** group was associated with an increased risk of chronic diseases (that is, cancer incidence) in adults (53% = 8/15 of the combinations compared with 6/15 nonsignificant and 1/15 protective findings) (Figure 4A) (8/27 significant associations with similar target variables);
8) the ***Animal-source*** ***Fatty Acids*** group improved pregnancy/breastfeeding-related outcomes (that is, foremilk composition in breastfeeding women) (67% = 8/12 of the combinations compared with 4/12 nonsignificant findings) (Figure 4B) (8/21 significant associations with similar target variables);
9) the ***Vegetable-based*** ***Patterns*** group was associated with a decreased risk of chronic diseases (that is, cancer incidence) in adults (60% = 9/15 of the combinations compared with 6/15 nonsignificant findings) (Figure 4A) and improved pregnancy/breastfeeding-related outcomes (that is, foremilk composition in breastfeeding women) (67% = 8/12 of the combinations compared with 4/12 nonsignificant findings) (Figure 4B) (17/27 significant associations with similar target variables);
10) the ***Vegetable-source*** ***Fatty Acids*** group was primarily nonsignificantly associated with the corresponding outcomes (incidence of chronic diseases (that is, cancer incidence, 9/14 nonsignificant compared with 4 significantly protective and 1 significantly at-risk findings) and pregnancy/breastfeeding-related outcomes (that is, foremilk composition, 9/12 nonsignificant compared with 3 significantly protective findings)) across adults, breastfeeding women, and children (Figure 4A and B) (18/26 nonsignificant associations with similar target variables);
11) the ***Starchy Patterns*** group was associated with an increased risk of chronic diseases (that is, cancer incidence) in adults (67% = 10/15 of the combinations, compared with 4/15 nonsignificant and 1/15 significant protective findings) (Figure 4A) (10/27 significant associations with similar target variables).

In total, statistically significant associations were found in 46% (157/342) of the combinations of groups of reproducible DPs and similar target variables.

### Reproducible DPs in relation to the same disease outcomes/DP drivers/correlates of interest: main results

Within this review, groups of reproducible DPs were available for the following disease outcomes/DP drivers: education, cancer incidence, overall mortality, systolic and diastolic blood pressure, total and LDL cholesterol, and glucose (Table 1).

Among DP drivers, education was inconsistently related with 2 DPs from the ***Unhealthy Foods and Snacks*** group (1 significantly at-risk and 1 significantly protective finding) and 3 DPs from the ***Healthy-Protein*** ***Foods and Side Dish*** group (1 significantly at-risk, 1 significantly protective, and 1 nonsignificant finding) (Figure 2A and B). The median number of DPs per group for education was, therefore, 2.5.

Among disease outcomes and related factors, cancer incidence significantly:

1) increased for increasing scores of 53% (8/15) of the nutrient-based DPs from the ***Animal-based*** ***Patterns*** group—compared with 40% (6/15) of nonsignificant findings and 1 significant protective finding (1/15∼7%) (Figure 4A)—in the absence of information from the corresponding ***Dairy Products and Sweets*** group of food-based DPs (Figure 3A);
2) increased for increasing scores of 67% (10/15) of the nutrient-based DPs from the ***Starchy Patterns*** group—compared with 27% (4/15) of nonsignificant findings and 1 significant protective finding (1/15∼7%) (Figure 4A)—in contrast to the 100% (7/7) nonsignificant findings recorded for food-based DPs in the corresponding ***Pasta-and-Meat-oriented*** group (Figure 3A);
3) decreased for increasing scores of 60% (9/15) of the nutrient-based DPs from the ***Vegetable-based*** ***Patterns*** group (Figure 4A)—compared with 40% (6/15) of nonsignificant findings—in the lack of consistent results for the food-based DPs in the corresponding ***Mixed-Salad*** group (2 significantly protective findings, 2 nonsignificant findings) (Figure 3A).

Evidence on cancer incidence was inconsistent for the reproducible nutrient-based DPs in the ***Animal-source*** ***Fatty Acids*** group (4 significantly at-risk compared with 2 significantly protective and 3 nonsignificant findings, out of 9 available combinations) (Figure 4A). It was also nonsignificant for the reproducible nutrient-based DPs in the ***Vegetable-source*** ***Fatty Acids*** group (9/14∼64% compared with 4 significantly protective and 1 significantly at-risk findings) (Figure 4A), and for the reproducible food-based DPs in the ***Healthy-Protein*** ***Foods and Side Dish*** group (4/4 = 100%) (Figure 3A). Finally, the median number of DPs per group was 9 for cancer incidence.

The overall mortality risk was significantly reduced in 50% (1/2) of food-based DPs from the ***Mixed-Salad*** group, in 33% (1/3) of food-based DPs from the ***Healthy-Protein*** ***Foods and Side Dish*** group, and in 20% (1/5) of food-based DPs from the ***Dairy Products and Sweets*** group, whereas all the remaining DPs from previous groups showed nonsignificant relations with risk. All DPs from the ***Pasta-and-Meat-oriented*** group (2/2) were consistently unrelated to overall mortality. The median number of DPs per group was 2.5 for overall mortality (Figure 3A).

Among cardiovascular and/or cardiometabolic risk factors, increasing scores in 100% (2/2) of the reproducible food-based DPs from the ***Healthy-Protein*** ***Foods and Side Dish*** group significantly decreased blood and capillary glucose, in adults and children, respectively. Conversely, the evidence for 2 food-based DPs from the ***Unhealthy Foods and Snacks*** group and the same outcomes was inconsistent (significant detrimental effect in adults, not significant in children). Additionally, the relationship between reproducible food-based DPs from the ***Dairy Products and Sweets*** group and (systolic and diastolic) blood pressure, total and LDL cholesterol, and blood glucose yielded consistent nonsignificant findings across both DPs for each outcome investigated, resulting in 100% (10/10) nonsignificant findings. The median number of DPs per group was 2 for each of the examined risk factors (Figure 3A).

Notably, 38 out of 45 combinations also reflected >50% nonsignificant findings across the same disease outcomes (cancer incidence: 7/7, 9/14, and 4/4; overall mortality: 2/3, 4/5, 2/2; cardiovascular and cardiometabolic risk factors: 10/10). When considering all the same target variables together, the median number of DPs per group of reproducible DPs was equal to 2.

### Reproducible DPs in relation to the same disease outcomes/DP drivers/correlates of interest: evaluation of causal criteria and rules of inference

On the basis of 15 articles, 53 associations involving 9 groups of DPs and 9 disease outcomes/DP drivers were potentially suitable for meta-analysis, with a median number of 2 DPs per target variable (IQR: 2–2.25) (Table 2). Within each reproducible DP-target variable combination, the study designs were consistent—with cohort studies available for breast cancer incidence and overall mortality—and confounding was generally accounted for, except for 1 article [55]. Control for confounding generally included age, sex, and total energy intake, when considering individual DPs. Except in 1 article [74], additional confounders were present, including education (rarely, social class, or socioeconomic status) and BMI (rarely, height, weight, or waist), in 87% of the studies. The association between education and 2 groups of reproducible DPs was investigated in 3 cross-sectional studies including pregnant women, the elderly, and the entire household, with no [55] or various [64,72] control for confounding, and different types of effect estimates. For breast cancer incidence, data from independent studies were unavailable to carry out meta-analyses, despite the cohort design and control for confounding being appropriate [[59], [60], [61]]. The 4 potential meta-analyses on gastric cancer incidence were based on 2 estimates, each derived from case-control studies with similar adjustment for confounding factors, including family history of gastric cancer [34,82]. Similarly, pairwise comparisons were available for glucose (3 groups of DPs), systolic and diastolic blood pressure (1 group of DPs for each risk factor), and total and LDL cholesterol (1 group of DPs for each risk factor), derived from 3 cross-sectional studies with inadequate [74] or similar [47,77] control for confounding. The comparisons including DPs from the ***Healthy-Protein*** ***Foods and Side Dish*** group showed statistically significant inverse associations with glucose, although based on differential adjustment for confounders. Overall mortality was associated with 4 groups of reproducible DPs [53,56,62,75]. Two groups, ***Mixed-Salad*** and ***Pasta-and-Meat-oriented***, each included 2 comparisons based on elderly and diabetic populations, respectively. The remaining 2 groups involved 3 and 5 comparisons. The ***Healthy-Protein*** ***Foods and Side Dish*** group covered multiple populations, including adult men, the elderly, and the general population, with 1 article focusing on 40-y mortality. Similarly, the ***Dairy Products and Sweets*** group included 2 DPs from the same study on adult men (with 40-y mortality as the outcome), 1 DP from diabetic subjects, 1 from the elderly, and 1 from the overall population. Due to insufficient population comparability and a limited number of comparisons, reliable meta-analyses and conclusions on the “consistency of associations” with the same target variables were not possible. Nonetheless, the “strength of association” criterion was met for the ***Vegetable-based*** ***Patterns*** group, which consistently showed odds ratios of gastric cancer risk around 0.5 and corresponding confidence intervals not including 1, although without a consistent linear trend for satisfying the “dose–response” criteria [34,82]. In addition, the ***Starchy Patterns*** group showed significant odds ratios >1.5 and a linear trend across quantiles, thus satisfying the “dose–response” criterion too [34,82].

**TABLE 2:** Associations between groups of reproducible dietary patterns and the same disease outcomes or dietary pattern drivers: evidence for assessing causal criteria.

Same target variable available for the same group of reproducible dietary patterns	Group of reproducible dietary patterns	Dietary pattern	First author's name	Study design/analysis	Confounding	Associations with disease outcomes or dietary pattern drivers
**Cancer incidence—breast cancer**	***Mixed-Salad***	*Salad Vegetables*	Sieri, 2004 [60]	Cohort, adult women	Adjusted for EI, age, years of education, parity, height, age at menarche, smoking, and menopausal status	RR: 0.66 (95% CI: 0.47, 0.95, *P*-trend = 0.016) for 3rd compared with 1st tertile of factor scores
		*Salad Vegetables*	Sant, 2007 [61]	Cohort, adult women, reanalysis of Sieri, 2004	Adjusted for total EI, age, years of education, parity, height, weight, age at menarche, smoking, and menopausal status	HER2–: RR: 0.71 (95% CI: 0.48, 1.03, *P*-trend = 0.072) for 3rd compared with 1st tertile of factor scores
						HER2+: RR: 0.25 (95% CI: 0.10, 0.64, *P*-trend = 0.001) for 3rd compared with 1st tertile of factor scores
		*(Salad) Vegetables*	Männistö, 2005 [59]	Cohort, adult women	Adjusted for age, BMI, height, education, smoking status, family history of breast cancer, OC and HRT use, alcohol intake, and EI	RR: 0.79 (95% CI: 0.50, 1.27, *P*-trend = 0.32) for 4th compared with 1st quartile of factor scores
	***Healthy-Protein Foods and Side Dish***	*Prudent*	Sieri, 2004 [60]	Cohort, adult women	Adjusted for EI, age, years of education, parity, height, age at menarche, smoking, and menopausal status	RR: 1.28 (95% CI: 0.90, 1.83, *P*-trend = 0.169) for 3rd compared with 1st tertile of factor scores
		*Prudent*	Sant, 2007 [61]	Cohort, adult women, reanalysis of Sieri, 2004	Adjusted for total EI, age, years of education, parity, height, weight, age at menarche, smoking, and menopausal status	HER2-: RR: 1.36 (95% CI: 0.93, 1.98, *P*-trend = 0.126) for 3rd compared with 1st tertile of factor scores
						HER2+: RR: 0.72 (95% CI: 0.35, 1.48, *P*-trend = 0.372) for 3rd compared with 1st tertile of factor scores
	***Pasta-and-Meat-oriented***	*Canteen*	Sieri, 2004 [60]	Cohort, adult women	Adjusted for EI, age, years of education, parity, height, age at menarche, smoking, and menopausal status	RR: 0.95 (95% CI: 0.63, 1.45, *P*-trend = 0.935) for 3rd compared with 1st tertile of factor scores
		*Canteen*	Sant, 2007 [61]	Cohort, adult women, reanalysis of Sieri, 2004	Adjusted for total EI, age, years of education, parity, height, weight, age at menarche, smoking, and menopausal status	HER2-: RR: 1.14 (95% CI: 0.75, 1.75, *P*-trend = 0.520) for 3rd compared with 1st tertile of factor scores
						HER2+: RR: 1.39 (95% CI: 0.50, 3.84, *P*-trend = 0.530) for 3rd compared with 1st tertile of factor scores
		*Western*	Sieri, 2004 [60]	Cohort, adult women	Adjusted for EI, age, years of education, parity, height, age at menarche, smoking, and menopausal status	RR: 0.90 (95% CI: 0.58, 1.41, *P*-trend = 0.705) for 3rd compared with 1st tertile of factor scores
		*Western*	Sant, 2007 [61]	Cohort, adult women, reanalysis of Sieri, 2004	Adjusted for total EI, age, years of education, parity, height, weight, age at menarche, smoking, and menopausal status	HER2-: RR: 0.88 (95% CI: 0.55, 1.40, *P*-trend = 0.651) for 3rd compared with 1st tertile of factor scores
						HER2+: RR: 0.75 (95% CI: 0.27, 2.08, *P*-trend = 0.584) for 3rd compared with 1st tertile of factor scores
		*Pork, processed meat, potatoes*	Männistö, 2005 [59]	Cohort, adult women	Adjusted for age, BMI, height, education, smoking status, family history of breast cancer, OC and HRT use, alcohol intake, and EI	RR: 1.07 (95% CI: 0.58, 1.98, *P*-trend = 0.95) for 4th compared with 1st quartile of factor scores
**Cancer incidence—Gastric cancer**	***Animal-based Patterns***	*Animal products*	Bertuccio, 2009 [34]	Case-control	Conditioned on age and sex; adjusted for quinquennia of period of interview, education, BMI, tobacco smoking, and family history of gastric cancer	OR 2.13 (95% CI: 1.34, 3.40, *P*-trend = 0.0003) for 4th compared with 1st quartile of factor scores
		*Refined*	Palli, 2001 [82]	Case-control	Adjusted for age, sex, social class, family history of gastric cancer, area of residence, BMI tertiles, and EI	OR 1.2 (95% CI: 0.8, 1.7, *P*-trend = 0.04) for 3rd compared with 1st tertile of factor scores
	***Vegetable-based Patterns***	*Vitamins and fiber*	Bertuccio, 2009 [34]	Case-control	Conditioned on age and sex; adjusted for quinquennia of period of interview, education, BMI, tobacco smoking, and family history of gastric cancer	OR 0.60 (95% CI: 0.37, 0.99, *P*-trend = 0.0861) for 4th compared with 1st quartile of factor scores
		*Vitamin-rich*	Palli, 2001 [82]	Case-control	Adjusted for age, sex, social class, family history of gastric cancer, area of residence, BMI tertiles, and EI	OR 0.5 (95% CI: 0.4, 0.7, *P*-trend = 0.0003) for 3rd compared with 1st tertile of factor scores
	***Vegetable-source Fatty Acids***	*Vegetable unsaturated fatty acids*	Bertuccio, 2009 [34]	Case-control	Conditioned on age and sex; adjusted for quinquennia of period of interview, education, BMI, tobacco smoking, and family history of gastric cancer	OR 0.89 (95% CI: 0.56, 1.42, *P*-trend = 0.7325) for 4th compared with 1st quartile of factor scores
		*Fat-rich*	Palli, 2001 [82]	Case-control	Adjusted for age, sex, social class, family history of gastric cancer, area of residence, BMI tertiles, and EI	OR 0.8 (95% CI: 0.5, 1.1, *P*-trend = 0.2) for 3rd compared with 1st tertile of factor scores
	***Starchy Patterns***	*Starch-rich*	Bertuccio, 2009 [34]	Case-control	Conditioned on age and sex; adjusted for quinquennia of period of interview, education, BMI, tobacco smoking, and family history of gastric cancer	OR 1.67 (95% CI: 1.01, 2.77, *P*-trend = 0.0463) for 4th compared with 1st quartile of factor scores
		*Traditional*	Palli, 2001 [82]	Case-control	Adjusted for age, sex, social class, family history of gastric cancer, area of residence, BMI tertiles, and EI	OR 3 (95% CI: 1.8, 4.8, *P*-trend = 0.0001) for 3rd compared with 1st tertile of factor scores
**Blood and capillary glucose**	***Healthy-Protein Foods and Side Dish***	*Mediterranean (PC2)*	Lasalvia, 2021 [74]	Cross-sectional	Adjusted for age, sex, and total EI	Mean difference between 5th and 1st quintile category of factor scores was significantly different from 0 for glucose (-3.03, 95% CI: –5.08, –0.98)
		*Healthy*	Giontella, 2019 [77]	Cross-sectional, children	Adjusted for age, sex, ethnicity, BMI, quartiles of total EI, and quartiles of Children-Physical Activity Questionnaire scores	Capillary glucose was inversely associated with factor scores (beta = –0.016, 95% CI: –0.027 , –0.005, *P*-value < 0.01)
	***Dairy Products and Sweets***	*Residuals (PC4)*	Lasalvia, 2021 [74]	Cross-sectional	Adjusted for age, sex, and total EI	Mean difference between 5th and 1st quintile category of factor scores was not significantly different for blood glucose (beta = 0.57, 95% CI: 1.29, 2.42)
		*Eggs and sweets*	Centritto, 2009 [47]	Cross-sectional	Adjusted for sex, smoking, SES, age, BMI, total EI, and total PA (continuous)	Higher factor scores were not associated with blood glucose (95.0 mg/dL, 95% CI 94.3, 95.6, *P*-trend = 0.2)
	***Unhealthy Foods and Snacks***	*Western (PC1)*	Lasalvia, 2021 [74]	Cross-sectional	Adjusted for age, sex, and total EI	Mean difference between 5th and 1st quintile category of factor scores was significantly different from 0 for glucose (4.54, 95% CI: 2.22, 6.86)
		*Unhealthy*	Giontella, 2019 [77]	Cross-sectional, children from 3rd and 4th class of the primary school	Adjusted for age, sex, ethnicity, BMI, quartiles of total EI, and quartiles of Children-Physical Activity Questionnaire scores	Nonsignificantly associated in regression models but estimates were not reported
**Blood pressure—SBP**	***Dairy Products and Sweets***	*Residuals (PC4)*	Lasalvia, 2021 [74]	Cross-sectional	Adjusted for age, sex, and total EI	Mean difference between 5th and 1st quintile category of factor scores was not significantly different for SBP (beta = –1.03, 95% CI: –3.19, 1.13)
		*Eggs and sweets*	Centritto, 2009 [47]	Cross-sectional	Adjusted for sex, smoking, SES, age, BMI, total EI, and total PA (continuous)	Higher factor scores were not associated with SBP (mean: 135.9 mm/Hg SEM: 0.4, *P*-trend = 0.8)
**Blood pressure—DBP**	***Dairy Products and Sweets***	*Residuals (PC4)*	Lasalvia, 2021 [74]	Cross-sectional	Adjusted for age, sex, and total EI	Mean difference between 5th and 1st quintile category of factor scores was not significantly different for SBP (beta = –0.41, 95% CI: –1.60, 0.79)
		*Eggs and sweets*	Centritto, 2009 [47]	Cross-sectional	Adjusted for sex, smoking, SES, age, BMI, total EI, and total PA (continuous)	Higher factor scores were not associated with DBP (mean: 81.6 mm/Hg SEM: 0.2, *P*-trend = 0.5)
**Cholesterol—total**	***Dairy Products and Sweets***	*Residuals (PC4)*	Lasalvia, 2021 [74]	Cross-sectional	Adjusted for age, sex, and total EI	Mean difference between 5th and 1st quintile category of factor scores was not significantly different for SBP (beta = –3.36, 95% CI: –8.03, 1.32)
		*Eggs and sweets*	Centritto, 2009 [47]	Cross-sectional	Adjusted for sex, smoking, SES, age, BMI, total EI and total PA (continuous)	Higher factor scores were not associated with total cholesterol (mean: 215.1 mg/dL SEM: 1.1, *P*-trend = 0.85)
**Cholesterol—LDL**	***Dairy Products and Sweets***	*Residuals (PC4)*	Lasalvia, 2021 [74]	Cross-sectional	Adjusted for age, sex, and total EI	Mean difference between 5th and 1st quintile category of factor scores was not significantly different for SBP (beta = –0.29, 95% CI: –4.59, 4.00)
		*Eggs and sweets*	Centritto, 2009 [47]	Cross-sectional	Adjusted for sex, smoking, SES, age, BMI, total EI, and total PA (continuous)	Higher factor scores were not associated with LDL cholesterol (mean: 132.4 mg/dL SEM: 1.0, *P*-trend = 0.55)
**Overall mortality**	***Mixed-Salad***	*Olive oil and salad*	Masala, 2007 [56]	Cohort, elderly	Adjusted for sex, age, log-transformed EI, BMI, waist, smoking status, years of education, civil status, hypertension status at enrolment, and PAL	HR: 0.50 (95% CI: 0.29, 0.86, *P*-trend = 0.02) for 4th compared with 1st quartile of factor scores
		*Olive oil and vegetables*	Bonaccio, 2016 [53]	Cohort, diabetic subjects	Adjusted for age, sex, education, EI, leisure-time PA, smoking, years from diagnosis of diabetes, blood glucose levels, and hypercholesterolemia	HR: 0.81 (95% CI: 0.62, 1.07) for 1 SD increase in factor scores
	***Healthy-Protein Foods and Side Dish***	*Prudent*	Masala, 2007 [56]	Cohort, elderly	Adjusted for sex, age, log-transformed EI, BMI, waist, smoking status, years of education, civil status, hypertension status at enrolment, and PAL	HR: 0.85 (95% CI: 0.47, 1.53, *P*-trend = 0.95) for 4th compared with 1st quartile of factor scores
		*Factor 2*	Menotti, 2012 [62]	Cohort, adult men, 40-y mortality	Adjusted for age, BMI, smoking status, SBP, and serum cholesterol	HR: 0.89 (95% CI: 0.83, 0.96) for 1 SD increase in factor scores
		*Winter pattern*	Zupo, 2020 [75]	Cohort	Adjusted for sex, age, BMI, education level, smoking, multimorbidity, wine consumption, and olive oil consumption	HR: 0.97 (95% CI: 0.92, 1.02)
	***Pasta-and-Meat-oriented***	*Pasta and meat*	Bonaccio, 2016 [53]	Cohort, diabetic subjects	Adjusted for age, sex, education, EI, leisure-time PA, smoking, years from diagnosis of diabetes, blood glucose levels, and hypercholesterolemia	HR: 0.96 (95% CI: 0.69, 1.32) for 1 SD increase in factor scores
		*Pasta and meat*	Masala, 2007 [56]	Cohort, elderly	Adjusted for sex, age, log-transformed EI, BMI, waist, smoking status, years of education, civil status, hypertension status at enrolment, and PAL	HR: 1.37 (95% CI: 0.80, 2.34, *P*-trend = 0.34) for 4th compared with 1st quartile of factor scores
	***Dairy Products and Sweets***	*Sweet and dairy*	Masala, 2007 [56]	Cohort, elderly	Adjusted for sex, age, log-transformed EI, BMI, waist, smoking status, years of education, civil status, hypertension status at enrolment, and PAL	HR: 1.47 (95% CI: 0.85, 2.54, *P*-trend = 0.25) for 4th compared with 1st quartile of factor scores
		*Factor 1*	Menotti, 2012 [62]	Cohort, adult men, 40-y mortality	Adjusted for age, BMI, smoking status, SBP, and serum cholesterol	HR: 1.00 (95% CI: 0.94, 1.06) for 1 SD increase in factor scores
		*Sweets*	Zupo, 2020 [75]	Cohort	Adjusted for sex, age, BMI, education level, smoking, multimorbidity, wine consumption, and olive oil consumption	HR: 1.01 (95% CI: 0.95, 1.08)
		*Eggs and sweets*	Bonaccio, 2016 [53]	Cohort, diabetic subjects	Adjusted for age, sex, education, EI, leisure-time PA, smoking, years from diagnosis of diabetes, blood glucose levels, and hypercholesterolemia	HR: 1.34 (95% CI: 0.98, 1.83) for 1 SD increase in factor scores
		*Factor 3*	Menotti, 2012 [62]	Cohort, adult men, 40-y mortality	Adjusted for age, BMI, smoking status, SBP, and serum cholesterol	HR: 0.93 (95% CI: 0.97, 1.00, as reported in the article) for 1 SD increase in factor scores
**Education**	***Healthy-Protein Foods and Side Dish***	*Prudent*	Pala, 2006 [55]	Cross-sectional, elderly	Not applicable	Different crude mean factor scores among Ms with (0.13) and without (–0.05) high school education (*P*-value = 0.002) and Fs with (0.26) and without (–0.08) high school education (*P*-value <0.001)
		*Prudent*	Maugeri, 2019 [64]	Cross-sectional, pregnant women	(Mutually) adjusted for age, education level, employment status, smoking, pregestational BMI, use of folic acid supplements and use of multivitamin and/or multimineral supplements	OR 0.70 (95% CI: 0.35, 1.42; *P*-value = 0.322) for 3rd compared with 1st tertile of factor score
		*Wide range*	Naska, 2006 [72]	Cross-sectional, household	(Mutually) adjusted for education level, locality, occupation, and household composition	Compared with elementary education, secondary (beta: –0.22, 95% CI: –0.30, –0.14) or higher (beta: –0.40, 95% CI: –0.55, –0.25) education was inversely related to factor scores
	***Unhealthy Foods and Snacks***	*Western*	Maugeri, 2019 [64]	Cross-sectional, pregnant women	(Mutually) adjusted for age, education level, employment status, smoking, pregestational BMI, use of folic acid supplements and use of multivitamin and/or multimineral supplements	Being in the 3rd factor score tertile was directly associated with medium-low education level (OR 1.617, 95% CI: 1.006, 3.374; *P* = 0.047)
		*Beverage and convenience*	Naska, 2006 [72]	Cross-sectional, household	(Mutually) adjusted for education level, locality, occupation, and household composition	Compared with elementary education, secondary (beta: 0.21, 95% CI: 0.16, 0.25) or higher (beta: 0.24, 95% CI: 0.15, 0.32) education was directly related to factor scores

Abbreviations: CI, confidence interval; DBP, diastolic blood pressure; EI, energy intake(s); HER2, human epidermal growth factor receptor 2; HR, hazard ratio; HRT, hormone replacement therapy; M: male; OC, oral contraceptive; OR, odds ratio; PA, physical activity; PAL, physical activity level; PC, principal component; RR, relative risk; SBP, systolic blood pressure; SES, socioeconomic status.

### Overview of results from the systematic review on reproducibility of DPs in Italy and their consistent associations with disease outcomes, DP drivers, or correlates

**FIGURE 6:**
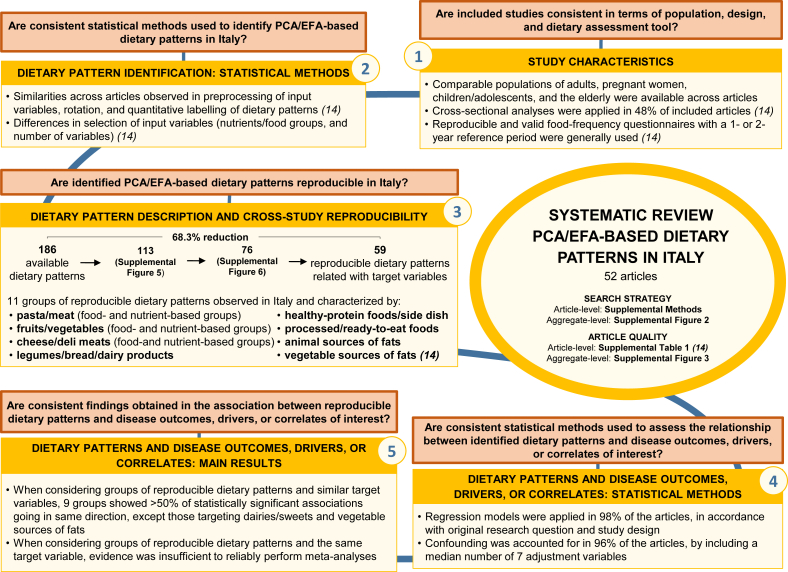
Specific research questions and corresponding findings from the systematic review on a posteriori dietary patterns (DPs) identified with principal component analysis and/or exploratory factor analysis in Italy and their associations with disease outcomes, dietary pattern drivers, or correlates of interest.^1^^1^In the blocks, we summarized the findings from the systematic review concerning the following aspects: study characteristics, dietary pattern identification method, dietary pattern description and cross-study reproducibility, statistical methods used to assess the relationships between identified DPs and disease outcomes/drivers/correlates of interest, and the main results concerning these relationships. The specific research questions were summarized at the top of each box and presented in a logical flow (indicated by a solid arrow), to highlight how all research questions contributed to the final evaluation of the consistency of associations between identified DPs and disease outcomes/dietary pattern drivers/correlates of interest. Abbreviations: PCA, principal component analysis; EFA, exploratory factor analysis.

The collected evidence from this systematic review on PCA/EFA-based DPs identified in Italy was utilized to address 2 methodological questions regarding: *1*) their cross-study reproducibility, and *2*). the consistency of their associations with disease outcomes/DP drivers/correlates of interest. We observed the following regarding cross-study reproducibility of the Italian PCA/EFA-based DPs and its sources (Figure 6):

1) population, study design, and dietary assessment tool: observational studies including comparable populations of adults, pregnant women, and the elderly were utilized, with 48% of cross-sectional analyses; reproducible and valid FFQs with a 1- or 2-y reference period were commonly employed [14];
2) DP identification method: similarities were found in the preprocessing of input variables, rotation techniques, and quantitative labeling of DPs, but the lists of input variables varied in terms of number of variables and included variables (nutrients or food groups) [14];
3) DP cross-study reproducibility: the 186 identified DPs representing the Italian diet over the last 30 y were condensed into 11 groups of reproducible DPs targeting the following combinations: pasta/meat, healthy-protein foods/side dish, fruits/vegetables, cheese/deli meats, processed/ready-to-eat foods, animal sources of fats, vegetable sources of fats, and legumes/bread/dairy products [14].

In this paper, we observed the following regarding the consistency of the associations of these groups of reproducible DPs with the same or similar disease outcomes/DP drivers/correlates of interest:

1) statistical methods and adjustment for confounding: regression models were appropriately applied in 98% of the included articles to address the original research questions within the specific study designs; adjustment for confounding was proposed in 96% of articles applying regression models, by including a median number of 7 confounders;
2) consistency of associations: when similar target variables were considered, 9 groups of reproducible DPs showed >50% of statistically significant associations going in the same direction. Exceptions were groups targeting dairies/sweets and vegetable sources of fats. However, 54% of nonsignificant findings were found across combinations of reproducible DPs and similar target variables. Moreover, when the same target variable was considered, the median number of DPs per group was equal to 2 (IQR: 2–2.5). Together with population comparability issues, this prevented us from reliably performing meta-analyses and assessing related heterogeneity and publication bias (Figure 6).

## Discussion

While awaiting the next official Italian food consumption survey—the most recent dating back to 2005–2006—the growing recognition of DPs as key evidence for national dietary guidelines led to the launch of a methodological project focused on PCA/EFA-based DPs in Italy and their relationship with health or disease outcomes [14,85]. A systematic review was conducted to gather available evidence, with a rigorous description of the search process and inclusion/exclusion criteria [25]. To address challenges in defining and interpreting a posteriori DPs [86], statistical and nutritional expertise was applied to define groups of reproducible DPs. The consistency of associations between DPs and similar/the same disease outcomes/drivers/correlates was evaluated across different populations, study designs, and statistical methods, using a selection of Hill’s causal criteria and predefined rules of inference. Although the overall findings that emerged from the application of the majority-rules criterion for consistency of associations were in line with existing literature, insufficient population comparability and a limited number of comparisons hindered the ability to perform meta-analyses on the same available disease outcomes or DP drivers. Regarding the 2 additional criteria of strength of association and dose–response, 2 groups of DPs (***Starchy Patterns*** and ***Vegetable-based*** ***Patterns*** groups) derived from case-control studies showed strong associations with gastric cancer risk in opposite directions, with (***Starchy Patterns****)* or without (***Vegetable-based Patterns***) a linear trend. At this stage, valid scientific conclusions cannot be drawn to inform future updates to Italian nutritional recommendations [25].

When we focused on single DPs and any/similar target variables, we observed that multiple regression models were appropriately applied in most articles, in line with the original research questions and study designs. Several adjustment variables were included, to account for confounding. The selection of specific confounders generally aligned with evidence existing at the time of publication. However, awareness of the importance of specific confounders (for example, physical activity) has increased over recent decades, thus potentially modifying the effect of DPs and reducing residual confounding, when they are inserted in regression models. When we focused on groups of reproducible DPs and the same target variables, stricter control for confounding was applied, including incorporating socioeconomic and anthropometric variables in most analyses. With the exception of cardiovascular and/or cardiometabolic risk factors, effect estimates were generally based on a similar set of confounding variables. The differences observed were consistent with the evidence from the specific populations under consideration.

Using the majority-rules criterion for evaluating consistency of association, we found that most groups of reproducible DPs were associated with one or more of the following similar target variables in the same direction: socioeconomic characteristics, major disease outcomes and related risk factors, overall/cause-specific mortality, pregnancy/breastfeeding-related outcomes, and elderly-related outcomes. Groups of putatively detrimental DPs were associated with lower levels of socioeconomic variables, including food culture, and showed an increased risk of disease, death, or other adverse health outcomes. Conversely, groups of putatively protective DPs showed associations in the opposite direction. The relationships between groups of reproducible DPs and the incidence of chronic diseases, cardiovascular and/or cardiometabolic risk factors, as well as overall/cause-specific mortality, confirm recent findings on the association between suboptimal diets and incidence and mortality/morbidity of noncommunicable diseases [23,87]. This burden is on the rise [87], and underscores the need for policy actions aimed at improving DPs at population level [88]. The putative relationships between socioeconomic factors/food literacy skills and PCA/EFA-based DPs align with previous literature [[89], [90], [91], [92]] and likely reflect the affordability of healthier foods [93,94]. However, we recognize that all included articles except one [71] relied on education or standard single measures of socioeconomic status, some of which had only modest validity in the Italian population [95]. Specific regression models could better capture the complex interactions involving diet, other lifestyle habits, sociodemographic factors, food literacy skills, and food costs/supply, in relation to disease outcomes, potentially within a mediation analysis framework [96,97].

Although the application of the majority-rules criterion for evaluating consistency of association produced findings in line with existing literature, cross-sectional analyses provided all evidence on socioeconomic characteristics and on cardiovascular and/or cardiometabolic risk factors and most evidence from pregnancy/breastfeeding-related and elderly-related outcomes. In addition, when considering the same target variables, we were unable to reliably conduct any additional meta-analyses, to quantify the strength of the consistent associations in 1 pooled measure, assess heterogeneity of studies, and discuss publication bias. Reasons are detailed in the following. With a small number of comparisons (from 2 to 5 comparisons), between-study heterogeneity can be inaccurately estimated, leading to biased pooled effect estimates and overly narrow confidence intervals [98]. Although various meta-analytic approaches to balance empirical coverage and statistical power are being recently developed [98], these fall outside the scope of this project. In addition, heterogeneity of the involved populations (described in Table 2) would likely result in an I^2^ statistic that underestimates the true heterogeneity by a nontrivial margin [32].

Finally, although the direction of the associations is reassuring, 54% of associations between groups of DPs and similar target variables were found to be nonsignificant. In the absence of a formal sample size calculation in most included articles [14], this proportion could be attributed to small-to-moderate sample sizes, which were insufficient to yield precise parameter estimates. When we applied recent guidelines on study power [99] to our results, we found that any regression model including PCA/EFA-based DPs and confounding factors simultaneously would require ≥275 events in median (4 DPs and 7 confounders in median per article, all continuous, would lead to 11 × 25 = 275 needed events), to be further increased because variables were usually expressed in 2 or 3-level categories. On the other hand, the likelihood of finding ≥1 significant association should be higher for the multiple PCA/EFA-based DPs compared with single a priori DPs. Therefore, although the identified proportions of nonsignificant findings could potentially be higher when accounting for the impact of positive-results bias [100,101], the effect of the positive-results bias might be less pronounced with a posteriori DPs. Moreover, the proportion of nonsignificant findings partially reflects a true lack of association for specific groups of reproducible DPs and the same disease outcome/DP driver/correlate. Unfortunately, in this review, we were unable to disentangle the relative contributions of study design, identified DPs (whether well-identified or not, nutrient-based or food-based ones, detrimental or favorable ones), and specific diseases/drivers/correlates considered to the overall likelihood of producing nonsignificant findings in the studies.

In addition to the points discussed in the companion article [14], this analysis presents additional strengths and limitations. Among the major strengths, at article level, DPs can help preempt potential confounding phenomena from other aspects of the diet [4]. At aggregate level, our systematic review provides a methodological framework to investigate on associations involving reproducible DPs and their consistency, strength, and dose–response effects. However, there are notable limitations as well. At article level, several issues could make it difficult to draw valid inferences on DPs [102]. Measurement error was not assessed in any of the included studies [2,4] and the management of missing values, including those on confounders, was not described. Food-based DPs—mostly derived in the selected articles—typically capture only a fraction of the variation in food intake. Potential interactions between DPs and confounders were mostly not formally investigated, as well as additional nonlinearities/nonadditivities between DPs, confounders, and drivers/disease outcomes. Residual confounding can, therefore, remain even when confounders are appropriately included in regression models. Nondifferential measurement error and inappropriate modeling can also exacerbate problems with multicollinearity and residual confounding. At the aggregate level, our systematic review excluded meal patterns identified through PCA/EFA, which could better capture the complexity of dietary behavior compared with standard a posteriori DPs. This includes factors such as meal timing, food combinations within meals, meal distribution throughout the day, and external elements influencing meals [103]. In addition, our synthesis relied on the arbitrary and simplistic criterion of statistical significance [[104], [105], [106]] to manage the vast number of combinations of investigated DPs and disease outcomes/DP drivers/correlates. Finally, we could not account for multiple comparisons involving different disease outcomes/DP drivers from (likely) overlapping populations in the same study (for example, the Moli-sani study) or across the selected articles.

Unlike Castellò et al. [24], we relaxed the criteria for DP reproducibility by considering similarities in original text descriptions and loadings, informed by CCs when available. Had we based our subsequent analysis solely on CCs, we would not have found comparable target variables. This is due to our conservative approach of restricting the CC-based analysis to DPs defined using the same input variables, which meant referencing the same research groups in this systematic review [14]. In future analyses, we plan on assessing reproducibility and consistency of associations of DPs from independent research groups within Italy by utilizing reconstructed DPs based on a newly developed common list of input variables [107]. Finally, it is essential to identify [108] or apply [24] the same DPs in populations from different countries [11]. This may involve cross-confirmatory analyses where both exploratory and confirmatory evaluations are conducted collaboratively across multiple studies [109].

## Author contributions

The authors’ responsibilities were as follows – VE, MF: designed overall research plan and provided supervision on statistical issues; RB, MCS: extracted the data for the systematic review and prepared tables and figures provided in the manuscript; VE: revised all tables and figures, wrote the article, and had primary responsibility for final content; MP: provided supervision on nutritional issues; and all authors: provided critical review of the manuscript and reviewed and approved the final version.

## Data availability

This systematic review made use of publicly available data from published studies. Therefore, no original data are available for sharing. Template data collection forms and data extracted from included studies are available on request from the corresponding author.

## Funding

The authors reported no funding received for this study.

## Conflict of interest

VE was supported by the Young Investigator Grant 2020, from Università degli Studi di Milano. VE, MP, MF, and RB were supported by the Italian Ministry of University and Research [grant numbers PRIN 20227YCB5P to VE (PI), MP (co-PI), and RB (project member), MUR Progetto Eccellenza to DISCCO for VE and MF]. Funders had no role in the review.
