# Supplementary material for: Are Reproducible Dietary Patterns Consistently Associated With Disease Outcomes or Their Drivers in Italy? A Systematic Review
Source: Adv Nutr. 2025 Feb 27;16(4):100397. doi: 10.1016/j.advnut.2025.100397 (PMC11979479; doi:10.1016/j.advnut.2025.100397)
Supplement: Multimedia component 1 [file mmc1.pdf]

Manuscript: "Are reproducible dietary patterns consistently associated with disease outcomes or their drivers in Italy? A systematic review"

Rachele Bianco

Online Supplemental Material

Supplemental Methods

Eligibility criteria

Articles were excluded if: 1. they did not provide original data (e.g., reviews, commentaries, editorials, or personal opinions), or they were case reports, in vitro and in vivo animal studies, conference abstracts or posters; 2. either the reference population lived outside Italy, or populations from different countries, including Italy, were available, but it was not possible to separate out the Italian-specific dietary patterns (DPs) of interest; 3. results concerned single nutrients, single food items, or single food groups; 4. the term "dietary pattern" was used to identify dietary attitudes and perceptions (e.g., feelings felt during meal times, sense of anxiety, perception of self-body image) or patterns of meals; 5. DPs were identified using the *a priori* approach, the mixed-type approach, or the *a posteriori* approach but not following principal component analysis or exploratory factor analysis (e.g., cluster analysis, latent class models, or treelet transform); 6. principal component analysis or exploratory factor analysis were applied on dietary behaviors and not on dietary components; and 7. principal component analysis or exploratory factor analysis were applied on many lifestyle variables, including diet, to derive lifestyle risk patterns.
No restrictions were imposed on year of publication, population characteristics, or health status.

Search strategy

The electronic literature search was conducted in parallel by two authors on 21 December 2022 inserting strings based on keywords and controlled vocabulary terms around the fields of *dietary patterns*, *factor analysis*, *principal component analysis*, and *Italy* in MEDLINE/PubMed, Embase, and Cochrane CENTRAL and Reviews. No reference was added to potential disease outcomes, DP drivers, or correlates of interest, as far as PCA/EFA-based DPs were identifiable in Italy.
Each search string included the following terms: "Feeding Behavior" OR "Diet, Western" OR "diet quality" OR "dietary pattern" OR "diet pattern" OR "food pattern" OR "food intake pattern" OR "food consumption pattern" OR "eating pattern" AND "Factor Analysis, Statistical" OR "Principal Component Analysis" OR "factor" OR "component" OR "score" OR "cluster" AND "Italy" or "Italian", as both keywords and MeSH/Emtree terms (14). Details on the single strings used were provided below.

PubMed	("Feeding Behavior"[Mesh] OR "Diet, Western"[Mesh] OR "diet qualit*" OR "dietary pattern*" OR "diet pattern*" OR "food pattern*" OR "food intake pattern*" OR "food consumption pattern" OR "eating pattern*") AND ("Factor Analysis, Statistical"[Mesh] OR "Principal Component Analysis"[Mesh] OR factor* OR component* OR score* OR cluster*) AND (Italy OR Italian)
Embase	('dietary quality'/exp OR 'dietary pattern'/exp OR 'dietary pattern*' OR 'diet pattern*' OR 'food pattern*' OR 'food intake pattern*' OR 'food consumption pattern*' OR 'eating pattern*' OR 'diet qualit*') AND ('factor analysis'/exp OR 'component analysis'/exp OR 'factor analysis*' OR 'component analysis*' OR factor* OR component* OR cluster* OR score*) AND ('Italy'/exp OR 'italian'/exp OR Italy OR italian)
Cochrane	("Feeding Behavior" OR "Diet, Western" OR “dietary pattern*” OR “diet pattern*” OR “food pattern*” OR “food intake pattern*” OR “food consumption pattern” OR “eating pattern*” OR "diet qualit*") AND ("Factor Analysis, Statistical" OR "Principal Component Analysis" OR factor* OR component* OR score* OR cluster*) AND (Italy OR italian)

After duplicates were removed, two authors independently screened titles and abstracts, and retrieved, screened, and included in the systematic review the relevant articles. The reference lists of the identified articles were also scanned by hand search. Discrepancies were resolved by involving a third researcher (14).

Article-level data extraction

Data extraction was performed independently by two investigators on a predefined Excel spreadsheet and checked by other two investigators; a third one was involved in resolving any potential disagreement. Information extracted from each article included the following: 1. general characteristics of the included studies; 2. study design and characteristics; 3. dietary assessment tool administered; 4. DP identification method; 5. number of DPs extracted, proportion of total variance explained, as well as name and composition of each extracted DP; 6. disease outcome, DP driver, or correlate of interest investigated; 7. statistical methods used to relate the identified DPs to disease outcomes/DP drivers/correlates of interest, and 8. main results on the relationship between identified DPs and disease outcomes/DP drivers/correlates (corresponding to those statistical models adjusted for all the available confounders, if models were fitted) (14).

Article-level quality assessment

Study quality was independently evaluated by two investigators based on the Quality Assessment Tools from the National Institutes of Health National Heart, Lung, and Blood Institute (27); any disagreements were solved by involving a third investigator. To better identify mid/high-quality studies, we added a fourth category, “very good”, to the originally suggested “poor”, “fair”, and “good” (27), in such a way that at least 25% (corresponding to 3 points) of item’s positive answers were included in each category (14).

Supplemental Results

Dietary pattern description and cross-study reproducibility

A total of 186 (food-based DPs: 102; nutrient-based DPs: 84) DPs were identified across all articles included in the systematic review. In the companion article (14), these DPs were collapsed into 113 (food-based DPs: 69; nutrient-based DPs: 44) distinct DPs, providing a 39.3% reduction of overall dietary information (**Supplemental Figure 5**). Below is a summary of the food-based and nutrient-based DPs, further organized into four macro-areas.

Food-based dietary patterns

Within the *Mediterranean-style* macro-area:

1. the ***Mixed-Salad*** group (green) comprised DPs focused on olive oil and a variety of raw (and occasionally cooked) vegetables; this group also featured soup and turkey, or fruits and potatoes as additional food groups in better characterized DPs;
2. the ***Healthy-Protein Foods and Side Dish*** group (blue) comprised DPs focused on at least one source of healthy proteins (i.e., fish, poultry, nuts, and/or legumes) paired with a side dish (e.g., cooked vegetables, potatoes and/or grains, or a combination of these); additional food groups (e.g., fruits) were identified in better characterized DPs;
3. the ***Traditional*** group (brown) comprised DPs focused on legumes, often complemented by semolina-type bread, dairy products, a variety of other vegetables, or eggs and wine.

Within the *Western-style* macro-area:

1. the ***Pasta-and-Meat-oriented*** group (orange) comprised DPs loading high on grains, (red) meat, and animal fats; better characterized DPs within this group also featured additional food groups such as cooked tomatoes, white bread, and wine;
2. the ***Dairy Products and Sweets*** group (yellow) comprised DPs loading high on sweets, dairy products or spreads, and eggs, either individually or in combination;
3. the ***Unhealthy Foods and Snacks*** group (red) comprised DPs loading high on processed foods, like snacks or salty snacks, dipping sauces, deli meats, desserts or sugary/soft drinks, and ready-to-eat dishes; this group also encompassed alcoholic beverages in some DPs.

Nutrient-based dietary patterns

Within the *Animal-oriented* macro-area:

1. the ***Animal-based Patterns*** group (yellow) comprised DPs mostly loading high on animal protein, calcium, cholesterol, saturated fatty acids, riboflavin, phosphorus, and zinc; some DPs also featured animal fat as an additional nutrient;

2. the ***Animal-source Fatty Acids*** group (gray) comprised DPs loading high on vitamin D and other PUFAs, sometimes detailed further into eicosapentaenoic acid, docosahexaenoic acid, and/or docosapentaenoic acid; niacin was also identified as an additional nutrient in three DPs.

Within the *Vegetable-oriented* macro-area:

1. the ***Vegetable-based Patterns*** group (green) comprised DPs loading high on vitamin C, total fiber, and β-carotene equivalents; more characterized DPs featured additional nutrients including total folate, potassium, vitamin B6, vitamin E, soluble carbohydrates, MUFAs, iron, nitrates, lignans, vitamin A, flavonoids, and starch, either individually or in combination;
2. the ***Vegetable-source Fatty Acids*** group (lilac) comprised DPs loading high on linoleic acid, alpha-linolenic acid, and vitamin E; some DPs also featured vegetable fat or the animal sources of fatty acids as additional nutrients;
3. the ***Starchy Patterns*** group (orange) comprised DPs loading high on starch, vegetable protein, and sodium; some DPs also featured given PUFAs or other PUFAs as additional nutrients.

Finally, we matched the following groups of nutrient-based and food-based DPs:

1. ***Mixed-Salad*** and ***Vegetable-based Patterns***;
2. ***Pasta-and-Meat-oriented*** and ***Starchy Patterns***;
3. ***Dairy Products and Sweets*** and ***Animal-based Patterns****,*

by inspecting correlation coefficients between nutrient-based DPs and selected food groups provided in the original articles (**Supplemental Figure 5**, solid line, and (14) for details).
When additionally merging DPs with the same color code in this article, the 113 DPs summarized in the companion article (14) were consolidated into 76 DPs, resulting in an additional 33% reduction. These DPs were related to disease outcomes/DP drivers/correlates of interest, when available, in this article.

Dietary patterns in relation to drivers/correlates of interest: main results

The relationships between the identified DPs and their drivers/correlates went in the expected direction across available target populations of children/adolescents (70, 71), adults (48-51, 64, 66, 72), and the elderly (55). Putatively detrimental DPs were related to lower socio-economic characteristics, including lower nutritional knowledge/culture, whereas putatively protective DPs acted in the opposite direction. Details for the single drivers and corresponding DPs follow. First, putatively detrimental DPs were generally related to lower education, income, or socio-economic status, whereas putatively protective DPs to higher categories. In detail, the *Eggs and Sweets* DP (***Dairy Products and Sweets*** group) was related to a lower income (48). The *Western*, *Beverages and Convenience* (both from the ***Unhealthy Foods and Snacks*** group), and the *Pasta-and-Meat* (***Pasta-and-Meat-oriented*** group) DPs were related to a lower education (55, 64, 72). On the other hand, the *Olive Oil and Vegetables* DP (***Mixed-Salad*** group) was related to a higher income (48). Similarly, a higher education was related to higher scores in the *Wide Range* or *Prudent* DPs (both from ***Healthy-Protein Foods and Side Dish*** group) (55, 72), but not significantly associated with the *Olive Oil and Salad* DP (***Mixed-Salad*** group) (55) or the *Prudent* DP (***Healthy-Protein Foods and Side Dish*** group) (64) (**Figures 2a** and **2b**).

In addition, in the Moli-sani study, putatively detrimental DPs were related to a lower mass media exposure and food culture, whereas putatively protective DPs acted in the opposite direction (49-51). In detail, mean factor scores of the three *Olive Oil and Vegetables* (***Mixed-Salad*** group) DPs (49-51) were higher in adults who were heavily exposed to mass media, showed the highest level of nutritional knowledge, or read food labels. In two of these articles (49, 51), mean factor scores of the *Pasta and Meat* (***Pasta-and-Meat-oriented*** group) DPs were lower in heavy mass media exposure (49) and food label readers (51) groups, whereas mean factor scores of the *Eggs and Sweets* (***Dairy Products and Sweets*** group) were additionally lower in food label readers only (51) (**Figure 2a**).

These results were in line with those on socio-economic status for children (71) and on school performance for adolescents (70). In children, increased mean factor scores in the *Processed* DP (***Unhealthy Foods and Snacks*** group) were associated with a reduced family socio-economic status indicator, but no material association emerged for the *Healthy* (***Healthy-Protein Foods and Side Dish*** group) and *Spreads* (***Dairy Products and Sweets*** group) DPs (71). In adolescents, higher scores in the *Western* and *Energy-dense* DPs (both from the ***Unhealthy Foods and Snacks*** group) were inversely correlated with school marks (English, History, Science, Physical Education, the overall Grade Point Average and mark for conduct for the *Western* DP; Italian only for the *Energy-dense* DP); on the opposite side, higher scores in the *Prudent* (***Healthy-Protein Foods and Side Dish*** group) DP were positively correlated with higher marks in Mathematics (**Figure 2b**).

In conclusion, although previous articles (48-51, 55, 64, 70-72) did not cover food-based DPs from the ***Traditional Patterns*** group, they provided significant associations in 60% (73/121) of the possible combinations of investigated DPs and drivers/correlates, which increased to 67% (65/97) when we restricted the analysis to DP drivers only.

Dietary patterns in relation to disease outcomes of interest: main results

Incidence of chronic diseases in the adult population

The most investigated outcome across articles was cancer incidence (or related risk factors) in the adult population (33-44, 59-61, 68, 69, 82) (**Figures 3a** and **4a**). Among food-based DPs, the *Salad Vegetables* (***Mixed-Salad*** group) showed a protective effect on breast cancer risk overall (60) and among HER2+ cancer patients (61) in the “Ormoni e Dieta nell'Eziologia del Tumore della Mammella (ORDET)” cohort from the north of Italy, although not confirmed either in the re-analysis presented in (59) or among HER2- cancer patients (61); in addition, their *Prudent* DP (***Healthy-Protein Foods and Side Dish*** group) was not associated with risk of breast cancer overall and in the two subtypes of HER2+/HER2- (60, 61). Similarly, no significant association was found between high-grade cervical intraepithelial neoplasia and another *Prudent* DP (***Healthy-Protein Foods and Side Dish*** group) identified in the south of Italy. On the other hand, neither the *Canteen*/*Western*/*Pork, Processed Meat, Potatoes* DPs (all from the ***Pasta-and-Meat-oriented*** group) from the north of Italy (59-61) nor the *Western* DP (***Unhealthy Foods and Snacks*** group) from the south of Italy (68) were materially related to breast or cervical cancer risk. No food-based DPs from the ***Dairy Products and Sweets*** or the ***Traditional Patterns*** groups were found in previous articles (59-61, 68, 69) and later related to cancer incidence (**Figure 3a**).

Among the nutrient-based DPs, within the ***Animal-based Patterns*** group, the *Animal Products* DP was a risk factor for most cancer sites (34, 35, 37-42), with the exception of a protective effect for breast cancer (33); a non-significant effect was found for other sites (33, 36, 43, 44) and for the *Refined* DP in gastric cancer (82). Similarly, within the ***Starchy Patterns*** group, the *Starch-rich* DP was a risk factor for breast and ovarian (33), gastric (34), colorectal (36), pancreatic (39), prostatic (40), nasopharyngeal (42), and renal-cell (43) cancers, although it was not related to (37, 38, 41, 44) – or protective for (35) – cancer at other sites; the *Traditional* DP from Tuscany was still a risk factor for gastric cancer (82). On the other hand, within the ***Vegetable-based Patterns*** group, the *Vitamins and Fiber* DP showed a general protective effect on several cancer sites (33-39, 44) – confirmed for the *Vitamin-rich* DP from Tuscany in gastric cancer (82) – versus some non-significant findings (33, 36, 40-43). Within the ***Vegetable-source Fatty Acids*** group, the *Vegetable Unsaturated Fatty Acids - VUFA* (or *Unsaturated Fats*, or *Cooking Oils and Dressings*) DPs were protective against cancer at four sites (33, 35, 36, 43), but at risk for nasopharynx (42) or not associated with cancer at other sites (33, 34, 36-41, 82). Within the ***Animal-source Fatty Acids*** group, the *Animal Unsaturated Fatty Acids - AUFA* (or *Other PUFAs and Vitamin D*) DPs were at risk for cancer at four sites (37, 40-42), protective at two additional sites (i.e., colon and esophagus (36, 38)), or not associated with another three sites (i.e., oral cavity, rectum and bladder (35, 36, 44)) (**Figure 4a**).
Incidence of other chronic diseases was investigated in the general adult population for coronary heart disease (CHD) at 20 years, with *Factor2* (***Healthy-Protein Foods and Side Dish*** group) being a protective DP versus *Factor1* and *Factor3* (both from the ***Dairy Products and Sweets*** group) being unrelated to the outcome (62). Incidence of type 2 diabetes was not significantly associated with *PC1* (***Mixed-Salad*** group) or *PC2 (**Pasta-and Meat-oriented*** group*)* (57). No food-based DPs from the ***Unhealthy Foods and Snacks*** or the ***Traditional*** groups were found in previous articles (57, 62) and later related to incidence of CHD or type 2 diabetes (**Figure 3a**).

Overall and cause-specific mortality in the adult population

Overall and/or cause-specific mortality was investigated in five articles (53, 56, 62, 63, 75). While overall mortality was not related to any DPs (from the ***Mixed-Salad***, ***Pasta-and-Meat-oriented***, and ***Dairy Products and Sweets*** groups) in subjects with type 2 diabetes in the Moli-sani study (53), in the Seven Countries study, *Factor2/FA2/PC2* (***Healthy-Protein Foods and Side Dish*** group) exerted a protective effect on overall, cancer-specific, cardiovascular disease (CVD)-specific, and CHD-specific mortality at 40 years (62), the last confirmed in a more recently published article (63); in addition, higher scores on *Factor3* (***Dairy Products and Sweets*** group) decreased overall mortality risk, although *Factor1* from the same group was not significantly associated with overall or cause-specific mortality outcomes (62). The *Olive Oil and Salad* DP (***Mixed-Salad*** group) was inversely related to risk of overall mortality in the "European Prospective Investigation into Cancer and Nutrition (EPIC)-Elderly" study, where, however, non-significant findings were found for the remaining DPs (***Pasta-and-Meat-oriented***, ***Healthy-Protein Foods and Side Dish***, and ***Dairy Products and Sweets*** groups) (56). Finally, the *Farm-house Diet* (***Traditional Patterns*** group) but not the *Elderly* Pattern from the same group increased overall mortality risk in the "Salus in Apulia" study; in addition, no significant relations were found with the other identified DPs belonging to the ***Healthy-Protein Foods and Side Dish***, the ***Dairy Products and Sweets***, and the ***Unhealthy Foods and Snacks*** groups (75) (**Figure 3a**).

Cardiovascular and/or cardiometabolic risk factors in adults and children

The relationship between identified DPs and cardiovascular and/or cardiometabolic risk factors was investigated in adults (47, 74) and children (77). In adults, DPs belonging to the ***Mixed-Salad*** and ***Healthy-Protein Foods and Side Dish*** groups (*Olive Oil and Vegetables* (47) and *Mediterranean (PC2)* (74), respectively) were consistently related to lower blood glucose, blood pressure, and inflammatory markers, including C-reactive protein (47) and leucocytes (74). On the other hand, DPs from the ***Pasta-and-Meat-oriented*** and the ***Unhealthy Foods and Snacks*** (*Pasta-and-Meat* (47) and *Western (PC1)* (74), respectively) were consistently related to higher blood glucose, total and LDL cholesterol, and respective markers of inflammation. In addition, within the same ***Dairy Products and Sweets*** group, the *Residual (PC4)* (74) and the *Eggs and Sweets* (47) DPs were related to previous markers of inflammation, but not to systolic and diastolic blood pressure, total and LDL cholesterol, and blood glucose. Finally, the mean arterial stiffness was higher for the *Western (PC1)* DP (***Unhealthy Foods and Snacks*** group) (74); the CUORE-CVD-risk score was higher for the *Pasta-and-Meat* DP (***Pasta-and-Meat-oriented*** group) and, in males only, for the *Eggs and Sweets* DP (***Dairy Products and Sweets*** group) (47). Among children, higher scores on the *Healthy* DP (***Healthy-Protein Foods and Side Dish*** group) were related to a lower mean capillary glucose, in comparison with a lack of statistically significant association for the corresponsing *Unhealthy* DP (***Unhealthy Foods and Snacks*** group) (77). No food-based DPs from the ***Traditional Patterns*** group were found in previous articles (47, 74, 77) and later related to cardiovascular and/or cardiometabolic risk factors (**Figure 3b**).

Elderly-related outcomes

Within the elderly population (55, 56, 76, 80, 81), three food-based DPs from the ***Pasta-and-Meat-oriented***, ***Healthy-Protein Foods and Side Dish***, and ***Traditional Patterns*** groups *(Pattern 1, Pattern 2*, and *Pattern 5*, respectively*)* favored a higher mean bone mineral density and/or a reduced odds ratio of fractures in Calabria (80). Within the same study, cognitive performance improved at follow-up for high consumers of the food-based *Legumes* DP (***Traditional Patterns*** group) and the nutrient-based *Plant Proteins and Polyunsaturated Fats Pattern* (***Starchy Patterns*** group) (81) (**Figure 3c**).

Pregnancy/breastfeeding-related outcomes

Concerning pregnancy/breastfeeding outcomes (64-66, 73, 83, 84), a lower mean pre-gestational BMI (65) was associated with higher adherence to a *Prudent* DP (***Healthy-Protein Foods and Side Dis*h** group) during pregnancy, whereas a high adherence to the *High Meat, Animal Fats, Grains* DP (***Pasta-and-Meat-oriented*** group) was cross-sectionally related to worse mean maternal biomarkers during pregnancy (83) (**Figure 3b**). In addition, mother's healthier DPs ended up into a better foremilk composition: higher scores on DPs from the ***Vegetable-based Patterns*** and ***Animal-source Fatty Acids*** groups (*Vitamins, minerals, and fibers* and *Fatty Acids with Fins*, respectively) were associated with higher mean omega-3, eicosapentaenoic acid, docosahexaenoic acid, and/or docosapentaenoic acid in mother's milk, whereas higher scores on the *Fatty Acids with Leaves* DP from the ***Vegetable-source Fatty***

Acids group provided higher mean MUFAs and alpha-linolenic acid, as well as lower saturated fatty acids in milk (73) (**Figure 4b**).

Childhood-related outcomes

Within the children/adolescents population (46, 70, 71, 77), three nutrient-based DPs in 7-year-old children from Friuli Venezia Giulia (46) were related to single components of overall cognitive performance: the *Meat and Potatoes* and *Dairy Products* DPs (both from the ***Animal-based Patterns*** group) were related to mean decreased verbal comprehension and processing speed, respectively; the *Seafood* DP (from the ***Animal-source Fatty Acids*** group) was related to increased verbal comprehension and perceptual reasoning (**Figure 4b**).
In conclusion, the previous articles covered all groups of nutrient-based and food-based DPs and provided significant associations in 42% (139/334) of the possible combinations of identified DPs and selected outcomes. Across investigated outcomes, we observed that:

1. nutrient-based DPs were significantly related to incidence of chronic diseases in 59% (40/68, from cancer incidence only) (**Figure 4a**) versus 14% (3/21, i.e., 2/16: cancer incidence; 1/3: CHD incidence; 0/2: type-2 diabetes incidence) (**Figure 3a**) of the combinations concerning food-based DPs, giving a total of 48% (43/89) of the combinations;
2. food-based DPs were significantly related to overall/cause-specific mortality in 36% (9/25) of the combinations (overall mortality: 26.7%=4/15) (**Figure 3a**);
3. food-based DPs were significantly related to cardiovascular and/or cardiometabolic risk factors in adults and children in 51% (39/77) of the combinations (**Figure 3b**);
4. nutrient-based DPs were significantly related to pregnancy/breastfeeding outcomes in 40% (24/60) of the combinations (**Figure 4b**) versus 36% (4/11) of the combinations concerning food-based DPs (**Figure 3b**), giving a total of 39% (28/71) of the combinations.

Supplemental Table 1. Main characteristics of studies identifying dietary patterns using principal component and factor analyses in Italy

Reference, location, study name, study quality	Study design	Participants	Dietary questionnaire	Outcome/Dietary pattern driver/Correlate
Edefonti, 2008 (33)<br>Breast cancer: northern Italy (Milan, Genoa, Gorizia, Forli), central and southern Italy (Latina, Naples)<br>Ovarian cancer: northern Italy (Milan, Pordenone, Padua), central and southern Italy (Latina, Naples)<br>Good quality	Case-control study; 2 companion studies on breast and ovarian cancers; hospital based; recruitment from 1991 to 1994 for the breast cancer study and from 1992 to 1999 for the ovarian cancer study; Italian multicentric	7013 total subjects (100% Fs); 2569 breast cancer cases 25-74 ys (median: 55 ys, NA); 1031 ovarian cancers cases 18-79 ys (median: 56 ys, NA); 3413 controls 17-79 ys (median: 57 ys, NA)	FFQ<br>2 ys before<br>IA<br>Reproducible and valid<br>78 FIs (30 NUTs)	Breast and ovarian cancer incidence
Bertuccio, 2009 (34)<br>Milan (Lombardy)<br>Good quality	Case-control study; gastric cancer; hospital based; recruitment from 1997 to 2007; single center/area	777 total subjects; 230 cases (143 Ms, 87 Fs) 22-80 ys (median: 63 ys, NA); 547 controls (286 Ms, 261 Fs) 22-80 ys (median: 63 ys, NA)	FFQ<br>2 ys before<br>IA<br>reproducible and valid<br>78 FIs (28 NUTs)	Gastric cancer incidence
Edefonti, 2010 (35)<br>Milan (Lombardy), Pordenone (Friuli Venezia Giulia), Rome, Latina (Lazio)<br>Good quality	Case-control study; oral cavity cancer; hospital based; recruitment from 1992 to 2005; Italian multicentric	2886 total subjects; 805 cases (659 Ms, 146 Fs) 22-78 ys (median: 58 ys, NA); 2081 controls (1302 Ms, 779 Fs); 19-79 ys (median: 58 ys, NA)	FFQ<br>2 ys before<br>IA<br>Reproducible and valid<br>78 FIs (29 NUTs)	Oral cavity and pharyngeal cancer incidence

Bravi, 2010 (36)<br>Milan (Lombardy), Genoa (Liguria), Pordenone, Gorizia (Friuli Venezia Giulia), Forlì (Emilia-Romagna), Latina (Lazio), Naples (Campania)<br>Good quality	Case-control study; colorectal cancer; hospital based; recruitment from 1992 to 1996; Italian multicentric	6107 total subjects; 1225 colon cancer cases (688 Ms, 537 Fs) 19-74 ys (median: 62 ys, NA); 728 rectum cancer cases (437 Ms, 291 Fs) 23-74 ys (median: 62 ys, NA); 4154 controls (2073 Ms, 2081 Fs) 19-74 ys (median: 58 ys, NA)	FFQ<br>2 ys before<br>IA<br>Reproducible and valid<br>78 FIs (28 NUTs)	Colorectal cancer incidence
Edefonti, 2010 (37)<br>Milan (Lombardy), Pordenone (Friuli Venezia Giulia)<br>Good quality	Case-control study; laryngeal cancer; hospital based; recruitment from 1992 to 2000; Italian multicentric	1548 total subjects; 460 cases (415 Ms, 45 Fs) 30-80 ys (median: 61 ys, NA); 1088 controls (863 Ms, 225 Fs) 31-79 ys (median: 61 ys, NA)	FFQ<br>2 ys before<br>IA<br>Reproducible and valid<br>78 FIs (28 NUTs)	Squamous cell laryngeal cancer incidence
Bravi, 2012 (38)<br>Milan (Lombardy), Pordenone (Friuli Venezia Giulia), Padua (Veneto)<br>Good quality	Case-control study; esophageal cancer; hospital based; recruitment from 1992 to 1997; Italian multicentric	1047 total subjects; 304 cases (275 Ms, 29 Fs) 39-77 ys (median: 60 ys, NA); 743 controls (593 Ms, 150 Fs) 33-77 ys (median: 60 ys, NA)	FFQ<br>2 ys before<br>IA<br>Reproducible and valid<br>78 FIs (28 NUTs)	Esophageal cancer incidence
Bosetti, 2013 (39)<br>Milan (Lombardy), Pordenone (Friuli Venezia Giulia)<br>Good quality	Case-control study; pancreatic cancer; hospital based; recruitment from 1991 to 2008; Italian multicentric	978 total subjects; 326 cases (174 Ms, 152 Fs) (median: 63 ys, NA); 652 controls (348 Ms, 304 Fs) (median: 62 ys, NA)	FFQ<br>2 ys before<br>IA<br>Reproducible and valid<br>78 FIs (28 NUTs)	Pancreatic cancer incidence
Rosato, 2014 (40)<br>Milan (Lombardy), Pordenone, Gorizia (Friuli Venezia Giulia) Latina (Lazio), Naples (Campania)<br>Good quality	Case-control study; prostate cancer; hospital based; recruitment from 1991 to 2002; Italian multicentric	2745 total subjects (100% Ms); 1294 cases 46-74 ys (median: 66 ys, NA); 1451 controls 46-74 ys (median: 63 ys, NA)	FFQ<br>2 ys before<br>IA<br>Reproducible and valid<br>78 FIs (28 NUTs)	Prostatic cancer incidence

Bravi, 2015 (41)<br>Milan (Lombardy), Pordenone, Udine (Friuli Venezia Giulia), Naples (Campania)<br>Good quality	Case-control study; endometrial cancer; hospital based; recruitment from 1992 to 2006; Italian multicentric	1362 total subjects (100% Fs); 454 cases 18-79 ys (median: 60 ys, NA); 908 controls 19-80 ys (median: 61 ys, NA)	FFQ<br>2 ys before<br>IA<br>Reproducible and valid<br>78 FIs (28 NUTs)	Endometrial cancer incidence
Edefonti, 2015 (42)<br>Milan (Lombardy), Pordenone (Friuli Venezia Giulia), Naples (Campania), Catania (Sicily)<br>Good quality	Case-control study; nasopharyngeal cancer; hospital based; recruitment from 1992 to 2008; Italian multicentric	792 total subjects; 198 cases (157 Ms, 41 Fs) 18-76 ys (median: 52 ys, NA); 594 controls (471 Ms, 123 Fs) 19-76 ys (median: 52 ys, NA)	FFQ<br>2 ys before<br>IA<br>Reproducible and valid<br>78 FIs (28 NUTs)	Nasopharyngeal carcer incidence
Dalmartello, 2020 (43)<br>Milan (Lombardy), Pordenone, Udine (Friuli Venezia Giulia), Latina (Lazio), Naples (Campania)<br>Good quality	Case-control study; renal cell cancer; hospital based; recruitment from 1992 to 2004; Italian multicentric	2301 total subjects; 767 cases (494 Ms, 273 Fs) 24-79 ys (median: 62 ys, NA); 1534 controls (988 Ms, 546 Fs) 22-79 ys (median: 62 ys, NA)	FFQ<br>2 ys before<br>IA<br>Reproducible and valid<br>78 FIs (28 NUTs)	Renal cell cancer incidence
Edefonti, 2020 (44)<br>Milan (Lombardy), Pordenone (Friuli Venezia Giulia), Naples (Campania), Catania (Sicily)<br>Good quality	Case-control study; bladder cancer; hospital based; recruitment from 2003 to 2014; Italian multicentric	1355 total subjects; 690 cases (595 Ms, 95 Fs) 25-84 ys (median: 67 ys, NA); 665 controls (561 Ms, 104 Fs) 27-84 ys (median: 66 ys, NA)	FFQ<br>2 ys before<br>IA<br>Reproducible and valid<br>80 FIs (28 NUTs)	Urothelial bladder cancer incidence
Edefonti, 2020 (45)<br>Milan (Lombardy)<br>Good quality	Cross-sectional study; rheumatoid arthritis disease activity; recruitment from January 2018 to December 2019; single center/area with recruitment at Pini Hospital (Milan)	205 total subjects (40 Ms, 165 Fs) 18-65 ys (median: 58.46 ys, IQR: 47.81-69.03 ys)	FFQ<br>6 mos before<br>IA<br>Reproducible and valid<br>110 FIs (33 NUTs)	Rheumatoid arthritis disease activity, measured with DAS28-CRP and SDAI

Marinoni, 2022 (46)<br>Croatia, Greece, Italy (Friuli Venezia Giulia region)<br>Good quality	Cross-sectional analysis nested within the NAC-II birth cohort which followed-up 632 eligible (i.e, 18-month children with neurodevelopment assessed) born from 767 mothers originally recruited between 2007 and 2009; international	379 total subjects (195 Ms, 184 Fs); mean: 7 ys, SD: 0.05 ys	3d-DR (2 weekdays and 1 weekend day, not necessarily consecutive) in the wk before<br>IA<br>Reproducible and valid<br>828 FIs (37 NUTs)	Cognitive abilities assessed with the validated Italian version of the WISC-IV and expressed by FSIQ, and other 4 indexes: VCI, PRI, WMI, and PSI
Centritto, 2009 (47)<br>Molise<br>Moli-sani<br>Good quality	Cross-sectional study; men and women living in Molise randomly recruited from city-hall registries of Molise by using electronically generated numbers; 16704 subjects recruited from 2005 to 2008; single center/area	7646 total subjects (49% Ms, 51% Fs); age ≥ 35 ys (mean: 50 ys, SE: 10 ys)	Modified version of the reproducible and valid EPIC FFQ to include some typical southern Italy foods<br>NA reference period<br>SA<br>Validated in a different form<br>188 FIs (45 FGs)	CVD risk profile according to a series of clinical parameters: total, HDL, and LDL cholesterol, SBP, DBP, TGs, blood glucose, CRP, and a global individual CVD risk score calculated applying the risk equation from the CUORE project
Bonaccio, 2012 (48)<br>Molise<br>Moli-sani<br>Good quality	Cross-sectional study; men and women living in Molise randomly recruited from city-hall registries of Molise by using electronically generated numbers; 24325 subjects recruited from March 2005 to April 2010; single center/area	13262 total subjects (6590 Ms, 6672 Fs); age ≥ 35 ys (mean: 53.3 ys, SD: 10.6 ys)	Modified version of the reproducible and valid EPIC FFQ to include some typical southern Italy foods<br>NA reference period<br>SA<br>Validated in a different form<br>188 FIs (43 FGs)	Household net income in categories (low, low-medium, medium-high, and high)

Bonaccio, 2012 (49)<br>Molise<br>Moli-sani<br>Good quality	Cross-sectional study; men and women living in Molise randomly recruited from city-hall registries of Molise by using electronically generated numbers; 1132 subjects recruited from May 2009 to April 2010; single center/area	959 total subjects (479 Ms, 480 Fs) aged ≥ 35 ys (mean: 52.8 ys, SD: 9.6 ys)	Modified version of the reproducible and valid EPIC FFQ to include some typical southern Italy foods<br>NA reference period<br>SA<br>Validated in a different form<br>188 FIs (45 FGs based on reference to a previous article)	Mass media exposure score obtained using principal component analysis (PCA) on items from an additional validated questionnaire on nutrition knowledge and mass media exposure
Bonaccio, 2013 (50)<br>Molise<br>Moli-sani<br>Good quality	Cross-sectional study; men and women living in Molise randomly recruited from city-hall registries of Molise by using electronically generated numbers; 1132 subjects recruited from May 2009 to April 2010; single center/area	744 total subjects (50.3% Ms, 49.7% Fs); age ≥ 35 ys (mean: 52.1 ys, SD: 9.4 ys)	Modified version of the reproducible and valid EPIC FFQ to include some typical southern Italy foods<br>NA reference period<br>SA<br>Validated in a different form<br>188 FIs (43 FGs)	Nutrition knowledge (categorical) obtained from an additional validated questionnaire on nutrition knowledge and mass media exposure
Bonanni, 2013 (51)<br>Molise<br>Moli-sani<br>Good quality	Cross-sectional study; men and women living in Molise randomly recruited from city-hall registries of Molise by using electronically generated numbers; 1571 subjects recruited from May 2009 to April 2010; single center/area	883 total subjects (442 Ms, 441 Fs); age ≥ 35 ys (mean: 52.5 ys, SD: 9.6 ys)	Modified version of the reproducible and valid EPIC FFQ to include some typical southern Italy foods<br>NA reference period<br>SA<br>Validated in a different form<br>188 FIs (45 FGs based on reference to a previous article)	Food labels reading (categorical) obtained from an additional validated questionnaire on nutrition knowledge and mass media exposure

Bonaccio, 2013 (52)<br>Molise<br>Moli-sani<br>Good quality	Cross-sectional study; men and women living in Molise randomly recruited from city-hall registries of Molise by using electronically generated numbers; 24325 subjects recruited from March 2005 to April 2010; single center/area	16937 total subjects (48.4% Ms, 51.6% Fs); age ≥ 35 ys (mean: 53.0 ys, SD: 10.8 ys)	Modified version of the reproducible and valid EPIC FFQ to include some typical southern Italy foods<br>NA reference period<br>SA<br>Validated in a different form<br>188 FIs (43 FGs)	Health-related quality of life (mental and physical health components) from the validated Italian version of SF-36 questionnaire (from 0 - worst possible condition - to 100 - best possible condition)
Bonaccio, 2016 (53)<br>Molise<br>Moli-sani<br>Good quality	Prospective cohort study; men and women living in Molise randomly recruited from city-hall registries of Molise by using electronically generated numbers; 24325 subjects recruited from March 2005 to April 2010 for a final sample of 1995 patients with type 2 diabetes followed-up for mortality until December 2011; single center/area	1995 total subjects (1319 Ms, 676 Fs); age ≥ 35 ys (mean: 62.6 ys, SD: 10.2 ys)	Modified version of the reproducible and valid EPIC FFQ to include some typical southern Italy foods<br>NA reference period<br>SA<br>Validated in a different form<br>188 FIs (46 FGs)	Overall mortality in diabetic subjects
Bonaccio, 2018 (54)<br>Molise<br>Moli-sani<br>Good quality	Cross-sectional study; men and women living in Molise randomly recruited from city-hall registries of Molise by using electronically generated numbers; 24325 subjects recruited from March 2005 to April 2010; single center/area	11272 total subjects (46.2% Ms, 53.8% Fs) age ≥ 35 ys (mean: 52.7 ys, SD: 10.8 ys) reduced to 10812 due to unreliable medical or dietary questionnaires, implausible EIs or missing values for dietary information	Modified version of the reproducible and valid EPIC FFQ to include some typical southern Italy foods<br>NA reference period<br>SA<br>Validated in a different form<br>188 FIs (46 FGs)	Psychological resilience from 25-item Connor-Davidson Psychological Resilience Scale (from 0 to 100 with a higher score reflecting greater psychological resilience)
Pala, 2006 (55)<br>Denmark, France, Germany, Greece, Netherlands, Spain, Sweden, UK, Italy (Varese, Turin, Florence, Naples, Ragusa)	Cross-sectional analysis nested within a prospective cohort study; elderly (≥60 ys) participants from EPIC study recruited voluntarily from 1993 to 1998 in 5 different areas covered by cancer registries in northern, central and southern Italy; international	100 059 total subjects; 5611 Italian participants: 1536 Ms (60.0-72.2 ys, median age at enrollment: 62.3 ys, IQR: NA), 4075 Fs (60.0-77.8 ys, median age at enrollment: 62.3 ys, IQR: NA)	3 different FFQs<br>1 y before<br>NA<br>Reproducible and valid<br>188 FIs (Varese, Turin, Florence), 217 FIs (Ragusa), 140 FIs (Naples), (57 FGs for all centers)	Lifestyle, anthropometry, education, and other health indicators

EPIC (EPIC-Elderly)<br>Good quality				
Masala, 2007 (56)<br>Denmark, France, Germany, Greece, Netherlands, Spain, Sweden, UK, Italy (Varese, Turin, Florence, Naples, Ragusa)<br>EPIC (EPIC-Elderly)<br>Very good quality	Prospective cohort study; elderly (≥60 ys) participants from EPIC study recruited voluntarily in 5 different areas covered by cancer registries in northern, central and southern Italy between 1993 and 1998 and followed-up for overall mortality up to 2001 or 2002 (median follow-up of 6.2 ys after applying exclusion criteria); international	100 059 total subjects; 5611 Italian participants: 1536 Ms (60.0-72.2 ys, median age at enrollment: 62.3 ys, IQR: NA), 4075 Fs (60.0-77.8 ys, median age at enrollment: 62.3 ys, IQR: NA), 152 total deaths	3 different FFQs<br>1 y before<br>NA<br>Reproducible and valid<br>188 FIs (Varese, Turin, Florence), 217 FIs (Ragusa), 140 FIs (Naples), (57 FGs for all centers)	Overall mortality
Jannasch, 2019 (57)<br>Italy, France, Spain, UK, Netherlands, Germany, Sweden, Denmark<br>EPIC-InterAct<br>Good quality	Case-cohort study nested within EPIC prospective cohort study and based on incident cases of type 2 diabetes in the full EPIC cohort (cases which occurred between 1991 and the 31 December 2007 in 8 countries) and a randomly drawn subcohort stratified by center (9 centers); international	25877 total subjects of which 14694 randomly drawn subcohort subjects and 11183 verified incident type 2 diabetes cases; 719 verified incident type 2 diabetes cases overlapping with the subcohort; 1927 Italian participants in the subcohort (32.3% Ms, 67.7% Fs), mean: 50.2 ys, SD: 7.9 ys at baseline	Reproducible and valid country specific FFQs<br>1 y before<br>NA FIs (36 FGs)	Type 2 diabetes incidence
Balder, 2003 (58)<br>Netherlands, Sweden, Finland, and Italy<br>DIETSCAN Project (NLSC, SMC, ATBC, ORDET)<br>Poor quality	Parallel analysis of 4 prospective cohort studies on diet and cancer according to the same strategy (no pooled analysis); NLSC (random subcohort of): population-based cohort of Ms and Fs from Dutch municipalities that began in 1986; SMC: population-based cohort of Fs based on a mammography screening in 2 countries in central Sweden from 1987 to 1990; ATBC: randomized placebo-controlled intervention study conducted among M smokers who	100911 total subjects; ORDET: 9208 Fs with complete dietary data (mean age at baseline: 48.6 ys, SE: 8.6 ys, 35–69 ys); median follow-up and number of deaths not reported	4 different but validated FFQs;<br>ORDET-FFQ: 1 y before;<br>SA;<br>Reproducible and valid;<br>107 FIs (51 FGs, but final number equal to 32, due to ORDET availability)	No outcomes, DP drivers, or correlates of interest;<br>Evaluation of cross-study reproducibility of identified DPs

	lived in south-western Finland (1985–1988); ORDET: cohort study of Italian healthy volunteer Fs from the province of Varese, northern Italy (1987–1992); international			
Männistö, 2005 (59)<br>Netherlands, Sweden, and Italy<br>DIETSCAN Project (NLSC, SMC, ATBC, ORDET)<br>Good quality	Parallel analysis of 3 prospective cohort studies on diet and cancer according to the same strategy (no pooled analysis); NLSC (random subcohort of): population-based cohort of Ms and Fs from Dutch municipalities that began in 1986; SMC: population-based cohort of Fs based on a mammography screening in 2 countries in central Sweden from 1987 to 1990; all invasive breast cancer cases were identified through national or local cancer registers; ORDET: cohort study of Italian healthy volunteer Fs from the province of Varese, northern Italy (enrollment from 1987 to 1992; 9 ys follow-up); international; re-analysis of DPs originally derived in Balder et al. 2003	73849 total subjects (3271 breast cancer cases with complete information on their diet); ORDET: 10788 Fs (mean age at baseline: 48 ys; SE: 8.5 ys, 35-69 ys), 212 breast cancer cases	3 different but validated FFQs: ORDET-FFQ: 1 y before; SA; Reproducible and valid; 107 FIs (51 FGs, but final number equal to 32)	Breast cancer incidence
Sieri, 2004 (60)<br>Varese (Lombardy)<br>ORDET<br>Very good quality	Prospective cohort study; Italian healthy volunteer women from the province of Varese, northern Italy; cancer cases identified through local cancer registry; recruitment from 1987 to 1992; 9.5 ys of average follow-up; single center/area	8984 subjects 100% Fs (34-70 ys) based on a total of 10786 subjects; 207 incident breast cancer cases	FFQ<br>1 y before<br>SA<br>Reproducible and valid<br>107 FIs (34 FGs)	Breast cancer incidence

Sant, 2007 (61)<br>Varese (Lombardy)<br>ORDET<br>Very good quality	Prospective cohort study; Italian healthy volunteer women from the province of Varese, northern Italy; cancer cases identified through local cancer registry; recruitment from 1987 to 1992; 11.5 ys of average follow-up; single center/area; re-analysis of DPs originally provided in Sieri et al. 2004	8861 subjects 100% Fs (34-70 ys) based on a total of 8984 subjects recruited in a previous ORDET study; 267 incident breast cancer cases by December 31, 2001, with availability of HER2 status in 238 of them	FFQ<br>1 y before<br>SA<br>Reproducible and valid<br>107 FIs (34 FGs)	Breast cancer incidence (HER2+ vs HER2-)
Menotti, 2012 (62)<br>Italian Rural Areas of Seven Countries Study of Cardiovascular Disease<br>Seven Countries Study<br>Very good quality	Prospective cohort study; enrollment in 1960 from the Italian Rural Areas cohorts, follow-up of 20 ys for CHD events and 40 ys for mortality; international	1221 total subjects (100% Ms) 45-64 ys at the 5-y follow-up in 1965 (mean: 54.9 ys, SD: 5.0 ys); at 20-y follow-up CHD events were 185 (fatal and non-fatal); at 40-y follow-up deaths were 187 for CHD, 513 for CVD, 324 for cancer, and 1148 for all-cause mortality	Dietary history; Italian Rural Areas administered at the 5-y follow-up in 1965;<br>IA<br>Validated<br>NA FIs (17 FGs)	CHD incidence in 20 ys; CHD, CVD, cancer, and overall mortality in 40 ys
Menotti, 2018 (63)<br>Italian Rural Areas of Seven Countries Study of Cardiovascular Disease<br>Seven Countries Study<br>Very good quality	Prospective cohort study; enrollment in 1960 from the Italian Rural Areas cohorts, follow-up of 40 ys for mortality; comparison of the role of 4 dietary scores in a sample of middle-aged men followed up during 40 ys for CHD mortality; international	1284 total subjects with final sample size equal to 1214 after excluding 70 subjects with major prevalent CHD (100% Ms); 45-64 ys at the 5-y follow-up in 1965; at 40-y follow-up deaths were 200 from CHD	Dietary history<br>IA<br>Validated<br>NA FIs (17 FGs)	CHD mortality

Maugeri, 2019 (64)<br>Mamma & Bambino<br>Catania (Sicily)<br>Fair quality	Cross-sectional study nested within the "Mamma & Bambino" birth cohort of pregnant women referring to “Policlinico Vittorio Emanuele” (Catania, Italy) for the prenatal genetic counselling without pre-existing medical conditions and/or pregnancy complications; recruitment from November 2014 to 2019 (ongoing at publication); single center/area	332 total subjects (100% Fs); 15-50 ys (median: 37 ys, NA); gestational age at recruitment 4-20 gwks (median: 16 gwks, NA)	FFQ<br>1 mo before<br>IA<br>Adapted from a previously validated FFQ<br>95 FIs (39 FGs)	Socio-demographic (i.e., age, education level, and employment status), lifestyle (i.e., smoking status, pre-gestational BMI, use of folic acid, multivitamin and/or multi-mineral supplements) factors
Maugeri, 2019 (65)<br>Mamma & Bambino<br>Catania (Sicily)<br>Fair quality	Cross-sectional study nested within the "Mamma & Bambino" birth cohort enrolling pregnant women referring to “Policlinico-Vittorio Emanuele” (Catania) at 4–20 gwks (median: 16 gwks) with additional exclusion criteria related to the current article; single center/area	232 total subjects (100% Fs); 15-50 ys (median: 37 ys, NA)	FFQ<br>1 mo before<br>IA<br>Adapted from a previously validated FFQ<br>95 FIs (39 FGs)	Pre-gestational BMI and total GWG
Magnano San Lio, 2022 (66)<br>Catania (Sicily)<br>Good quality	Cross-sectional analysis of data from two prospective cohorts; pregnant women enrolled before COVID-19 pandemic (“Mamma & Bambino” cohort, from November 2014 to December 2019, during the prenatal genetic counseling) and during COVID-19 pandemic ("MAMI-MED", from December 2020 to January 2022, during the first trimester visit) in two hospitals in Catania with the aim to evaluate how their dietary habits affect the health of mother-child pairs; Italian multicentric	1097 total subjects (100% Fs); 397 "Mamma & Bambino" (median: 37.0 ys, IQR: 4.0 ys); 801 "MAMI-MED" (median: 31.0 ys, IQR: 7.0 ys)	FFQ for both studies<br>1 mo before<br>IA<br>Adapted from a previously validated FFQ<br>95 FIs (39 FGs)	Impact of COVID-19 pandemic on the adherence to DPs in pregnant women

Ojeda-Granados, 2022 (67)<br>Catania (Sicily), Guadalajara (Mexico)<br>Fair quality	Cross-sectional study; age-matched Italian non-pregnant women with no history of severe diseases recruited among those referring to three clinical laboratories in Catania (Italy) from 2010 to 2017 and from the general adult population referring to University of Guadalajara (Mexico) from 2011 to 2015; international	1026 total subjects (100% Fs), age 18-72 ys; 811 Italian subjects (median: 40 ys, IQR: 19 ys); 215 Mexican subjects (median: 40 ys, IQR: 21 ys)	Italian FFQ: 1 mo before, IA, Adapted from a previously validated FFQ, 95 FIs (39 FGs);<br>Mexican FFQ: NA reference period, IA, 64 FIs (20 FGs)	No outcomes, DP drivers, or correlates of interest;<br>Combined tertile-based categories of adherence to two identified DPs
Barchitta, 2018 (68)<br>Catania (Sicily)<br>Good quality	Cross-sectional study; women diagnosed with an abnormal PAP test without previous treatments and referred to a cervical cancer screening unit in Catania, later classified according to hrHPV status and histological grade of CIN (from normal cervical epithelium to CIN3); recruitment from 2013 to 2015; single center/area	539 total subjects (100% Fs) of which 252 with normal cervical epithelium and 160 CIN1 (i.e., low-grade CIN); 84 hrHPV infections (+) (mean: 38.63 ys, SD: 10.53 ys) among the 251 (as reported in the text) with a normal cervical epithelium; 167 hrHPV infections (-) (mean: 43.65 ys, SD: 9.62 ys) among the 251 (as reported in the text) with a normal cervical epithelium; 127 CIN2+ (mean: 36.01 ys, SD: 8.10 ys); 411 with normal cervical epithelium or CIN1 (as reported in the text) (mean: 41.50 ys, SD: 10.21 ys)	FFQ<br>1 mo before<br>IA<br>Validated<br>95 FIs (39 FGs)	hrHPV status (positive: infection by any of the 13 identified hrHPV types; negative: otherwise) and high-grade CIN incidence (CIN2+ which included CIN2, CIN3, or carcinoma in situ; ≤ CIN1 which included CIN1 or normal cervical epithelium)
Barchitta, 2019 (69)<br>Catania (Sicily)<br>Good quality	Cross-sectional study; non-pregnant women with no history of severe diseases referring for routine physical examination to three clinical laboratories in Catania; recruitment from 2010 to 2017; single center/area	349 total subjects (100% Fs); age 12-87 ys (median: 36 ys, NA)	FFQ<br>1 mo before<br>IA<br>Adapted from a previously validated FFQ<br>95 FIs (39 FGs)	Leukocyte LINE-1 methylation (surrogate marker of global DNA methylation)

Barchitta, 2019 (70)<br>Eastern Sicily<br>Fair quality	Cross-sectional study; adolescents attending three high schools in the urban area of Eastern Sicily; single center/area	213 total subjects; age 15-18 ys (median: 16 ys; IQR: 0 ys); 102 Ms (median: 16 ys, IQR: 0 ys), 111 Fs (median: 16 ys, IQR: 1 y)	FFQ<br>NA<br>SA<br>Adapted from a previously validated FFQ<br>95 FIs (36 FGs)	School performance assessed through school marks by using the previous y as reference (Italian, English, History, Science, PE, Mathematics, Comportment, and GPA were used as indicators)
Fernández-Alvira, 2014 (71)<br>Italy, Estonia, Cyprus, Belgium, Sweden, Hungary, Germany, and Spain<br>IDEFICS<br>Good quality	Cross-sectional analysis nested within a prospective cohort study of children aged 2–9 ys from 8 European countries (recruited between September 2007 and May 2008) with the aim to investigate the etiology of obesity and the possible interventions for its prevention; international	14233 total subjects (8028 Ms, 6205 Fs; 2-9 ys, of which 12462 with complete dietary and socioeconomic information; mean: 6.0 ys, SD: 1.8 ys at baseline); Italy 2110 subjects (NA Ms, NA Fs)	Same FFQ across all centers (Children's Eating Habits Questionnaire-FFQ)<br>1 mo before<br>IA<br>Reproducible and valid<br>43 FIs (14 FGs) to investigate the consumption frequency of obesity-related foods	SES (as an additive indicator constructed by Bamman et al. including equalized household income, parental education, and occupational position, and ranging from 3 for low SES to 15 for high SES)
Naska, 2006 (72)<br>Belgium, France, Finland, Germany, Greece, Italy, Norway, Portugal, Spain, UK<br>DAFNE<br>Fair quality	Analysis of standardized and post-harmonized data collected through the national household budget surveys undertaken in 10 European countries during the 1990s (Italy 1996) on food, goods, and services available to household members during the reference period conducted by the National Statistical Offices of each country; international	94564 original subjects (NA Ms, NA Fs), age from 0 to over 75 ys, of which 15251 were excluded because they did not fit the pre-defined categories; Italy: 22740 original subjects (NA Ms, NA Fs) of which 16% (3638 subjects) was excluded	No dietary assessment tool used; collected data were availability of foods and beverages at the household level taking into consideration the households' purchases, contributions from all production and food items offered to members as gifts;<br>56 detailed original FGs further aggregated into 25 final FGs	Socio-demographic characteristics: locality (rural, semi-urban, and urban), education level of the household head (elementary, secondary, and higher education), occupation of the household head (manual, non-manual, retired, and other), and household composition (single adult households, two adult households, lone parent households, adults and children households, single elderly households, and 2 members elderly households)

Bravi, 2021 (73)<br>Turin (Piemonte), Florence (Tuscany), Rome (Lazio), San Giovanni Rotondo (Apulia), Palermo (Sicily)<br>MEDIDIET<br>Fair quality	Cross-sectional study; exclusively breastfeeding and healthy women recruited in 5 hospital settings in northern, central and southern Italy had information on dietary habits and a sample of freshly expressed foremilk collected at 6±1 wks post-partum; recruitment between 2012 and 2014; Italian multicentric	300 total subjects (100% Fs), age 25-41 ys (mean: 33 ys, SD: 4.06 ys)	FFQ at 6±1 wks post-partum, same d of milk collection<br>From partum to d of milk collection<br>IA<br>Reproducible and valid<br>78 FIs (31 NUTs)	Foremilk macronutrients and FAs composition
Lasalvia, 2021 (74)<br>Varese (Lombardy)<br>ROCAV<br>Good quality	Cross-sectional study; men and women randomly selected among residents of the Varese city (Lombardy) without main chronic diseases with the aim to investigate the relation between dietary patterns and arterial stiffness; recruitment between 2013 and 2016; single center/area	2640 total subjects (mean: 65.5 ys, SD: 6.7 ys); 1608 Ms (50-75 ys), 1032 Fs (60-75 ys)	FFQ<br>1 y before<br>SA<br>Reproducible and valid<br>188 FIs (41 FGs)	CVD and metabolic risk factors, and carotid-femoral PWV levels as arterial stiffness indicator
Zupo, 2020 (75)<br>Castellana Grotte (Apulia)<br>Salus in Apulia Study (from MICOL study)<br>Very good quality	Prospective cohort study originally enrolling participants from Apulia based center of MICOL study in 1985, with a follow-up for mortality until December 31, 2017; single center/area	2472 total subjects (1429 Ms, 1043 Fs); age > 30 ys (mean: 48.00 ys, SD: 10.71 ys) in a representative sample of the population of Apulia in 1985; 990 total deaths, no additional information on causes	FFQ administered in 1985<br>1 y before<br>SA<br>Validated<br>31 FIs (29 FGs)	Overall and cause-specific (cancer, CVD, and cerebrovascular disease) mortality
Tatoli, 2022 (76)<br>Castellana Grotte (Apulia)<br>Salus in Apulia Study (including also a major part of MICOL study participants)<br>Poor quality	Cross-sectional study a part of which nested within the MICOL cohort; investigated dietary differences between subjects with and without diabetes among non-institutionalized older adults from Southern Italy, recruited between 2014 and 2018, based on health registry office list at December 31, 2014, as well as previous MICOL study participants; single center/area	1399 total subjects (mean: 73.43 ys, SD: 6.30 ys); 187 diabetic subjects (115 Ms, 72 Fs; mean: 74.66 ys, SD: 6.39 ys); 1212 non-diabetic subjects (634 Ms, 578 Fs; mean: 73.24 ys, SD: 6.26 ys)	FFQ administered between 2014 and 2018<br>1 y before<br>SA with interviewer checks<br>Validated<br>85 FIs (28 FGs)	No outcomes, DP drivers, or correlates of interest;<br>Evaluation of internal reproducibility of identified DPs (i.e., between subjects with and without diabetes)

Giontella, 2019 (77)<br>Verona (Veneto)<br>Good quality	Cross-sectional study; children were recruited from the third and fourth classes of four primary schools in the Verona South district with the aim to assess the relationship between food, PA, and main CVD risk factors; single center/area	300 total subjects (7-10 ys); 150 Ms (mean: 8.7 ys, SD: 0.8 ys), 150 Fs (mean: 8.6 ys, SD: 0.7 ys)	FFQ<br>NA<br>NA<br>Validated<br>61 FIs (10 FGs)	CVD risk factors including anthropometric, gluco-lipid, and hemodynamic parameters
Turroni, 2021 (78)<br>Emilia-Romagna (Italy)<br>Good quality	Pilot intervention study; based on Istituto Romagnolo per lo Studio dei Tumori "Dino Amadori" (Meldola, Emilia Romagna) recruitment from October 2018 to September 2019; 60 subjects with at least one among abdominal obesity, hypertension, dyslipidemia, impaired fasting glucose or insulin resistance, 33 of which consumed symbiotic agriculture food (SA-group) and 27 of which received probiotic supplementation (PROB-group) over 30 ds, with a follow-up of 15 ds and stool, urine, and blood samples collected over time; single center/area	60 total subjects (13 Ms, 47 Fs) 18.3-86.4 ys (median age at enrollment: 46.9 ys, IQR: NA); 33 subjects in SA-group (5 Ms, 28 Fs) 34.6-86.4 ys (median age at enrollment: 52.7 ys, IQR: NA); 27 subjects in PROB-group (8 Ms, 19 Fs) 18.3-64.2 ys (median age at enrollment: 45.3 ys, IQR: NA)	FFQ<br>1 y before<br>IA<br>Reproducible ad valid<br>188 FIs (27 NUTs)	No outcomes, DP drivers, or correlates of interest;<br>Cluster analysis based on factor scores
Donati Zeppa, 2020 (79)<br>Urbino (Marche)<br>Fair quality	Trial; normal-weight M and F young adults were recruited by the University of Urbino to participate to a 9-wk HIIT program to investigate the role of PA in modulating food choices; single center/area	32 total subjects (21-24 ys at enrollment); 20 Ms (mean: 22.6 ys, SD: 1.7 ys), 12 Fs (mean: 21.5 ys, SD: 0.8 ys)	24HR in association with PHOTOdietometer for portion size estimation from 2 wks before to the end of the training session<br>IA<br>NA<br>NA FIs (16 NUTs)	No outcomes, DP drivers, or correlates of interest<br>Evaluation of identified DPs

Colica, 2017 (80)<br>Catanzaro (Calabria)<br>Fair quality	Cross-sectional study nested within the cohort reported in Mazza et al. 2017; Caucasian, community-dwelling individuals from Calabria, enrolled between 2013 and 2014, without any bone metabolism disfunctions, aged ≥ 65 ys and satisfying additional criteria underwent whole-body-dual X-ray absorptiometry scan, a fasting venous blood collection, and fractures and dietary intake assessments; single center/area	177 total subjects (37% Ms, 63% Fs); age ≥ 65 ys (mean: 70 ys, SD: 4.1 ys); 41 participants had fractures (52 total fractures)	24HR + 7d-DR<br>NA<br>IA<br>NA<br>NA FIs (10 FGs)	Prevalence of (previous) fractures measured as a dichotomous variable (yes vs. no) and as WB-BMD as indicator of fractures risk in the elderly
Mazza, 2017 (81)<br>Catanzaro (Calabria)<br>Good quality	Cross-sectional and longitudinal analysis of a prospective cohort enrolled between 2013 and 2014 including community-dwelling, Caucasian individuals from Calabria, aged ≥ 65 ys, who underwent a neuropsychological assessment (MMSE and ADAS-Cog) at baseline and 1-y follow-up, and satisfied additional criteria (e.g., MMSE>20); dietary guidance to promote a "healthy diet" was given by a dietitian to all participants during follow-up; 1-y follow-up; single center/area	214 total subjects ≥ 65 ys at baseline (mean: 70 ys, SD: 4 ys), 144 of which had complete data on ADAS-Cog at follow-up and were included in the follow-up analysis	24HR + 7d-DR at baseline<br>IA<br>Validated<br>NA FIs (8 FGs + 10 NUTs)	Cognitive decline measured with MMSE and ADAS-Cog at baseline, at 12 mos, and as progression over time for ADAS-Cog
Palli, 2001 (82)<br>Florence (Tuscany)<br>Good quality	Case-control study; in high-risk area for gastric cancer in central Italy, 382 cases and 561 controls recruited from 1985 to 1987 and 142 additional controls at the end of the study period to have a more representative sample; population based; single center/area	943 total subjects; 382 cases (239 Ms, 143 Fs) 30 subjects <50 ys, 130 subjects 50-64 ys; 222 subjects >64 ys; 561 controls (328 Ms, 233 Fs) 122 subjects <50 ys, 188 subjects 50-64 ys, 251 subjects >64 ys	FFQ<br>1 y before<br>NA<br>NA<br>181 FIs (20 NUTs)	Gastric cancer incidence; Attributable risk estimation; Correspondence analysis based on factor scores

Anelli, 2022 (83)<br>Milan (Lombardy), Naples (Campania)<br>GIFt Study<br>Very good quality	Prospective cohort study; Italian healthy normal-weight singleton pregnant women at 20±2 gwks recruited between January 2017 and June 2020 in 3 hospital settings in northern and southern Italy, followed-up until delivery for pregnancy outcomes; Italian multicentric	179 total subjects 20-40 ys at baseline (mean: 31.8 ys, SD: 4.3 ys); 85 enrolled in Milan (mean: 31.7 ys, SD: 4.5 ys); 94 enrolled in Naples (mean: 31.9 ys, SD: 4.1 ys);	7d-DR: at 25±1 gwks, IA by a trained dietitian;<br>FFQ: at 29±2 gwks, 3 mos before (second trimester of pregnancy), SA but checked by a trained dietitian, adapted from a previously validated FFQ, 192 FIs (15 FGs)	Maternal biomarkers (red blood cells folate in ng/mL, serum vitamin D in ug/L, plasma hepcidin mature form in ng/mL, and plasma total antioxidant capacity in mM) at 29±2 gwks and delivery outcomes (maternal GWG in kg, gestational age at delivery in wks, placental weight in grams, neonatal to placental weight ratio, neonatal ponderal index in grams/cm^3, neonatal length in cm, neonatal weight in grams, and neonatal head circumference in cm)
Ruggieri, 2022 (84)<br>Crotone (Calabria), Milazzo and Augusta-Priolo (Sicily)<br>NEHO Study<br>Good quality	Cross-sectional study nested within a birth cohort; healthy pregnant women with no history of chronic diseases, not requiring special diets, and living in the areas surrounding the perimeter of National Priority Contaminated Sites in Southern Italy were voluntarily recruited starting from January 2018 when admitted to the maternity units of the public hospitals in Milazzo, Syracuse (for the Augusta-Priolo area) and Crotone; Italian multicentric	816 total subjects (100% Fs), age 18-40 ys (mean: 30.6 ys, SD: 5.1 ys); 534 Augusta-Priolo (mean: 30.4 ys, SD ± 5.1 ys); 165 Crotone (mean: 30.5 ys, SD: 5.4 ys); 117 Milazzo (mean: 31.5 ys, SD: 4.5 ys); 589 subjects with available data for risk perception analyses	FFQ<br>Gestational period until FFQ administration (from 32 gwks onwards)<br>IA<br>Not validated<br>41 FIs (38 FGs)	No outcomes, DP drivers, or correlates of interest;<br>Cluster analysis based on principal component scores

ABBREVIATONS: 24HR, 24-hour recall; ADAS-Cog, Alzheimer's Disease Assessment Scale - Cognitive sub-scale; ATBC, Alpha-Tocopherol Beta-Carotene Cancer; BMI, body mass index; CHD, coronary heart disease; CIN, cervical intraepithelial neoplasia; COVID-19, Coronavirus disease 2019; CRP, C-reactive protein; CVD, cardiovascular disease; d, day(s); DAFNE, Data Food Networking; DAS28-CRP, Disease Activity Score on 28 joints and C-reactive protein; DBP, diastolic blood pressure; DIETSCAN, Dietary Patterns and Cancer; DP, dietary pattern; DR, dietary record; EI, energy intake(s); EPIC, European Prospective Investigation into Cancer and Nutrition; F, female(s); FFQ, Food Frequency Questionnaire; FG, food group(s); FI, food item(s); FL, factor loading(s); FSIQ, full scale intelligence quotient; GIFt, Gestational Intake of Food towards healthy outcomes; GPA, Grade Point Average; GWG, gestational weight gain; gwk, gestational week(s); HDL, high-density lipoprotein; HER2, human epidermal growth factor receptor 2; HIIT, high intensity interval training; HR, hazard ratio; hrHPV, high-risk Human Papilloma Virus; IA, interviewer administered; IDEFICS, Identification and prevention of Dietary- and lifestyle-induced health EFfects In Children and infantS; IQR, interquartile range; LDL, low-density lipoprotein; LINE-1, Long Interspersed nuclear elements-1; M, male(s); MAMI-MED, Multisettoriale Alla salute Materno-Infantile Mediante valutazione dell'Esposoma nelle Donne; MMSE, Mini Mental State Examination; mo, month(s); NA, not available; NAC-II, Northern Adriatic Cohort II; NEHO, Neonatal Environment and Health Outcomes; NLSC, Netherlands Cohort Study; NUT, nutrient(s); ORDET, Ormoni e Dieta nell'Eziologia del Tumore della Mammella; PA, physical activity; PCA, Principal Component Analysis; PE, Physical Education; PRI, perceptual

reasoning index; PSI, processing speed index; PWV, pulse wave velocity; ROCAV, Risk Of Cardiovascular diseases and abdominal aortic Aneurysm in Varese; SA, self-administered; SBP, systolic blood pressure; SD, standard deviation; SDAI, Simplified Disease Activity Index; SE, standard error; SES, socioeconomic status; SF-36, Short Form Healthy Survey 36; SMC, Swedish Mammography Cohort; TG, triglyceride(s); VCI, verbal comprehension index; vs., versus; WB-BMD, whole-body bone mineral density; WISC-IV, Wechsler Intelligence Scale of Children; wk, week(s); WMI, working memory index; y, year(s)

Supplemental Table 2. Dietary patterns identified using principal component and factor analyses in Italy and their association with disease outcomes/dietary pattern drivers/correlates of interest.

Reference, location, study name, study quality	Dietary pattern identification methods	Expl. Var. % (NF)	Dietary pattern composition	Models and tests	Adjustments	Association with disease outcomes/dietary pattern drivers/correlates of interest
Edefonti, 2008 (33) Breast cancer: northern Italy (Milan, Genoa, Gorizia, Forli), central and southern Italy (Latina, Naples) Ovarian cancer: northern Italy (Milan, Pordenone, Padua), central and southern Italy (Latina, Naples) Good quality	PCFA Standardization EIG>1, Scree plot, and interpretability Varimax rotation |FL|≥0.63 Factorability checks NA DP internal consistency NA DP reproducibility (internal)	75.70% (4)	ANIMAL PRODUCTS: animal protein and animal fat, calcium, cholesterol, SFAs, riboflavin, zinc, and phosphorus; VITAMINS AND FIBER: vitamin C and total fiber, total folate, potassium, beta-carotene equivalents, soluble carbohydrates, and vitamin B6; UNSATURATED FAT: vegetable fat and vitamin E, MUFAs and PUFAs; STARCH-RICH: starch, vegetable protein, and sodium	Multiple logistic regression models on quartiles of factor scores for all DPs as independent variables	Adjusted for age, education, parity, menopausal status, family history of digestive cancers, family history of female cancers, BMI, geographic area, and total EI	ANIMAL PRODUCTS: OR 0.74 (95%CI: 0.61–0.91) of breast cancer for 4th vs. 1st quartile, p-trend<0.01; VITAMINS AND FIBER: OR 0.77 (95%CI: 0.61–0.98) of ovarian cancer for 4th vs. 1st quartile, p-trend=0.026; UNSATURATED FAT: OR 0.83 (95%CI: 0.68–1.00) of breast cancer for 4th vs. 1st quartile, p-trend=0.03 STARCH-RICH: OR 1.34 (95%CI: 1.10–1.65) of breast cancer for 4th vs. 1st quartile, p-trend<0.01, and OR 1.85 (95%CI: 2.37–2.48) of ovarian cancer for 4th vs. 1st quartile, p-trend=0.03; Other DPs did not provide additional evidence
Bertuccio, 2009 (34) Milan	PCFA Standardization	75.09% (4)	ANIMAL PRODUCTS: animal protein, riboflavin, cholesterol,	Multiple logistic regression models on	Conditioned on age and sex; adjusted for quinquennia of	ANIMAL PRODUCTS: OR 2.13 (95%CI: 1.34–3.40) of gastric cancer for 4th vs. 1st quartile, p-trend=0.0003;

(Lombardy)<br>Good quality	EIG>1, Scree plot, and interpretability<br>Varimax rotation<br>|FL|≥0.63<br>Factorability checks<br>DP internal consistency<br>DP reproducibility (internal)		phosphorus, calcium, and zinc;<br>VITAMINS AND FIBER: vitamin C, total fiber, potassium, total folate, beta-carotene equivalents, and soluble carbohydrates;<br>VUFA: other PUFAs, vitamin E, MUFAs, LA, and ALA;<br>STARCH-RICH: starch, vegetable protein, and sodium	quartiles of factor scores for all DPs as independent variables	period of interview, education, BMI, tobacco smoking, and family history of gastric cancer	VITAMINS AND FIBER: OR 0.60 (95%CI: 0.37–0.99) of gastric cancer for 4th vs. 1st quartile, p-trend=0.0861;<br>STARCH-RICH: OR 1.67 (95%CI: 1.01–2.77) of gastric cancer for 4th vs. 1st quartile, p-trend=0.0463;<br>Other DPs did not provide additional evidence
Edefonti, 2010 (35)<br>Milan (Lombardy), Pordenone (Friuli Venezia Giulia), Rome, Latina (Lazio)<br>Good quality	PCFA<br>Standardization<br>EIG>1, Scree plot, and interpretability<br>Varimax rotation<br>|FL|≥0.63<br>Factorability checks<br>DP internal consistency<br>DP reproducibility (internal)	79.94% (5)	ANIMAL PRODUCTS: animal fat, calcium, SFAs, animal protein, phosphorus, cholesterol, and riboflavin;<br>VITAMINS AND FIBER: vitamin C, total fiber, soluble carbohydrates, and beta-carotene equivalents;<br>UNSATURATED FATS: vegetable fat and vitamin E, MUFAs and PUFAs;<br>RETINOL AND NIACIN: retinol and niacin;<br>STARCH-RICH: starch, vegetable protein, and sodium	Multiple logistic regression models on quintiles of factor scores computed among controls for all DPs as independent variables	Adjusted for age, sex, study center, education, BMI, tobacco smoking, and alcohol drinking	ANIMAL PRODUCTS: OR 1.56 (95%CI: 1.13–2.15) of oral and pharyngeal cancer for 5th vs. 1st quintile, p-trend<0.001;<br>VITAMINS AND FIBER: OR 0.47 (95%CI: 0.34–0.65) of oral and pharyngeal cancer for 5th vs. 1st quintile, p-trend<0.001;<br>UNSATURATED FATS: OR 0.63 (95%CI: 0.45-0.86) of oral and pharyngeal cancer for 5th vs. 1st quintile, p-trend=0.03;<br>STARCH RICH: OR 0.71 (95%CI: 0.50–0.99) of oral and pharyngeal cancer for 5th vs. 1st quintile, p-trend=0.06;<br>Other DPs did not provide additional evidence
Bravi, 2010 (36)<br>Milan	PCFA<br>Standardization	81.36% (5)	ANIMAL PRODUCTS: calcium, animal protein, phosphorus,	Multiple logistic regression models on	Adjusted for age, sex, study center, education,	VITAMINS AND FIBER: OR 0.61 (95%CI: 0.48–0.77) of rectal cancer for 4th vs. 1st quartile, p-trend<0.0001;

(Lombardy); Genoa (Liguria), Pordenone, Gorizia (Friuli Venezia Giulia), Forlì (Emilia-Romagna), Latina (Lazio), Naples (Campania)<br>Good quality	EIG>1, Scree plot, and interpretability<br>Varimax rotation<br>|FL|≥0.63<br>Factorability checks<br>DP internal consistency<br>DP reproducibility (internal)		riboflavin, SFAs, and cholesterol;<br>VITAMINS AND FIBER: vitamin C, total fiber, beta-carotene equivalents, soluble carbohydrates, and total folate;<br>VUFA: LA, ALA, and vitamin E;<br>AUFA: other PUFAs and vitamin D;<br>STARCH-RICH: starch, vegetable protein, and sodium	quartiles of factor scores for all DPs as independent variables; stratified analysis by sex; separate analyses by colon and rectal cancer	occupational PA, and family history of colorectal cancer	VUFA: OR 0.79 (95%CI: 0.65–0.96) of colon cancer for 4th vs. 1st quartile, p-trend=0.0281, and OR 1.27 (95%CI: 1.00–1.62) of rectal cancer for 4th vs. 1st quartile, p-trend=0.0831;<br>AUFA: OR 0.80 (95%CI: 0.66–0.98) of colon cancer for 4th vs. 1st quartile, p-trend=0.0232;<br>STARCH-RICH: OR 1.68 (95%CI: 1.37–2.07) of colon cancer for 4th vs. 1st quartile, p-trend<0.0001, and OR 1.74 (95%CI: 1.34–2.26) of rectal cancer for 4th vs. 1st quartile, p-trend=0.0001;<br>Other DPs did not provide additional evidence; comparable results were observed in the stratified analyses by sex (data not provided in the article)
Edefonti, 2010 (37)<br>Milan (Lombardy), Pordenone (Friuli Venezia Giulia)<br>Good quality	PCFA<br>Standardization<br>EIG>1, Scree plot, and interpretability<br>Varimax rotation<br>|FL|≥0.63<br>Factorability checks<br>DP internal consistency<br>DP reproducibility (internal)	79.00% (5)	ANIMAL PRODUCTS: calcium, phosphorus, riboflavin, animal protein, SFAs, zinc, and cholesterol;<br>VITAMINS AND FIBER: vitamin C and total fiber, beta-carotene equivalents, and total folate;<br>VUFA: LA, ALA, and vitamin E;<br>AUFA: other PUFAs and vitamin D;<br>STARCH-RICH: starch, vegetable protein, and sodium	Multiple logistic regression models on quartiles of factor scores computed among controls for all DPs as independent variables; stratified analyses by age, education, alcohol drinking, tobacco smoking, BMI; separate analyses by anatomic subsite (i.e., supraglottis, glottis,	Adjusted for age, sex, study center, education, BMI, occupational PA, alcohol drinking, and tobacco smoking, when appropriate	ANIMAL PRODUCTS: OR 2.34 (95%CI: 1.59–3.45) of laryngeal cancer for 4th vs. 1st quartile, p-trend<0.001; OR 2.03 (95%CI: 1.07–3.84) of supraglottis cancer and OR 2.30 (95%CI: 1.51–3.49) of glottis cancer for 3rd vs. 1st tertile;<br>VITAMINS AND FIBER: OR 0.35 (95%CI: 0.24–0.52) of laryngeal cancer for 4th vs. 1st quartile, p-trend<0.001; OR 0.47 (95%CI: 0.25–0.86) of supraglottis cancer, OR 0.57 (95%CI: 0.39–0.84) of glottis cancer, and OR 0.45 (95%CI: 0.26–0.77) of other/unspecified site of laryngeal cancer for 3rd vs. 1st tertile; subjects older than 60 ys did not show any protective effect of this DP;<br>VUFA: OR 1.98 (95%CI: 1.00–3.92) of supraglottis cancer for 3rd vs. 1st tertile;<br>AUFA: OR 2.07 (95%CI: 1.42–3.01) of laryngeal cancer for 4th vs. 1st quartile, p-trend <0.001; OR 2.05 (95%CI: 1.10–3.81) of supraglottis cancer, OR 2.02 (95%CI: 1.36–3.01) of glottis cancer, and OR 2.00 (95%CI:

				other/unspecified) on tertiles of each DP		1.16–3.46) of other/unspecified site of laryngeal cancer for 3rd vs. 1st tertile; Starch-rich DP did not provide additional evidence overall or in strata; other DPs did not provide additional evidence based on stratified analyses
Bravi, 2012 (38) Milan (Lombardy), Pordenone (Friuli Venezia Giulia); Padua (Veneto) Good quality	PCFA Standardization EIG>1, Scree plot, and interpretability Varimax rotation |FL|≥0.63 Factorability checks DP internal consistency DP reproducibility (internal)	79.18% (5)	ANIMAL PRODUCTS AND RELATED COMPONENTS: calcium, phosphorus, riboflavin, animal protein, SFAs, cholesterol, and zinc; VITAMINS AND FIBER: vitamin C, total fiber, beta-carotene equivalents, soluble carbohydrates, and total folate; STARCH-RICH: starch, vegetable protein, and sodium; OTHER PUFAs AND VITAMIN D: other PUFAs, vitamin D, and niacin; OTHER FATS: LA, ALA, and vitamin E	Multiple logistic regression models on quartiles of factor scores computed among controls for all DPs as independent variables	Adjusted for age, sex, study center, education, alcohol drinking, tobacco smoking, and BMI	ANIMAL PRODUCTS AND RELATED COMPONENTS: OR 1.64 (95%CI: 1.06–2.55) of esophageal cancer for 4th vs. 1st quartile, p-trend=0.0062; VITAMINS AND FIBER: OR 0.50 (95%CI: 0.32–0.78) of esophageal cancer for 4th vs. 1st quartile, p-trend=0.0002; OTHER PUFAs AND VITAMIN D: OR 0.48 (95%CI: 0.31–0.74) of esophageal cancer for 4th vs. 1st quartile, p-trend=0.0009; Other DPs did not provide additional evidence
Bosetti, 2013 (39) Milan (Lombardy), Pordenone (Friuli	PCFA Standardization EIG>1, Scree plot, and interpretability Varimax	75.84% (4)	ANIMAL PRODUCTS: calcium, animal protein, phosphorus, riboflavin, SFAs, cholesterol, and zinc; VITAMINS AND FIBER: vitamin C, total	Conditional multiple logistic regression models on quartiles of factor scores computed	Conditioned on age (categorical), sex, and study center; adjusted for y of interview (continuous), education	ANIMAL PRODUCTS: OR 2.03 (95%CI: 1.29–3.19) of pancreatic cancer for 4th vs. 1st quartile, p-trend=0.0008; VITAMINS AND FIBER: OR 0.55 (95%CI: 0.35–0.86) of pancreatic cancer for 4th vs. 1st quartile, p-trend=0.0035; STARCH-RICH: OR 1.69 (95%CI: 1.02–

Venezia Giulia) Good quality	rotation |FL|≥0.63 Factorability checks DP internal consistency DP reproducibility (internal)		fiber, beta-carotene equivalents, soluble carbohydrates, total folate, and potassium; UNSATURATED FATS: LA, vitamin E, ALA, and other PUFAs; STARCH-RICH: starch, vegetable protein, and sodium	among controls for all DPs as independent variables	(categorical), BMI (categorical), tobacco smoking (categorical, combination of status and intensity), alcohol drinking (categorical, intensity), and diabetes (categorical)	2.79) of pancreatic cancer for 4th vs. 1st quartile, p-trend=0.0592; Other DPs did not provide additional evidence
Rosato, 2014 (40) Milan (Lombardy), Pordenone, Gorizia (Friuli Venezia Giulia), Latina (Lazio), Naples (Campania) Good quality	PCFA Standardization EIG>1, Scree plot, and interpretability Varimax rotation |FL|≥0.63 Factorability checks DP internal consistency DP reproducibility (internal)	78.27% (5)	ANIMAL PRODUCTS: calcium, phosphorus, riboflavin, animal protein, SFAs, zinc, and cholesterol; VITAMINS AND FIBER: vitamin C, total fiber, beta-carotene equivalents, total folate, and soluble carbohydrates; VUFA: LA, vitamin E, and ALA; AUFA: other PUFAs and vitamin D; STARCH-RICH: starch, vegetable protein, and sodium	Multiple logistic regression models on quintiles of factor scores for all DPs as independent variables	Adjusted for age (categorical), study center, education (categorical), BMI (categorical), tobacco smoking (categorical, combination of status and intensity), alcohol drinking (categorical, combination of status and intensity), and family history of prostate cancer in first-degree relatives (categorical)	ANIMAL PRODUCTS: OR 1.51 (95%CI: 1.16–1.96) of prostatic cancer for 4th vs. 1st quartile, p-trend=0.02; AUFA: OR 1.32 (95%CI: 1.02–1.70) of prostatic cancer for 4th vs. 1st quartile, p-trend=0.02; STARCH-RICH: OR 1.50 (95%CI: 1.16–1.93) of prostatic cancer for 4th vs. 1st quartile, p-trend<0.001; Other DPs did not provide additional evidence

Bravi, 2015 (41) Milan (Lombardy), Pordenone, Udine (Friuli Venezia Giulia), Naples (Campania) Good quality	PCFA Standardization EIG>1, Scree plot, and interepretability Varimax rotation |FL|≥0.63 Factorability checks DP internal consistency DP reproducibility (internal)	80.04% (5)	WESTERN TYPE DIET: calcium, riboflavin, phosphorus, animal protein, SFAs, cholesterol, and zinc; VITAMINS AND FIBER: vitamin C, total fiber, potassium, total folate, beta-carotene equivalents, and soluble carbohydrates; STARCH-RICH: starch, vegetable protein, and sodium; ANIMAL DERIVED NUTRIENTS AND PUFA: vitamin D, other PUFAs, and niacin; OTHER FATS: LA, ALA, and vitamin E	Conditional multiple logistic regression models on quartiles of factor scores computed among controls for all DPs as independent variables; Stratified analyses by age (<60 ys, ≥ 60 ys) and BMI (<30 kg/m^2, ≥30 kg/m^2)	Conditioned on age and study center; adjusted for period of interview, education, BMI, history of diabetes, age at menarche, menopausal status, parity, OC and HRT use	WESTERN TYPE DIET: OR 1.63 (95%CI: 1.12–2.38) of endometrial cancer for 4th vs. 1st quartile, p-trend=0.0058; OR 1.45 (95%CI: 0.93–2.26) of endometrial cancer for 4th vs. 1st quartile among subjects with BMI<30 and OR 2.08 (95%CI: 0.92–4.69) of endometrial cancer for 4th vs. 1st quartile among subjects with BMI≥30 (p-heterogeneity<0.0001); non-significant heterogeneity across strata by age; ANIMAL DERIVED NUTRIENTS AND PUFA: OR 1.76 (95%CI: 1.23–2.52) of endometrial cancer for 4th vs. 1st quartile; p-trend=0.0004; OR 1.75 (95%CI: 1.16–2.64) of endometrial cancer for 4th vs. 1st quartile among subjects with BMI<30 and OR 2.30 (95%CI: 1.03–5.16) of endometrial cancer for 4th vs. 1st quartile among subjects with BMI≥30 (p-heterogeneity<0.0001); non-significant heterogeneity across strata by age; Other DPs did not provide additional evidence
Edefonti, 2015 (42) Milan (Lombardy), Pordenone (Friuli Venezia Giulia), Naples (Campania), Catania (Sicily) Good quality	PCFA Standardization EIG>1, Scree plot, and interpretability Varimax rotation |FL|≥0.63 Factorability checks DP internal consistency DP reproducibility (internal)	79.60% (5)	ANIMAL PRODUCTS: calcium, riboflavin, phosphorus, SFAs, animal protein, and cholesterol; VITAMINS AND FIBER: vitamin C and total fibre, beta-carotene equivalents, and total folate; VUFA: LA, ALA, and vitamin E; AUFA: other PUFAs and vitamin D; STARCH-RICH:	Multiple logistic regression models on tertiles of factor scores computed among controls for all DPs as independent variables; Stratified analyses by age, tobacco smoking status, and alcohol	Adjusted for age, sex, area of residence, education, y of interview, alcohol drinking, and tobacco smoking	ANIMAL PRODUCTS: OR 2.62 (95%CI: 1.67–4.13) of nasopharyngeal cancer for 3rd vs. 1st tertile, p-trend<0.001; STARCH-RICH: OR 2.05 (95%CI: 1.27–3.33) of nasopharyngeal cancer for 3rd vs. 1st tertile, p-trend=0.022; AUFA: OR 1.55 (95%CI: 1.00–2.39) of nasopharyngeal cancer for 3rd vs. 1st tertile, p-trend=0.038; VUFA: OR 1.90 (95%CI: 1.22–2.96) of nasopharyngeal cancer for 3rd vs. 1st tertile, p-trend=0.011; VITAMINS AND FIBER: did not provide additional evidence; non-significant heterogeneity across strata for the 5 DPs

			starch, vegetable protein, and sodium	drinking intensity		
Dalmartello, 2020 (43) Milan (Lombardy), Pordenone, Udine (Friuli Venezia Giulia), Latina (Lazio), Naples (Campania) Good quality	PCFA Standardization EIG>1, Scree plot, and interpretability Varimax rotation |FL|≥0.63 Factorability checks DP internal consistency DP reproducibility (internal)	74.52% (4)	ANIMAL PRODUCTS: calcium, animal protein, riboflavin, phosphorus, cholesterol, SFAs, and zinc; VITAMINS AND FIBER: vitamin C, total fiber, soluble carbohydrates, beta-carotene equivalents, potassium, and total folate; COOKING OIL AND DRESSING: vitamin E, LA, and ALA; STARCH-RICH: starch, vegetable protein, and sodium	Conditional multiple logistic regression models on quartiles of factor scores for all DPs as independent variables computed among controls; Stratified analyses by education, history of hypertension, BMI, tobacco smoking, and alcohol drinking	Conditioned on study center, sex and quinquennia of age; adjusted for ys of education (categorical), period of interview (categorical), family history of kidney cancer in first-degree relatives (categorical), hypertension (categorical), tobacco smoking (categorical, combination of status and intensity), alcohol drinking (categorical, combination of status and intensity), and BMI (categorical)	STARCH-RICH: OR 1.38 (95%CI: 1.04–1.82) of renal cell cancer for 4th vs. 1st quartile, p-trend=0.018; COOKING OILS AND DRESSING: OR 0.61 (95%CI: 0.47–0.80) of renal cell cancer for 4th vs. 1st quartile, p-trend<0.001; Other DPs did not provide additional evidence; significant heterogeneity was observed across strata of BMI and tobacco smoking for the VITAMINS AND FIBER DP, and of tobacco smoking for the ANIMAL PRODUCTS DP, but no significant results were found in strata
Edefonti, 2020 (44) Milan (Lombardy), Pordenone (Friuli Venezia Giulia), Naples	PCFA Standardization EIG>1, Scree plot, and interpretability Varimax rotation |FL|≥0.63 Factorability	78.09% (4)	ANIMAL PRODUCTS: calcium, SFAs, riboflavin, animal protein, cholesterol, phosphorus, and zinc; VITAMINS AND FIBER: vitamin C, total fiber, beta-carotene equivalents, vitamin E, potassium, and total	Multiple logistic regression models on quartiles of factor scores for all DPs as independent variables	Adjusted for age (categorical), sex, center of recruitment, education (categorical), cigarette smoking (categorical, combination of status and	ANIMAL PRODUCTS: OR 0.70 (95%CI: 0.48–1.01) of bladder cancer for 4th vs. 1st quartile, p-trend=0.026; VITAMINS AND FIBER: OR 0.70 (95%CI: 0.49–0.98) of bladder cancer for 4th vs. 1st quartile, p-trend=0.109; Other DPs did not provide additional evidence

(Campania), Catania (Sicily) Good quality	checks DP internal consistency DP reproducibility (internal)		folate; AUFA: other PUFAs and vitamin D; STARCH-RICH: starch, vegetable protein, and sodium		intensity), alcohol drinking intensity (categorical), history of occupational exposure in selected sectors relevant for bladder cancer risk (categorical), history of diabetes, history of cystitis, family history of bladder cancer, y of interview, and BMI	
Edefonti, 2020 (45) Milan (Lombardy) Good quality	PCFA Standardization EIG>1, Scree plot, and interpretability Varimax rotation |FL|≥0.63 Factorability checks DP internal consistency DP reproducibility (internal)	79.85% (5)	ANIMAL PRODUCTS: cholesterol and SFAs; ANTI-OXIDANT VITAMINS AND FIBER: soluble carbohydrates, potassium, vitamin C, vitamin A (Retinol Activity Equivalent), soluble and insoluble fiber, lignans, and flavonoids; VUFA: LA, ALA, and vitamin E; AUFA: EPA and DHA, and vitamin D; STARCH-RICH: total protein, starch, sodium, phosphorus, iron, zinc, magnesium, selenium, and vitamin B1 and B3	Multiple logistic regression models on tertiles of factor scores for all DPs as independent variables with DAS28-CRP and SDAI as dependent variables dichotomized in "presence of disease activity" or "remission"; Robust linear regression models on tertiles of factor scores for all DPs as independent variables with	Adjusted for age (categorical), sex, education (categorical), BMI (categorical), cigarette smoking status (categorical), alcohol drinking intensity (categorical), disease duration (categorical), RF (categorical), ACPA (categorical), presence of any therapy (categorical), conventional synthetic DMARDs (categorical), biologic DMARDs (categorical), targeted synthetic DMARDs	ANIMAL PRODUCTS: not associated with overall disease activity; heterogeneity among strata by RF and/or ACPA positivity and among strata by disease duration was detected in the robust linear regression model with SDAI for the highest vs. the lowest tertile-based category of adherence (p-heterogeneity=0), but associations pointed in the same direction (inverse) were not significant in all strata; ANTI-OXIDANT VITAMINS AND FIBER: not associated with overall disease activity; heterogeneity among strata by RF and/or ACPA positivity was detected in the robust linear regression model with SDAI for the highest vs. the lowest tertile-based category of adherence (p-heterogeneity=0), but association pointed in the same direction (positive) was not significant in both strata; VUFA: OR 0.39 (95%CI: 0.21–0.74) of rheumatoid arthritis activity according to DAS28-CRP for the highest vs. the lowest tertile-based category of adherence; being in the highest vs. the lowest category of

				DAS28-CRP and SDAI as continuous dependent variables; Stratified analyses by disease severity (i.e., RF and/or ACPA positivity status) and by disease duration (≤15 ys, >15 ys)	(categorical), and steroids (categorical) for both logistic and linear regression models	adherence was associated with decreased DAS28-CRP (beta: -0.36, SE: 0.14, p=0); heterogeneity among strata by disease duration was detected in the robust linear regression model with SDAI for the highest vs. the lowest category of adherence (p-heterogeneity=0), with significant inverse association only with disease duration >15 ys (beta: -4.60, SE: 1.66, p=0);<br>AUFA: OR 0.53 (95%CI: 0.28–1.00) of rheumatoid arthritis activity according to DAS28-CRP for the highest vs. the lowest tertile-based category of adherence; being in the highest vs. the lowest category of adherence was associated with decreased DAS28-CRP (beta: -0.25, SE: 0.14, p=0.05); heterogeneity among strata by RF and/or ACPA positivity and disease duration was detected in the robust linear regression model with SDAI (p-heterogeneity=0), with significant inverse association only with RF and/or ACPA positivity (beta: -3.85, SE: 1.27, p=0) and with disease duration >15 ys (beta: -4.75, SE: 1.61, p=0);<br>STARCH-RICH: not associated with overall disease activity; heterogeneity among strata by disease duration was detected in the robust linear regression model with SDAI highest vs. the lowest tertile-based category (p-heterogeneity=0), but associations pointed in the same direction (inverse) were not significant in both strata
Marinoni, 2022 (46) Croatia, Greece, Italy (Friuli Venezia Giulia	PCFA Standardization<br>EIG>1, Scree plot, and interpretability Varimax	63.39% (5)	DAIRY PRODUCTS: calcium, biotin, magnesium, pantothenic acid, iodine, phosphorus, and vitamin B2; PLANT-BASED	Robust multiple linear regression models for assessing the association between all DPs as independent	Adjusted for father's education (categorical), maternal Raven's test score during pregnancy (continuous), folic	DAIRY PRODUCTS: inversely associated with PSI score (beta: -2.05, SE: 0.84, p-value<0.01);<br>MEAT AND POTATOES: inversely associated with VCI score (beta: -1.28, SE: 0.66, p-value<0.05);<br>SEAFOOD: positively associated with VCI

region)<br>Good quality	rotation<br>|FL|≥0.60<br>Factorability checks<br>DP internal consistency<br>DP reproducibility (internal)		FOODS: total fiber, vitamin C, folate, potassium, beta-carotene, vitamin E, and iron;<br>FATS: MUFAs, oleic acid, SFAs, and LA;<br>MEAT AND POTATOES: niacin, vitamin B6, proteins, vitamin B1, and zinc;<br>SEAFOOD: EPA, DHA, and selenium	variables (continuous variables) and each of FSIQ, VCI, PRI, WMI, and PSI (from WISC-IV) as dependent variable	acid supplementation before pregnancy (categorical), alcohol consumption during pregnancy (units per wk, continuous), breastfeeding (categorical), house property (categorical), child's sex (categorical), child's birth weight ≥4 kg (categorical), child's BMI at 7 ys (categorical), and child's extracurricular PA at 7 ys (categorical)	score (beta: 1.24, SE: 0.64, p-value<0.05) and with PRI score (beta: 1.35, SE: 0.70, p-value<0.05);<br>Other DPs did not provide additional evidence
Centritto, 2009 (47)<br>Molise<br>Moli-sani<br>Good quality	PCFA<br>Standardization<br>EIG>1, Scree plot, and interpretability<br>Varimax rotation<br>|FL|≥0.15<br>Factorability checks NA<br>DP internal consistency NA<br>DP reproducibility (internal)	15.7% (3)	OLIVE OIL AND VEGETABLES: olive oil, cooked and raw vegetables, legumes, soups, fruits, fish, potatoes, bouillon, white meat, crustaceans and molluscs, crisp bread and rusks, nuts and dried fruits, yogurt, snacks, and fresh cheese;<br>PASTA AND MEAT: high on pasta and other grains, cooked tomatoes, red meat, white meat, olive oil,	Multiple linear regression models on quintiles of each factor score as independent variable and total, HDL and LDL cholesterol, SBP, DBP, and log-transformed values for CRP, CUORE project CVD risk score across strata by sex, TGs, and blood glucose in	Adjusted for sex, smoking, SES, age, BMI, total EI and total PA (continuous);<br>Analyses on CRP further adjusted for TGs and performed only where CRP ≤10 mg/L;<br>Analyses on CUORE project CVD risk were only adjusted for smoking, age, and total EI	OLIVE OIL AND VEGETABLES: higher factor scores were associated with reduced total cholesterol (p-trend=0.0006), LDL cholesterol (p-trend=0.039), SBP (p-trend=0.0012), DBP (p-trend=0.0002), CRP (p-trend=0.018), CUORE project CVD risk score in Ms (p-trend<0.0001), TGs (p-trend<0.0001), and blood glucose (p-trend=0.001);<br>PASTA AND MEAT: higher factor scores were associated with increased total cholesterol (p-trend=0.0043), LDL cholesterol (p-trend=0.032), DBP (p-trend=0.098), CRP (p-trend<0.0001), CUORE project CVD risk score in Ms (p-trend=0.0007) and in Fs (p-trend<0.0001), TGs (p-trend=0.0002), and blood glucose (p-trend<0.0001);

			animal fats, other sauces, wine, beer, bread, offals, processed meat, and seasoned cheese; low on breakfast cereals and yogurt;<br>EGGS AND SWEETS: eggs, margarines, processed meat, sugar and sweets, vegetable oils, snacks, mayonnaises, butter, seasoned cheese, fresh cheese, pizza, canned fish, fruit juices, coffee, soft drinks, potatoes, white meat, red meat, animal fats, bread, and beer	the presence of asymmetry		EGGS AND SWEETS: higher factor scores were associated with increased CRP (p-trend<0.0001) and CUORE project CVD risk score in Ms (p-trend=0.041), and with reduced TGs (p-trend=0.044)

Bonaccio, 2012 (48) Molise Moli-sani Good quality	PCFA Standardization EIG>1, Scree plot, and interpretability Varimax rotation |FL|≥0.15 Factorability checks NA DP internal consistency NA DP reproducibility (internal)	NA% (3)	OLIVE OIL AND VEGETABLES: olive oil, cooked and raw vegetables, legumes, soups, fruits, fish, potatoes, bouillon, white meat, crustaceans and molluscs, crisp bread and rusks, nuts and dried fruits, yogurt, snacks, and fresh cheese; PASTA AND MEAT: high on pasta and other grains, cooked tomatoes, red meat, white meat, olive oil, animal fats, other sauces, wine, beer, bread, offals, processed meat, and seasoned cheese; low on breakfast cereals and yogurt; EGGS AND SWEETS: eggs, margarines, processed meat, sugar and sweets, vegetable oils, snacks, mayonnaises, butter, seasoned cheese, fresh cheese, pizza, canned fish, fruit juices, coffee, soft drinks, potatoes, white meat, red meat, animal fats, bread, and beer	Multiple linear regression models with each DP as dependent variable, and categories of household income as independent variable; ANOVA based on the regression models to test the presence of differences between means of factor scores of each DP across categories of household net income; Stratified analyses by age and sex	Adjusted for age, sex, daily EI, BMI, PA, smoking, alcohol consumption, and marital status	OLIVE OIL AND VEGETABLES: mean factor scores were not equal across the 4 categories of income ($p<0.0001$) with mean factor scores reported to be higher in higher categories of income; similar results were observed after stratification by age and sex; EGGS AND SWEETS: mean factor scores were not equal across the 4 categories of income ($p<0.0001$) with mean factor scores reported to be lower in both extreme categories of low and high income; similar results were observed after stratification by age and sex; Other DPs did not provide additional evidence

Bonaccio, 2012 (49) Molise Moli-sani Good quality	PCFA Standardization EIG>1, Scree plot, and interpretability Varimax rotation |FL|≥0.15 Factorability checks NA DP internal consistency NA DP reproducibility (internal)	NA% (3)	OLIVE OIL AND VEGETABLES: olive oil, cooked and raw vegetables, legumes, soups, fruits, fish, potatoes, bouillon, white meat, crustaceans and molluscs, crisp bread and rusks, nuts and dried fruits, yogurt, snacks, and fresh cheese; PASTA AND MEAT: high on pasta and other grains, cooked tomatoes, red meat, white meat, olive oil, animal fats, other sauces, wine, beer, bread, offals, processed meat, and seasoned cheese; low on breakfast cereals and yogurt; EGGS AND SWEETS: eggs, margarines, processed meat, sugar and sweets, vegetable oils, snacks, mayonnaises, butter, seasoned cheese, fresh cheese, pizza, canned fish, fruit juices, coffee, soft drinks, potatoes, white meat, red meat, animal fats, bread, and beer	Multiple linear regression models with each DP as dependent variable, and tertiles of factor scores of mass media exposure (light/moderate/ heavy exposure) as independent variables; ANOVA based on the regression models to test the presence of differences between means of factor scores of each DP across tertiles of factor scores of mass media exposure; Stratified analysis by SES (low/medium/high) and education (higher/lower)	Adjusted for sex, age and additionally for total EI, PA, log-transformed CRP (based on p<0.10 in a series of age and sex adjusted regression models) as well as the other two DPs and SES or education depending on the regression model; stratified models adjusted for sex, age, total EI, PA, log-transformed CRP, and the other two DPs	OLIVE OIL AND VEGETABLES: mean factor scores were not equal across tertiles of mass media exposure in the SES-adjusted (p=0.0018) and in the education-adjusted (p=0.0027) models, with mean factor scores reported to be higher in the heavy mass media exposure group; mean factor scores were not equal across tertiles of mass media exposure when analyses were restricted to the medium SES subgroup (p=0.03), with mean factor scores reported to be higher in the heavy mass media exposure group; mean factor scores were not equal across tertiles of mass media exposure when analyses were restricted to the higher (p=0.015) and lower (p=0.09) education subgroups, with mean factor scores reported to be higher in the heavy mass media exposure group; PASTA AND MEAT: mean factor scores were borderline not equal across tertiles of mass media exposure in the SES-adjusted (p=0.068) and in the education-adjusted (p=0.034) models, with mean factor scores reported to be lower in the heavy mass media exposure group; mean factor scores were not equal across tertiles of mass media exposure when analyses were restricted to the medium SES subgroup (p=0.03), with mean factor scores reported to be higher in the moderate mass media exposure group; mean factor scores were not equal across tertiles of mass media exposure when analyses were restricted to the lower education subgroup (p=0.06), with mean factor scores reported to be lower in the heavy mass media exposure group; Other DPs did not provide additional evidence

Bonaccio, 2013 (50)<br>Molise<br>Moli-sani<br>Good quality	PCFA<br>Standardization<br>EIG>1, Scree plot, and interpretability<br>Varimax rotation<br>|FL|≥0.15<br>Factorability checks NA<br>DP internal consistency NA<br>DP reproducibility (internal)	NA%<br>(3)	OLIVE OIL AND VEGETABLES: olive oil, cooked and raw vegetables, legumes, soups, fruits, fish, potatoes, bouillon, white meat, crustaceans and molluscs, crisp bread and rusks, nuts and dried fruits, yogurt, snacks, and fresh cheese;<br>PASTA AND MEAT: high on pasta and other grains, cooked tomatoes, red meat, white meat, olive oil, animal fats, other sauces, wine, beer, bread, offals, processed meat, and seasoned cheese; low on breakfast cereals and yogurt;<br>EGGS AND SWEETS: eggs, margarines, processed meat, sugar and sweets, vegetable oils, snacks, mayonnaises, butter, seasoned cheese, fresh cheese, pizza, canned fish, fruit juices, coffee, soft drinks, potatoes, white meat, red meat, animal fats, bread, and beer	ANOVA to provide adjusted estimates of mean factor scores across levels of nutrition knowledge;<br>Multiple linear regression models with each DP as dependent variable and nutrition knowledge (low, medium, and high) as independent variable;<br>Stratified analysis by education levels (≤8 ys, >8 ys)	ANOVA adjusted for age, sex, total EI;<br>Multiple linear regression adjusted for age, sex, total EI, BMI, PA, education level, income, SES, marital status, and smoking	OLIVE OIL AND VEGETABLES: in the overall analysis, adjusted mean (SD) factor scores reported in the text were -0.11 (0.95), -0.04 (0.82), 0.16 (0.96) for low, medium and high levels of nutrition knowledge, respectively; in stratified analysis by education, mean (SD) factor scores reported in the text were -0.09 (0.95), -0.07 (0.84), 0.16 (0.98) for low, medium and high levels of nutrition knowledge in high-educated subjects only, and -0.11 (0.96), 0.001 (0.80), 0.14 (0.92) for low, medium and high levels of nutrition knowledge in low-educated subjects only; higher mean factor scores were associated with higher nutrition knowledge levels overall (p-trend=0.001), in high-educated subjects only (p-trend=0.01), and borderline in low-educated subjects only (p-trend=0.05);<br>Other DPs did not provide additional evidence

Bonanni, 2013 (51)<br>Molise<br>Moli-sani<br>Good quality	PCFA<br>Standardization<br>EIG>1, Scree plot, and interpretability<br>Varimax rotation<br>|FL|≥0.15<br>Factorability checks NA<br>DP internal consistency NA<br>DP reproducibility (internal)	NA% (3)	OLIVE OIL AND VEGETABLES: olive oil, cooked and raw vegetables, legumes, soups, fruits, fish, potatoes, bouillon, white meat, crustaceans and molluscs, crisp bread and rusks, nuts and dried fruits, yogurt, snacks, and fresh cheese;<br>PASTA AND MEAT: high on pasta and other grains, cooked tomatoes, red meat, white meat, olive oil, animal fats, other sauces, wine, beer, bread, offals, processed meat, and seasoned cheese; low on breakfast cereals and yogurt;<br>EGGS AND SWEETS: eggs, margarines, processed meat, sugar and sweets, vegetable oils, snacks, mayonnaises, butter, seasoned cheese, fresh cheese, pizza, canned fish, fruit juices, coffee, soft drinks, potatoes, white meat, red meat, animal fats, bread, and beer	Multiple ANOVA with DPs as dependent variables and food labels reading status (yes/no) as independent variable	Adjusted for age, sex, total EI, SES, income, BMI, smoking, PA, and education level	OLIVE OIL AND VEGETABLES: mean factor scores were not equal across food labels reading categories (p-value<0.0001) with mean (SD) being 0.1 (0.8) in readers and -0.2 (0.8) in non-readers, as reported in the article;<br>PASTA AND MEAT: mean factor scores were not equal according to food labels reading categories (p-value=0.009) with mean (SD) being 0.01 (0.9) in readers and 0.2 (0.8) in non-readers, as reported in the article;<br>EGGS AND SWEETS: mean factor scores were not equal across food labels reading categories (p-value=0.02) with mean (SD) being 0.1 (0.8) in readers and 0.2 (0.9) in non- readers, as reported in the article

Bonaccio, 2013 (52) Molise Moli-sani Good quality	PCFA Standardization EIG>1, Scree plot, and interpretability Varimax rotation |FL|≥0.15 Factorability checks NA DP internal consistency NA DP reproducibility (internal)	NA% (3)	OLIVE OIL AND VEGETABLES: olive oil, cooked and raw vegetables, legumes, soups, fruits, fish, potatoes, bouillon, white meat, crustaceans and molluscs, crisp bread and rusks, nuts and dried fruits, yogurt, snacks, and fresh cheese; MEAT AND PASTA: high on pasta and other grains, cooked tomatoes, red meat, white meat, olive oil, animal fats, other sauces, wine, beer, bread, offals, processed meat, and seasoned cheese; low on breakfast cereals and yogurt; EGGS AND SWEETS: eggs, margarines, processed meat, sugar and sweets, vegetable oils, snacks, mayonnaises, butter, seasoned cheese, fresh cheese, pizza, canned fish, fruit juices, coffee, soft drinks, potatoes, white meat, red meat, animal fats, bread, and beer	Multiple linear regression models to assess association of each DP as independent variable (1 SD increase), and mental and physical health scores as continuous and dependent variables; Multiple logistic regression models on quartiles of factor scores for each DP as independent variable and extreme mental and physical health conditions (4th vs. 1st quartile-based score categories) as dependent variable; Stratified analysis by sex	Adjusted for age, sex, BMI, total EI, total PA, education, income, total SES, smoking, diabetes, hypertension and hypercholesterolemia, and additional adjustment for MUFAs, PUFAs, SFAs, FAC or dietary fiber in 5 separate models	OLIVE OIL AND VEGETABLES:1 SD increase in mental component score was directly related with factor scores (beta: 0.50, 95%CI: 0.34–0.65, p-value<0.0001); further adjustment for MUFAs, PUFAs or SFAs in 3 separate models did not materially change point estimates, CIs, and p-values, but results changed with further adjustment for FAC (beta: 0.19, 95%CI: -0.003–0.38, p-value=0.05) or dietary fiber (beta: 0.32, 95%CI: 0.15–0.50, p-value=0.0004) in 2 separate models; 1 SD increase in physical component score was directly associated with factor scores (beta: 0.15, 95%CI: 0.06–0.24, p-value=0.001); further adjustment for MUFAs, PUFAs, SFAs, FAC or dietary fiber in 5 separate models did not materially change point estimates, CIs, and p-values; ORs of being in the uppermost (4th) vs. 1st quartile of mental and physical component scores were 1.59 (95%CI: 1.39–1.83, p-trend<0.0001) and 1.22 (95%CI: 1.05–1.41, p-trend=0.01) for participants in the 4th vs. 1st quartile of factor scores; further adjustment for FAC or dietary fiber in 2 separate models did not materially change point estimates, CIs and p-trend values; MEAT AND PASTA: 1 SD increase in physical component score was inversely associated with factor scores (beta: -0.11, 95%CI: -0.20 to -0.02, p-value=0.02); further adjustment for MUFAs, PUFAs, SFAs, FAC or dietary fiber in 5 separate models did not materially change point estimates, and CIs; OR of being in the uppermost (4th) vs. 1st quartile of physical component scores was 0.86 (95%CI: 0.73–1.01, p-trend=0.02) for participants in the 4th vs. 1st quartile of factor scores; further adjustment for FAC or

						dietary fiber in 2 separate models did not materially change point estimates, CIs, and p-trend values;<br>EGGS AND SWEETS: 1 SD increase in mental component score was inversely associated with factor scores (beta=-0.33, 95%CI: -0.52 to -0.14, p-value=0.001); further adjustments for MUFAs, PUFAs or SFAs in 3 separate models did not materially change point estimates, CIs, and p-values but results changed with further adjustment for FAC (beta: -0.18, 95%CI: -0.39–0.01, p-value=0.06) or dietary fiber (beta: -0.16, 95%CI: -0.36–0.04, p-value=0.11) in 2 separate models; 1 SD increase in physical component score was not associated with factor scores; OR of being in the uppermost (4th) vs. 1st quartile of mental component score was 0.86 (95%CI: 0.73–1.01, p-trend=0.15) for participants in the 4th vs. 1st quartile of factor scores; further adjustment for FAC or dietary fiber in 2 separate models gave non-significant results; OR of being in the uppermost (4th) vs. 1st quartile of physical component score was 1.24 (95%CI: 1.05–1.47, p-trend=0.05) for participants in the 4th vs. 1st quartile of factor scores; further adjustment for FAC or dietary fiber in 2 separate models did not materially change point estimates, CIs, and p-trend values;<br>Stratified analyses did not provide additional evidence

Bonaccio, 2016 (53)<br>Molise<br>Moli-sani<br>Good quality	PCFA<br>Standardization<br>EIG>1, Scree plot, and interpretability<br>Varimax rotation<br>|FL|≥0.15<br>Factorability checks NA<br>DP internal consistency NA<br>DP reproducibility (internal)	13.5% (3)	OLIVE OIL AND VEGETABLES: olive oil, cooked and raw vegetables, legumes, soups, fruits, fish, potatoes, bouillon, white meat, crustaceans and molluscs, crisp bread and rusks, nuts and dried fruits, yogurt, snacks, and fresh cheese;<br>PASTA AND MEAT: high on pasta and other grains, cooked tomatoes, red meat, white meat, olive oil, animal fats, other sauces, wine, beer, bread, offals, processed meat, and seasoned cheese; low on breakfast cereals and yogurt;<br>EGGS AND SWEETS: eggs, margarines, processed meat, sugar and sweets, vegetable oils, snacks, mayonnaises, butter, seasoned cheese, fresh cheese, pizza, canned fish, fruit juices, coffee, soft drinks, potatoes, white meat, red meat, animal fats, bread, and beer	Cox proportional hazard model for overall mortality including factor scores for each DP (continuous variables, 1 SD increase) as independent variable	Adjusted for age, sex, education, EI, leisure-time PA, smoking, ys from diagnosis of diabetes, blood glucose levels, and hypercholesterolemia	No significant associations between overall mortality and DPs in the fully-adjusted model

Bonaccio, 2018 (54)<br>Molise<br>Moli-sani<br>Good quality	PCFA<br>Standardization<br>EIG>1, Scree plot, and interpretability<br>Varimax rotation<br>|FL|≥0.15<br>Factorability checks NA<br>DP internal consistency NA<br>DP reproducibility (internal)	6.6% (3)	OLIVE OIL AND VEGETABLES: olive oil, cooked and raw vegetables, legumes, soups, fruits, fish, potatoes, bouillon, white meat, crustaceans and molluscs, crisp bread and rusks, nuts and dried fruits, yogurt, snacks, and fresh cheese;<br>ANIMAL FATS AND MEAT: high on pasta and other grains, cooked tomatoes, red meat, white meat, olive oil, animal fats, other sauces, wine, beer, bread, offals, processed meat, and seasoned cheese; low on breakfast cereals and yogurt;<br>EGGS AND SWEETS: eggs, margarines, processed meat, sugar and sweets, vegetable oils, snacks, mayonnaises, butter, seasoned cheese, fresh cheese, pizza, canned fish, fruit juices, coffee, soft drinks, potatoes, white meat, red meat, animal fats, bread, and beer	Standardized multiple linear regression with psychological resilience as dependent variable and all DPs as independent variable; stratified analyses by age (≤65 ys, >65 ys), sex (Ms, Fs), education (up to lower secondary school, upper secondary school, post-secondary school), CVD status, cancer status, hypertension status, hypercholesterolemia status, and antidepressant use; further adjusted for the other two DPs	Adjusted for age, sex, EI, education, leisure-time PA, smoking habit, and cancer	OLIVE OIL AND VEGETABLES: psychological resilience scale increases of 1.184 (95%CI: 0.927–1.441, p-value<0.0001) for each 1 SD increase in factor score; point estimates and 95%CIs did not materially change after restricting the sample to a complete-case analysis of 10351 individuals (beta: 1.176, 95%CI: 0.914-1.439), nor to a subgroup of 3263 healthy subjects free from CVD, cancer, diabetes, hypercholesterolemia, hypertension, and depression (beta: 0.944, 95%CI: 0.500–1.388);<br>Stratified analyses revealed no material differences among strata except for sex (beta: 0.998, 95%CI: 0.643–1.354 among Ms; beta: 1.430, 95%CI: 1.053–1.807 among Fs), education (beta: 0.957, 95%CI: 0.516–1.399 among the "up to lower secondary school" group; beta: 1.358, 95%CI: 0.989–1.727 among the "upper secondary school"; beta: 1.264, 95%CI: 0.681–1.846 among the "post-secondary school" group) and cancer status (beta: 2.616, 95%CI: 1.100–4.133 among cancer subjects; beta: 1.154, 95%CI: 0.893–1.416 among non-cancer subjects) in the absence of any heterogeneity test;<br>Other DPs did not provide additional evidence in fully adjusted models

Pala, 2006 (55) Denmark, France, Germany, Greece, Netherlands, Spain, Sweden, UK, Italy (Varese, Turin, Florence, Naples, Ragusa) EPIC (EPIC-Elderly) Good quality	EFA Standardization EIG≥NA, Scree plot Varimax rotation |FL|≥0.30 Factorability checks NA DP internal consistency NA DP reproducibility NA	21% (4)	PRUDENT: other vegetables, legumes, cooked leafy vegetables, onions and garlic, cabbage, fish, crustaceans and molluscs, mushrooms, seed oils, cooked tomatoes, fresh fruit (non-citrus), and nuts and seeds; PASTA & MEAT: high on pasta and other grains, beef, other animal fats, cooked tomatoes, wine, bread, processed meat, and pork; low on yogurt; OLIVE OIL & SALAD: olive oil, raw tomatoes, raw leafy vegetables, root vegetables, soup, and chicken and turkey; SWEET & DAIRY: sugar and honey and jam, ice cream, chocolate-based confectionery, cakes and puddings, coffee, processed meat, eggs, milk, butter, cheese, and patisserie and biscuits	Crude means of factor scores and t-test testing the differences between factor scores of each DP by sex; separate analyses by sex: t-test testing the differences between sex-standardized crude means of factor scores (for each DP) by presence of high school education, hypertension status, hyperlipidemia status, whether on a diet, presence of recent modification of habitual diet; Separate analyses by sex: simple linear regression models inserting the category number into the model for p-trend evaluation, testing the presence of a	Not applicable	PRUDENT: different crude mean factor scores among Ms (-0.16) and Fs (0.06), p-value<0.001; different crude mean factor scores among Ms with (0.13) and without (-0.05) high school education (p-value=0.002) and Fs with (0.26) and without (-0.08) high school education (p-value<0.001); different crude mean factor scores among Fs with (-0.09) and without (0.02) recent modification of habitual diet (p-value=0.003); different crude mean factor scores among Fs with (0.07) and without (-0.04) hypertension (p-value=0.001), also when restricting the analysis to not on a diet Fs (p<0.001) with (0.11) and without (-0.03) hypertension; different crude mean factor scores among Fs with (0.04) and without (-0.02) hyperlipidemia (p-value=0.07), also when restricting the analysis to not on a diet Fs (p=0.02) with (0.01) and without (-0.02) hyperlipidemia; increasing crude mean factor scores were associated with higher BMI in Ms (p-trend=0.09) and Fs (p-trend<0.001), also when restricting the analysis to not on a diet Fs (p-trend<0.001); increasing crude mean factor scores were associated with higher WHR in Fs (p-trend<0.001), also when restricting the analysis to not on a diet Fs (p-trend<0.001); increasing crude mean factor scores were associated with higher PAL in Ms (p-trend<0.001) and lower PAL Fs (p-trend<0.001), also when restricting the analysis to not on a diet Ms (p-trend<0.001) and Fs (p-trend<0.001);<br>crude mean scores were not equal among all recruitment centers in Ms (0.02 in Florence, -0.30 in Varese, 0.76 in Ragusa, and -0.76 in Turin, p-value<0.001) and Fs (-0.19 in Florence, -0.45 in Varese, 0.32 in Ragusa, -

				trend in sex-standardized crude means of factor scores for each DP by BMI (<25, 25-29, 30-34, ≥35), WHR (in tertile-based on the distribution in men or women according to stratification), and PAL (in tertile-based on the distribution of the entire population); Separate analyses by sex: one-way ANOVA and F-test testing equality of sex-standardized crude means of factor scores for each DP among categories of recruitment center		0.25 in Turin, and 1.73 in Naples, p-value<0.001); PASTA & MEAT: different crude mean factor scores among Ms (0.76) and Fs (-0.29), p-value<0.001; different crude mean factor scores among Ms with (-0.21) and without (0.08) high school education (p-value<0.001) and Fs with (-0.06) and without (0.02) high school education (p-value=0.05); different crude mean factor scores among Ms with (-0.34) and without (0.06) recent modification of habitual diet (p-value<0.001) and Fs with (-0.33) and without (0.08) recent modification of habitual diet (p-value<0.001); different crude mean factor scores among not on a diet Fs with (0.12) and without (0.06) hypertension (p-value=0.08); different crude mean factor scores among Ms with (-0.11) and without (0.05) hyperlipidemia (p-value=0.005) and Fs with (-0.11) and without (0.06) hyperlipidemia (p-value<0.001), also when restricting the analysis to not on a diet Fs (p-value=0.001) with (-0.01) and without (0.08) hyperlipidemia; increasing crude mean factor scores were associated with higher BMI in Ms (p-trend=0.001) and Fs (p-trend<0.001), also when restricting the analysis to not on a diet Ms (p-trend=0.004) and Fs (p-trend<0.001); increasing crude mean factor scores were associated with higher WHR in Ms (p-trend<0.001) and Fs (p-trend<0.001), also when restricting the analysis to not on a diet Ms (p-trend=0.001) and Fs (p-trend<0.001); increasing crude mean factor scores were associated with higher PAL (p-trend=0.003), also when restricting the analysis to not on a diet Ms (p-trend=0.003); crude mean scores were not equal among all recruitment centers in Ms

						(0.16 in Florence, 0.14 in Varese, -0.22 in Ragusa, and -0.13 in Turin; p-value<0.001) and Fs (0.11 in Florence, -0.13 in Varese, -0.13 in Ragusa, -0.32 in Turin, and 0.37 in Naples, p-value<0.001);<br>OLIVE OIL & SALAD: different crude mean factor scores among Ms (0.16) and Fs (-0.06), p-value<0.001; different crude mean factor scores among Ms with (0.26) and without (-0.05) recent modification of habitual diet (p-value<0.001) and Fs with (0.36) and without (-0.09) recent modification of habitual diet (p-value<0.001); different crude mean factor scores among Ms with (0.07) and without (-0.03) hyperlipidemia (p-value=0.06) and Fs with (0.09) and without (-0.05) hyperlipidemia (p-value<0.001), also when restricting the analysis to not on a diet Fs (p-value=0.001) with (-0.02) and without (-0.06) hyperlipidemia; increasing crude mean factor scores were associated with higher BMI in Ms (p-trend=0.014); decreasing crude mean factor scores were associated with higher WHR in Ms (p-trend=0.015) and Fs (p-trend<0.001), also when restricting the analysis to not on a diet Fs (p-trend<0.001); increasing crude mean factor scores were associated with higher PAL in Fs (p-trend<0.001), also when restricting the analysis to not on a diet Fs (p-trend<0.001); crude mean scores were not equal among all recruitment centers in Ms (0.09 in Florence, -0.05 in Varese, -0.65 in Ragusa, and 0.23 in Turin, p-value<0.001) and Fs (0.15 in Florence, 0.05 in Varese, -0.24 in Ragusa, 0.42 in Turin, and -0.81 in Naples, p-value<0.001);<br>SWEET & DAIRY: different crude mean factor scores among Ms (-0.04) and Fs

						(0.02), p-value=0.046; different crude mean factor scores among Fs with (0.06) and without (-0.02) high school education (p-value=0.04); different crude mean factor scores among Ms with (-0.23) and without (0.04) recent modification of habitual diet (p-value<0.001) and Fs with (-0.19) and without (0.04) recent modification of habitual diet (p-value<0.001); different crude mean factor scores among Fs with (-0.04) and without (0.02) hypertension (p-value=0.06); different crude mean factor scores among Ms with (-0.19) and without (0.09) hyperlipidemia (p-value<0.001) and Fs with (-0.11) and without (0.06) hyperlipidemia (p-value<0.001), also when restricting the analysis to not on a diet Ms (p-trend<0.001) with (-0.16) and without (0.12) hyperlipidemia and Fs (p-trend<0.001) with (-0.16) and without (0.12) hyperlipidemia; decreasing crude mean factor scores were associated with higher BMI in Fs (p-trend=0.003), also when restricting the analysis to not on a diet Fs (p-trend<0.001); decreasing crude mean factor scores were associated with higher WHR in Fs (p-trend<0.001), also when restricting the analysis to not on a diet Fs (p-trend<0.001); increasing crude mean factor scores were associated with higher PAL in Fs (p-trend<0.001), also when restricting the analysis to not on a diet Fs (p-trend<0.001); crude mean scores were not equal among all recruitment centers in Ms (0.05 in Florence, 0.28 in Varese, -0.09 in Ragusa, and -0.22 in Turin, p-value<0.001) and Fs (0.05 in Florence, 0.23 in Varese, -0.25 in Ragusa, -0.11 in Turin, and -0.50 in Naples, p-value<0.001)

Masala, 2007 (56) Denmark, France, Germany, Greece, Netherlands, Spain, Sweden, UK, Italy (Varese, Turin, Florence, Naples, Ragusa) EPIC (EPIC-Elderly) Very good quality	EFA Standardization NA EIG≥NA, Scree plot Varimax rotation |FL|≥0.30 Factorability checks NA DP internal consistency NA DP reproducibility NA	21% (4)	PRUDENT: other vegetables, legumes, cooked leafy vegetables, onions and garlic, cabbage, fish, crustaceans and molluscs, mushrooms, seed oil, fresh fruit (non-citrus), cooked tomatoes, and nuts and seeds;<br>PASTA & MEAT: high on pasta and other grains, beef, other animal fats, cooked tomatoes, wine, white bread, processed meat, and pork; low on yogurt;<br>OLIVE OIL & SALAD: olive oil, raw tomatoes, raw leafy vegetables, root vegetables, soup, and chicken and turkey;<br>SWEET & DAIRY: sugar and honey and jam, ice cream, chocolate-based confectionery, cakes and puddings, coffee, processed meat, eggs, milk, butter, cheese, and patisserie and biscuits	Multiple Cox proportional hazard regression models to estimate overall mortality (HR) according to sex-specific quartiles of factor scores of each DP as independent variable in separate models; stratified analysis by recruitment center due to center-specific DPs and additional stratified analyses by sex and geographic area in center-specific models	Adjusted for sex, age, log-transformed EI, BMI, waist, smoking status, ys of education, civil status, hypertension status at enrolment, and PAL	OLIVE OIL & SALAD: being in the 4th vs. 1st quartile of factor scores protected against overall mortality (HR: 0.50, 95%CI: 0.29–0.86, p-trend=0.02); stratified analyses by sex and geographic area (northern vs. central-southern centers) did not provide significant results;<br>Other DPs did not additional evidence
Jannasch, 2019 (57) Italy, France,	Separate PCFAs on each country Standardization	18.3% (2)	PC1: leafy vegetables, fruiting vegetables, cabbage, other vegetables, legumes,	Prentice-weighted multiple Cox proportional	Adjusted for sex, PA, education level, smoking status,	PC1: increase in factor score was borderline associated with an increased hazard of type 2 diabetes in Italy (HR: 1.10, 95%CI: 0.98–1.23, p-value=0.1);

Spain, UK, Netherlands, Germany, Sweden, Denmark EPIC-InterAct Good quality	n EIG>1, Scree plot, and interpretability Varimax rotation Simplified sum score with different cut-offs for FL values, but final cut-off equal to |0.4| Factorability checks NA DP internal consistency NA DP reproducibility (internal and cross-study)		fish, and vegetable oils; PC2: pasta and rice, red meat, processed meat, other fats, and sugar	hazard regression models, investigating the association between DPs and hazard of type 2 diabetes incidence; Stratified analysis by country and center, if present, due to country/center-specific DPs; additional stratified analysis by integers of age; Cross-study reproducibility of DPs: meta-analysis of diabetes-associated country-specific DPs using simplified or replicated scores	total EI, BMI, and waist circumference	the other DP did not provide additional evidence for Italy; data not shown on stratified analyses by integers of age; Cross-study reproducibility of DPs: among the country-specific DPs associated with diabetes risk, the positive association of the UK-Norfolk DP could potentially be replicated across other countries in the EPIC-InterAct study, but not those form Spain and France
Balder, 2003 (58) Netherlands, Sweden, Finland, and Italy DIETSCAN Project	Separate PCFAs on each of the 4 studies (but NLSC separate analyses for Ms and Fs)	ORDET: 28.5% (4)	(SALAD) VEGETABLES: raw leafy vegetables, dressings, tomatoes, oil, and carrots; PORK, PROCESSED MEAT, POTATOES: butter, non-fermented	Not applicable	Not applicable	Not applicable Cross-study reproducibility: From visual inspection of PCFA loadings across solutions, (SALAD) VEGETABLES: factors were qualitatively similar across studies and between Ms and Fs; PORK, PROCESSED MEAT, POTATOES:

(NLSC, SMC, ATBC, ORDET)<br>Poor quality	Standardization<br>EIG>1, Scree plot, and interpretability<br>Varimax rotation<br>|FL|≥0.35<br>Factorability checks NA<br>DP internal consistency NA<br>DP reproducibility (internal and cross-study)		whole milk, pasta, beef, potatoes, processed meat, cakes, and eggs;<br>COOKED VEGETABLES: legumes, cabbages, cooked leafy vegetables, fish, carrots, rice, potatoes, and poultry;<br>ALCOHOL: high on wine and spirits; low on coffee (with milk), non-fermented lowfat milk, cakes, and other fruits			factors were qualitatively similar across studies and between Ms and Fs, but somewhat less consistently than (SALAD) VEGETABLES;<br>COOKED VEGETABLES: found for ORDET and NLCS Ms;<br>ALCOHOL: found for ORDET, ATBC, and SMC
Männistö, 2005 (59)<br>Netherlands, Sweden, and Italy<br>DIETSCAN Project (NLSC, SMC, ATBC, ORDET)<br>Good quality	Separate PCFAs on each of the 3 studies<br>Standardization<br>EIG>1, Scree plot, and interpretability<br>Varimax rotation<br>|FL|≥0.35<br>Factorability checks NA<br>DP internal consistency NA<br>DP reproducibility NA	ORDET: 28.5% (2)	VEGETABLES (VEG): raw leafy vegetables, tomatoes, dressings, oil, and carrots;<br>PORK, PROCESSED MEAT, POTATOES (PPP): butter, pasta, potatoes, beef and veal, and processed meat;<br>Plus 2 additional DPs for ORDET (presented in Balder et al.) but not common to other cohorts and therefore not considered here	ORDET: multiple Cox proportional hazard regression models investigating the association between breast cancer incidence and factor scores of each DP in continuum and in quartiles, in separate models;<br>Stratified analysis by country due to region-specific DPs and	Adjusted for age, BMI, height, education, smoking status, family history of breast cancer, OC and HRT use, alcohol intake, and EI	ORDET: no significant associations between any of the two DPs and breast cancer incidence was identified; stratified analyses by menopausal status did not provide any additional evidence

				additional stratified analysis by menopausal status in country-specific models		
Sieri, 2004 (60)<br>Varese (Lombardy)<br>ORDET<br>Very good quality	EFA<br>Standardization NA<br>EIG>NA,<br>Scree plot<br>Varimax rotation<br>|FL|>0.25<br>Factorability checks NA<br>DP internal consistency NA<br>DP reproducibility NA	30%<br>(4)	SALAD VEGETABLES: raw and cooked leafy vegetables, mixed vegetables in salad, raw tomatoes, raw carrots, olive oil and other fruiting vegetables;<br>WESTERN: butter, potatoes, other pasta, processed meat, veal, eggs, cakes, beef, seed oils, offal, pork, and cheese;<br>CANTEEN: pasta, cooked tomatoes, olive oil, pulses, other fruiting vegetables, veal, bread and wine;<br>PRUDENT: high on cooked carrots, cooked leafy vegetables, rice, fish, other fruiting vegetables, pulses, poultry, raw carrots, potatoes, yogurt, and	Multiple Cox proportional hazard regression model investigating the association between breast cancer incidence and tertiles of factors scores for all DPs, using 1st tertile as reference;<br>Stratified analysis by BMI (<25 kg/m^2, ≥ 25 kg/m^2)	Adjusted for EI, age, ys of education, parity, height, age at menarche, smoking, and menopausal status	SALAD VEGETABLES: being in the 3rd vs. 1st tertile of factor scores was associated with a lower risk of breast cancer (RR: 0.66, 95%CI: 0.47–0.95, p-trend=0.016); stratified analyses by BMI: in BMI<25 women RR=0.39, 95%CI: 0.22–0.69, p-trend=0.001; in BMI≥25 women RR did not give significant results;<br>Other DPs did not provide additional evidence

			olive oil; low on wine and spirits			
Sant, 2007 (61) Varese (Lombardy) ORDET Very good quality	EFA Standardization EIG>NA, Scree plot Varimax rotation |FL|>0.25 Factorability checks NA DP internal consistency NA DP reproducibility NA	30% (4)	SALAD VEGETABLES: raw and cooked leafy vegetables, mixed vegetables in salad, raw tomatoes, raw carrots, olive oil and other fruiting vegetables; WESTERN: butter, potatoes, other pasta, processed meat, veal, eggs, cakes, beef, seed oils, offal, pork, and cheese; CANTEEN: pasta, cooked tomatoes, olive oil, pulses, other fruiting vegetables, veal, bread and wine; PRUDENT: high on cooked carrots, cooked leafy vegetables, rice, fish, other fruiting vegetables, pulses, poultry, raw carrots, potatoes, yogurt, and olive oil; low on wine and spirits	Multinomial multiple logistic regression model to assess risk of HER2+ and HER2- breast cancer (vs. non-cases) according to tertiles of factor scores for all DPs in the same model (1st tertile as reference); RR heterogeneity between HER2+ and HER2- assessed by Wald test	Adjusted for total EI, age, ys of education, parity, height, weight, age at menarche, smoking, and menopausal status	SALAD VEGETABLES: being in the 3rd vs. 1st tertile of factor scores was associated with a reduced risk of HER2+ breast cancer (RR: 0.25, 95%CI: 0.10–0.64, p-trend=0.001), much stronger than risk of HER2- breast cancer (RR: 0.71, 95%CI: 0.48–1.03, p-trend=0.072) (p-heterogeneity=0.039); Other DPs did not provide additional evidence

Menotti, 2012 (62) Italian Rural Areas of Seven Countries Study of Cardiovascular Disease Seven Countries Study Very good quality	PCFA Standardization NA Adjustment by weight EIG>1, Scree plot Varimax rotation |FL|≥0.25 Factorability checks NA DP internal consistency NA DP reproducibility (internal) with PCA	≥82%* (3)	FACTOR 1: sugar, milk, meat, fruit, pastries, and cheese; FACTOR 2: bread, cereals, vegetables, fish, potatoes, and oils; FACTOR 3: eggs and alcoholic beverages	Multiple Cox proportional hazard models assessing HRs of CHD-specific incidence at 20-y follow-up, overall mortality and mortality from CHD, CVD or cancer at 40-y follow-up in separate models, according to standardized factor scores for all DPs as independent variables	Adjusted for age, BMI, smoking status, SBP, and serum cholesterol	FACTOR 1: 1 SD increase in factor scores was associated with borderline reduced CHD mortality (HR: 0.87, 95%CI: 0.76–1.01) and cancer mortality (HR 0.91, 95%CI: 0.81–1.01) and with borderline increased CVD mortality (HR: 1.07, 95%CI: 0.98–1.18), at 40-y follow-up; FACTOR 2: 1 SD increase in factor scores was associated with reduced CHD incidence (HR: 0.88, 95%CI: 0.73–0.96) at 20-y follow-up, CHD mortality (HR 0.79, 95%CI: 0.66–0.95), CVD mortality (HR: 0.87, 95%CI: 0.78–0.96), cancer mortality (HR: 0.84, 95%CI: 0.74–0.96), and overall mortality (HR:0.89, 95%CI: 0.83–0.96), at 40-y follow-up; FACTOR 3: 1 SD increase in factor scores was associated with borderline increased CHD mortality (HR: 1.17, 95%CI, 0.97–1.40), with decreased cancer mortality (HR:0.86, 95%CI: 0.77–0.97) and overall mortality (HR: 0.93, 95%CI: 0.97–1.00, as reported in the article), at 40-y follow-up
Menotti, 2018 (63) Italian Rural Areas of Seven Countries Study of Cardiovascular Disease Seven Countries Study Very good quality	PCA and EFA Standardization NA Energy adjustment (density method) EIG>1, Scree plot Varimax rotation |FL|≥0.30 Factorability checks NA DP internal consistency	≥82%* (3)	FA2 (EFA-based FACTOR2 from Menotti 2012): bread, cereals, vegetables, fish, potatoes, and oils; PCA2 (PCA-based COMPONENT2 from Menotti 2012): bread, cereals, vegetables, fish, potatoes, and oils; Plus 2 additional factors and 2 additional principal components not further investigated due to the lack of	Multiple Cox proportional hazard models assessing HRs of CHD mortality at 40-y follow-up, according to tertiles of factor scores of FA2 and PC2 as separate independent variables; Kaplan-Meier curves and log-rank test to	Adjusted for age, cigarettes smoking, SBP, serum cholesterol, BMI, and PA	FA2: being in the 3rd vs. 1st tertile of factor scores was associated with reduced hazard of CHD mortality (HR: 0.65, 95%CI: 0.45–0.94); PC2: being in the 3rd vs. 1st tertile of factor scores was associated with reduced hazard of CHD mortality (HR: 0.53, 95%CI: 0.37–0.77)

	NA<br>DP reproducibility (internal)		association with CHD mortality	(unproperly due to competing risks) assess difference in CHD survival according to tertiles of factor scores		
Maugeri, 2019 (64)<br>Mamma & Bambino Catania (Sicily)<br>Fair quality	PCFA<br>Standardization<br>Energy adjustment (residual method)<br>EIG>2, Scree plot, and interpretability<br>Varimax rotation<br>|FL|≥0.25<br>Factorability checks NA<br>DP internal consistency NA<br>DP reproducibility NA	15.6% (2)	PRUDENT: potatoes, raw and cooked vegetables, legumes, rice, and soup;<br>WESTERN: red meat, fries, dipping sauces, salty snacks, and alcoholic drinks	Multiple logistic regression models assessing ORs for being in the 3rd tertile of each factor score vs. being in 1st or 2nd tertile combined, according to age, pre-gestational BMI, education level, employment status, smoking status, use of folic acid supplements, use of multivitamin supplements	(Mutually) adjusted for age, education level, employment status, smoking, pre-gestational BMI, use of folic acid supplements and use of multivitamin and/or multi-mineral supplements	PRUDENT: being in the 3rd factor score tertile was inversely associated with increases in pre-gestational BMI (continuous) (OR 0.920, 95%CI: 0.865–0.978; p-value=0.007);<br>WESTERN: being in the 3rd factor score tertile was inversely associated with increases in age (continuous) (OR 0.885, 95%CI: 0.829–0.945; p-value=0.001) and directly associated with medium-low education level (OR 1.617, 95%CI: 1.006–3.374; p-value=0.047) and current smoking status (OR 1.812, 95%CI: 1.004–3.269; p-value=0.048);<br>Other sociodemographic characteristics did not provide additional evidence for the previous DPs

Maugeri, 2019 (65)<br>Mamma & Bambino Catania (Sicily)<br>Fair quality	PCFA<br>Standardization<br>Energy adjustment (residual method)<br>EIG>2, Scree plot, and interpretability<br>Varimax rotation<br>|FL|≥0.20<br>Factorability checks NA<br>DP internal consistency NA<br>DP reproducibility (internal)	15.55% (2)	PRUDENT: potatoes, cooked vegetables, legumes, pizza, and soup;<br>WESTERN: red meat, fries, dipping sauces, salty snacks, and alcoholic drinks	Multiple linear regression models with BMI or GWG as dependent variables in separate models, according to tertiles of each DP as independent variable; the interaction between gestational age at recruitment and adherence to DP on pre-gestational BMI was tested and found non-significant; Stratified analysis by pre-gestational BMI categories (underweight, normal weight, overweight, and obese) for the model with GWG	Pre-gestational BMI model adjusted for age, education level, employment status, smoking, total EI, gestational age at recruitment; GWG model adjusted for age, length of gestation, birth weight, education level, employment status, smoking, parity, newborn sex, total EI	WESTERN: no association with pre-gestational BMI in the overall model; no association with GWG in the overall model, but p-trend=0.013; being in the 3rd vs. 1st tertile of factor scores was positively associated with GWG in pre-pregnancy obese women (beta=13.701, SE=0.887, p-value=0.041, p-trend=0.005);<br>PRUDENT: higher factor scores were associated with decreased pre-gestational BMI (beta=-0.631, SE=0.318, p-value=0.038) for a 1 SD increase; being in the 3rd vs. 1st tertile of factor scores was associated with decreased pre-gestational BMI (beta=-1.347, SE=0.598, p-value=0.024); no association with GWG in the overall model; no association with GWG in underweight and normal-weight women; being in the 3rd vs. 1st tertile of factor score was inversely associated with GWG in pre-pregnancy over-weight women (beta=-9.736, SE=4.302, p-value=0.037, p-trend=0.016) and in obese women with borderline significance (beta=-10.730, SE=4.156, p-value=0.061, p-trend=0.031) in the absence of any heterogeneity test

Magnano San Lio, 2022 (66) Catania (Sicily) Good quality	PCFA on the overall sample Standardization Energy adjustment, NA method EIG>2, Scree plot, and interpretability Varimax rotation |FL|≥0.4 Factorability checks NA DP internal consistency NA DP reproducibility NA	15.6% (2)	PRUDENT: cooked and raw vegetables, legumes, fruits, fish, and soup; WESTERN: white bread, vegetable oil, fries, salty snacks, dipping sauces, and sweets	Multiple logistic regression models assessing for each DP the ORs of being in the 3rd vs.1st tertile (only) of factor scores according to the cohort of enrollment	Adjusted for maternal age, gestational age at recruitment, education level, employment status, pre-gestational BMI, and smoking status	PRUDENT: mothers enrolled during COVID-19 pandemic were less likely to adhere to this DP than those enrolled before: OR of being in the uppermost (3rd) vs. 1st tertile of adherence was 0.26 (95%CI: 0.15–0.43, p<0.001) for participants of Mamma & Bambino cohort compared to MAMI-MED cohort; Adjusted analysis on WESTERN DP did not provide additional evidence
Ojeda-Granados, 2022 (67) Catania (Sicily), Guadalajara (Mexico) Fair quality	Separate PCFAs on each country Standardization Energy adjustment (residual method) EIG>2, Scree plot, and interpretability Varimax rotation |FL|≥0.2 Factorability checks NA DP internal	15.3% (2)	LEGUMES, VEGETABLES AND FISH (DP1): legumes, cooked and raw vegetables, vegetable soup, potatoes, and fish; SNACK FOODS, PROCESSED MEATS AND OILS (DP2): chips, dipping sauces, snacks, processed meat, vegetable oils, red meat, sugar and sweets, and fruit juice	One-way ANOVA (followed by post-hoc comparisons) or Kruskal-Wallis test (followed by Mann-Whitney test as reported in the text) for age, weight, BMI, body fat, total EI, and various macro- and micro-nutrients as dependent variables, or chi-	Not applicable	Distribution of age (p=0.001), weight (p=0.065), PUFAs (p=0.001), folates (p<0.001), vitamin A (p<0.001), vitamin C (p<0.001), vitamin D (p<0.001), thiamin (p=0.054), pyridoxine (p<0.001), calcium (p=0.007), iron (p<0.001) magnesium (p<0.001), and zinc (p=0.039) according to tertile-based categories of DP adherence was not similar across the 5 combined categories of tertiles of the 2 DPs; EXCLUSIVELY LEGUMES, VEGETABLES AND FISH (DP1): age was higher compared to exclusively DP2 (p=0.006) and to preferably DP2 (p<0.001); percentage of PUFAs was lower compared to preferably DP2, and exclusively DP2 (p<0.032); folates (p<0.001) and vitamin A (p<0.012) were higher compared to all the other categories;

	consistency<br>NA<br>DP reproducibility<br>NA			square test for BMI (in categories) and combined tertile-based categories of adherence to the two DPs (exclusively adherent for either DP, preferably adherent for either DP, or no preference)		vitamin C was higher compared to preferably DP2, exclusively DP2, and no preference (p<0.025); vitamin D (p<0.004), pyridoxine (p<0.002), magnesium (p<0.001), and zinc (p<0.015) were higher compared to all the other categories; calcium was higher compared to exclusively DP2 (p<0.016); iron was higher compared to preferably DP2 and exclusively DP2 (p<0.003);<br>PREFERABLY LEGUMES, VEGETABLES AND FISH (DP1): age was higher compared to exclusively DP2 (p=0.046) and to preferably DP2 (p=0.002); percentage of PUFAs was lower compared to preferably DP2, and exclusively DP2 (p<0.032); folates (p<0.001) and vitamin A (p<0.012) were higher compared to all the other categories; vitamin C was higher compared to preferably DP2, exclusively DP2, and no preference (p<0.025); vitamin D was higher compared to preferably DP2 (p=0.007); pyridoxine was higher compared to preferably DP2 (p=0.028); iron was higher compared to preferably DP2 and exclusively DP2 (p<0.003); calcium was higher compared to exclusively DP2 (p<0.016); magnesium was higher compared to preferably DP2 and exclusively DP2 (p=0.001);<br>NO PREFERENCE: age was higher compared to preferably DP2 (p=0.015)

Barchitta, 2018 (68) Catania (Sicily) Good quality	PCFA Standardization Energy adjustment (residual method) EIG>2, Scree plot, and interpretability Varimax rotation |FL|≥0.2 Factorability checks NA DP internal consistency NA DP reproducibility (internal)	14.31% (2)	PRUDENT: legumes, vegetable soups, potatoes, cooked and raw vegetables, and olive oil; WESTERN: high on chips, snacks, dipping sauces, plant oils, processed and red meats; low on olive oil	Multiple logistic regression models assessing ORs of hrHPV infection (among women with normal epithelium) and OR of CIN2+, according to quartiles of factor scores or one unit increase of factor scores (independent variable) for identified DPs	hrHPV infection model: adjusted for age, BMI, smoking status, and parity; CIN2+ model: adjusted for age and hrHPV status	WESTERN: increasing factor scores were borderline significantly associated with increased risk of hrHPV infection (OR: 1.39, 95%CI: 0.97–1.99, p-value=0.069, for one unit increase); PRUDENT: increasing quartile-based categories of factor scores were borderline significantly associated with a reduced risk of CIN2+ (p-trend=0.076)
Barchitta, 2019 (69) Catania (Sicily) Good quality	PCFA Standardization Energy adjustment (residual method) EIG>2, Scree plot, and interpretability Varimax rotation |FL|≥0.3 Factorability checks NA DP internal consistency NA	17.2% (2)	PRUDENT: potatoes, cooked and raw vegetables, legumes, soup, and fish; WESTERN: high on canned fish, vegetable oil, processed meat, salty snacks, alcoholic drinks, and dipping sauces; low on fruits	Multiple linear regression models assessing the association between tertiles of factor scores and log-transformed leucocyte LINE-1 methylation (4 dependent variables CpG site 1, 2, 3, and average) for each DP; Multiple linear regression	Adjusted for age, education level, employment status, smoking status, use of folic acid supplement, total EI, and BMI	PRUDENT: mean leucocyte LINE-1 methylation levels were higher in the 3rd vs.1st tertile of factor scores at CpG site 1 (beta=0.009, SE=0.003, p=0.001, p-trend<0.001), CpG site 2 (beta=0.030, SE=0.005, p<0.001, p-trend<0.001), and CpG site 3 (beta=0.034, SE=0.003, p<0.001, p-trend<0.001); average leucocyte LINE-1 methylation levels were higher in the 3rd vs. 1st tertile of factor scores (beta=0.022, SE=0.003, p<0.001, p-trend<0.001); women who exclusively adhered to this DP had a higher average leucocyte LINE-1 methylation level than those who adhered to the no preference category (beta=0.013, SE=0.004, p=0.002), to the preferably adherent to WESTERN DP category (beta=0.023, SE=0.004, p<0.001), and to the exclusively

	DP reproducibility NA			models assessing the association between combined tertile-based categories of adherence to DPs (exclusively adherent, preferably adherent, with no preference) and average leucocyte LINE-1 methylation level for each DP		adherent to WESTERN DP category (beta=0.030, SE=0.004, p<0.001);<br>Other DPs did not provide additional evidence
Barchitta, 2019 (70)<br>Eastern Sicily<br>Fair quality	PCFA<br>Standardization<br>Energy adjustment (residual method)<br>EIG>2, Scree plot, and interpretability<br>Varimax rotation<br>|FL|≥0.2<br>Factorability checks NA<br>DP internal consistency NA<br>DP reproducibility NA	26.8% (3)	PRUDENT: potatoes, cooked vegetables, legumes, fruits, nuts, yogurt, offals, shellfish, and tea;<br>WESTERN: white bread, red and processed meat, shellfish, vegetable oil, dipping sauces, and fries;<br>ENERGY DENSE: yogurt, butter and margarine, sweets and refined sugar, dipping sauces, pizza, and fries	Spearman correlation coefficient and hypothesis test to assess correlation between factor scores of each DP and school marks (Italian, English, History, Science, PE, Mathematics, Comportment, and GPA)	Not applicable	PRUDENT: adherence was positively associated with marks in Mathematics (r=0.150, p-value<0.05);<br>WESTERN: adherence was negatively associated with marks in English (r=-0.217, p-value<0.05), History (r=-0.174 ,p-value<0.05), Science (r=-0.158, p-value<0.05), PE (r=-0.221, p-value<0.05), Comportment (r=-0.168, p-value<0.05), and GPA (r=-0.220, p-value <0.05);<br>ENERGY DENSE: adherence was negatively associated with marks in Italian (r=-0.165, p-value<0.05)

Fernández-Alvira, 2014 (71) Italy, Estonia, Cyprus, Belgium, Sweden, Hungary, Germany, and Spain IDEFICS Good quality	Separate PCFA by center Standardization NA EIG>1, Scree plot Varimax rotation |FL|≥0.3 Factorability checks NA DP internal consistency NA DP reproducibility (cross-study)	20.5% (3)	PROCESSED: crisps, corn crisps and popcorn, ketchup, chocolate and candy bars, mayonnaise and mayonnaise-based products, and sweetened drinks; HEALTHY: raw vegetables, cooked vegetables and beans, fresh fruits without added sugar, fresh or frozen fish (not fried), and fresh meat (not fried); SPREADS: reduced-fat products on bread, butter and/or margarine on bread, jam and honey, and chocolate or nut-based spread	Multiple linear regression models performed to assess the impact of SES (independent variable) on children's DP scores for one DP at a time (dependent variable); Stratified analysis by country due to country-specific DPs	Adjusted for sex, age, and migrant background	PROCESSED: increases in children factor scores were associated with reductions of the family SES indicator in the Italian cohort (beta=-0.063, 95%CI: -0.077 to -0.049, p-value<0.001); Other DPs did not provide additional evidence in the Italian cohort Cross-study reproducibility: PROCESSED: identified in all the 8 regions; HEALTHY: identified in 7 investigated regions, but not in Cyprus; SPREADS: identified in Italy only
Naska, 2006 (72) Belgium, France, Finland, Germany, Greece, Italy, Norway, Portugal, Spain, UK DAFNE Fair quality	Separate PCAs by country on daily individual food availability defined as recorded food quantities divided by the corresponding household values (defined as age and sex specific	PC1: 15-20%; PC2: 6-8% (2)	WIDE RANGE: high on fruits, vegetables, cereals, meat, fish, and dairy products; BEVERAGE AND CONVENIENCE: high on beverages (alcoholic and nonalcoholic) and ready-to-eat dishes; low on plant foods and elaborate-to-cook dishes	Separate multiple linear regression models by country including factor scores of each DP as dependent variables and socio-demographic characteristics as independent variables; the analysis was	(Mutually) adjusted for education level, locality, occupation, and household composition	WIDE RANGE: compared to elementary education, secondary (beta: -0.22, 95%CI: -0.30 to -0.14) or higher (beta: -0.40, 95%CI: -0.55 to -0.25) education was inversely related to factor scores; compared to rural locality, living in semi-urban (beta: 0.14, 95%CI: 0.05–0.24) or urban localities (beta: 0.34, 95%CI: 0.25–0.43) was directly related to factor scores; compared to manual occupation of the household head, retirement (beta: 0.49, 95%CI: 0.38–0.60) or other occupation (beta: 0.41, 95%CI: 0.29–0.53) were directly related to factor scores; compared to a single adult household, being in a lone parent household (beta: 0.29, 95%CI: 0.02–0.57), in a single elderly

	consumption units calculated on the basis of the respective average energy requirements using energy requirements of males aged 18-29 ys as the reference unit)<br>Standardization<br>Log-transformation of individual food availability relative to the overall average DAFNE food availability (calculated for each FG as unweighted arithmetic mean of the country-specific mean availability values)<br>EIG>1, and interpretability<br>Rotation NA<br>|FL|≥0.2			based on a subset of households whose composition fits specific pre-defined categories (Italy: 16% households lost)		household (beta: 0.33, 95%CI: 0.18–0.47), or in a 2 elderly members household (beta: 0.33, 95%CI: 0.17–0.50) was directly related to factor scores;<br>BEVERAGE AND CONVENIENCE: compared to elementary education, secondary (beta: 0.21, 95%CI: 0.16–0.25) or higher (beta: 0.24, 95%CI: 0.15–0.32) education was directly related to factor scores; compared to rural localities, living in urban localities was inversely related to factor scores (beta: -0.17, 95%CI: -0.22 to -0.12); compared to manual occupation of household head, non-manual occupation was directly related to factor scores (beta: 0.09, 95%CI: 0.04–0.14), while retirement (beta: -0.16, 95%CI: -0.23 to -0.16) or other occupation (beta: -0.25, 95%CI: -0.32 to -0.19) were inversely related to factor scores; compared to a single adult household, being in a 2 members adult household (beta: 0.22, 95%CI: 0.15–0.29), in a lone parent household (beta: 0.41, 95%CI: 0.26–0.56), or in an adults and children household (beta: 0.46, 95%CI: 0.40–0.52) was directly related to factor scores, while being in a single elderly household (beta: -0.26, 95%CI: -0.34 to -0.18) or in a 2 members elderly household (beta: -0.17, 95%CI: -0.27 to -0.08) was inversely related to factor scores<br>Cross-study reproducibility:<br>WIDE RANGE: remarkably similar in all countries;<br>BEVERAGE AND CONVENIENCE: slightly more varied among the 10 countries

	Factorability checks NA<br>DP internal consistency NA<br>DP reproducibility (cross-study)					
Bravi, 2021 (73)<br>Turin (Piemonte), Florence (Tuscany), Rome (Lazio), San Giovanni Rotondo (Apulia), Palermo (Sicily)<br>MEDIDIET<br>Fair quality	PCFA<br>Standardization<br>EIG>1, Scree plot, and interpretability<br>Varimax rotation<br>|FL|≥0.63<br>Factorability checks<br>DP internal consistency<br>DP reproducibility (internal)	80.57% (5)	VITAMINS, MINERALS AND FIBERS: fiber, potassium, iron, folate, vitamin C, vitamin E, and beta-carotene equivalents;<br>PROTEINS AND FATTY ACIDS WITH LEGS: animal protein, SFAs, cholesterol, calcium, phosphorus, zinc, and riboflavin;<br>FATTY ACIDS WITH FINS: EPA, DHA, DPA, and vitamin D;<br>FATTY ACIDS WITH LEAVES: MUFAs, LA, ALA, vitamin E, and lycopene;<br>STARCH AND VEGETABLE PROTEINS: starch, vegetable protein, and sodium	One-way ANOVA to assess the presence of differences in means of foremilk characteristics (protein, lactose, fat, fat composition and energy density) according to quartiles of factor scores of each maternal DP	Not applicable	VITAMINS, MINERALS AND FIBERS: mean foremilk content in omega-3 (p-value= 0.0029), ALA (p-value=0.0507), EPA (p-value=0.0195), DHA (p-value=0.0093), and DPA (p-value=0.0273) was not equal among quartiles of factor scores and seemed to increase from 1st to 4th quartile; mean foremilk omega-3/omega-6 ratio (p-value=0.009), LA/ALA ratio (p-value=0.0780), AA/EPA ratio (p-value=0.0012), AA/DHA ratio (p-value=0.0193), and LA/DHA ratio (p-value=0.0277) were not equal among quartiles of factor scores and seemed to decrease from 1st to 4th quartile;<br>PROTEINS AND FATTY ACIDS WITH LEGS: mean foremilk content in AA was not equal among quartiles of factor scores (p-value=0.0473) and seemed to increase from 1st to 4th quartile; mean foremilk content in omega-3 (p-value=0.0339), ALA (p-value=0.0354), EPA (p-value=0.0559), DHA (p-value=0.0640), AA/EPA ratio (p-value=0.0229), and AA/DHA ratio (p-value=0.0378) was not equal among

						quartiles of factor scores with unclear trends across quartiles;<br>FATTY ACIDS WITH FINS: mean foremilk content in omega-3 (p-value=0.0038), EPA (p-value=0.0004), DHA (p-value=0.0013), DPA (p-value=0.0276) was not equal among quartiles of factor scores and seemed to increase from 1st to 4th quartile; mean foremilk omega-3/omega-6 ratio (p-value=0.0426), AA/EPA ratio (p-value=0.0004), AA/DHA ratio (p-value=0.0006), and LA/DHA (p-value=0.0012) were not equal among quartiles of factor scores and seemed to decrease from 1st to 4th quartile;<br>FATTY ACIDS WITH LEAVES: mean foremilk content in SFA (p-value=0.0035), MUFA (p-value=0.0322), AA (p-value=0.0637), omega-3 (p-value=0.0735), ALA (p-value=0.0032) was not equal among quartiles of factor scores and seemed to decrease from 1st to 4th quartile for SFA, have unclear trend across quartiles for AA, and increase from 1st to 4th quartile for MUFA, omega-3, and ALA;<br>STARCH AND VEGETABLE PROTEINS: mean foremilk AA/EPA ratio was not equal among quartiles of factor scores (p-value=0.0910) and seemed to increase from 1st to 4th quartile
Lasalvia, 2021 (74)<br>Varese (Lombardy)<br>ROCAV<br>Good quality	PCFA<br>EIG>1, Scree plot and total variance explained<br>Varimax rotation<br>FL≥0.28 or FL≤-0.15	24.35% (4)	WESTERN: high on red meats, animal fats, processed meats, salty biscuits, vegetable oils, mayonnaise and other sauces, spirits, cheeses, eggs, pizza, crustaceans and molluscs, beer and	Multiple linear regression models to derive mean differences (and corresponding 95% CIs) in metabolic and inflammatory	Multiple linear and logistic regressions models on metabolic and inflammatory parameters and risk factors adjusted for age, sex, and total EI whenever	WESTERN (PC1): mean difference between 5th and 1st quintile category of factor scores was significantly different from 0 for total cholesterol (9.12, 95%CI: 3.26–14.98), LDL cholesterol (6.78, 95%CI 1.40–12.16), glucose (4.54, 95%CI: 2.22–6.86), BMI (0.86, 95%CI: 0.18–1.53), and leucocytes (0.62, 95%CI: 0.29–0.95); being in the 5th vs. 1st quintile of factor scores was

	Factorability checks NA<br>DP internal consistency NA<br>DP reproducibility NA		cider, offals, wine, soft drinks, sugar and sweets, and butter; low on toasted bread and rusks, and fruits;<br>MEDITERRANEAN: high on olive oil, cooked vegetables, raw vegetables, legumes, pasta and other grains, bouillon, cooked tomatoes, soups, fruits, fish, and potatoes; low on soft drinks;<br>CARBOHYDRATE: high on pasta and other grains, cooked tomatoes, bread, and animal fats; low on yogurt, fish, nuts and seeds, breakfast cereals, crustaceans and molluscs, tea, cooked vegetables, fruit juices, fruits, snacks, and eggs;<br>RESIDUAL: high on milk, coffee, and white meats; low on tea, wine, spirits	parameters and risk factors (dependent variables) namely total cholesterol, HDL cholesterol, LDL cholesterol, TGs, glucose, SBP, DBP, MBP, BMI, and leucocytes, between 5th and 1st quintile category of factor scores for the DPs with delta factor scores as independent variables; Multiple logistic regression to derive ORs of metabolic and inflammatory parameters and risk factors (dependent variables) namely ever smoking (vs never smoking), dyslipidemia, and hypertension according to adherence to DPs comparing	possible;<br>Multiple linear regression on carotid-femoral PWV adjusted for age, sex, EI, cigarette smoking, education level, BMI, hypertension and dyslipidemia in Model 3 and for age, sex, EI, cigarette smoking, education level, glucose and leucocytes in Model 4	associated with higher ODDS of being an ever-smoker (OR: 1.97, 95%CI: 1.43–2.72); a 1 SD increase in factor scores was associated with an increase in carotid-femoral PWV in Model 3 (beta=0.31, 95%CI: 0.11–0.52, p-value=0.003) and in Model 4 (beta=0.24, 95%CI: 0.03–0.45, p-value=0.03); leucocytes mediated 9.2% of the effect of factor scores on carotid-femoral PWV (Sobel test: p-value=0.047); glucose mediated 9.8% of the effect of factor scores on carotid-femoral PWV (Sobel test: p-value=0.059);<br>MEDITERRANEAN (PC2): mean difference between 5th and 1st quintile category of factor scores was significantly different from 0 for glucose (-3.03, 95%CI: -5.08 to -0.98), SBP (-2.49, 95%CI: -4.88 to -0.10), MBP (-1,73, 95%CI: -3.43 to – 0.03), and leucocytes (-0.57, 95%CI: -0.86 to -0.28); being in the 5th vs. 1st quintile of factor scores was associated with lower ODDS of being an ever-smoker (OR: 0.66, 95%CI: 0.50–0.88), and of hypertension (OR: 0.65, 95%CI: 0.48–0.86); a 1 SD increase in factor scores was borderline associated with a decrease in carotid-femoral PWV in Model 4 (beta=-0.17, 95%CI: -0.35 to 0.01, p-value=0.07);<br>CARBOHYDRATE (PC3): mean difference between 5th and 1st quintile category of factor scores was significantly different from 0 for SBP (2.49, 95%CI: 0.34–4.64), DBP (1.29, 95%CI: 0.11–2.48), MBP (1.89, 95%CI: 0.36–3.42), BMI (0.72, 95%CI: 0.19–1.25); being in the 5th compared to the 1st quintile of factor scores was associated with higher ODDS of hypertension (OR: 1.25, 95%CI: 0.97–1.62); a 1 SD increase in factor

				the 5th vs. the 1st quintile (reference) of factor scores; Multiple linear regression models assessing mean differences in carotid-femoral PWV (dependent variable) according to 1SD increase of DP scores (independent variables); Mediation analysis was conducted on BMI, hypertension, dyslipidemia, leucocytes, and glucose (possible mediators) using the procedures described in Preacher and Hayes and MacKinnon et al. to assess changes in the regression coefficient of Western DP with and without		scores was associated with an increase in carotid-femoral PWV in Model 3 (beta=0.11, 95%CI: -0.02 to 0.25, p-value=0.09); RESIDUAL (PC4): mean difference between 5th and 1st quintile category of factor scores was significantly different from 0 for HDL cholesterol (-3.62, 95%CI: -5.34 to -1.90), BMI (1.13, 95%CI: 0.59–1.66), and leucocytes (0.41, 95%CI: 0.15–0.68)

				including the mediator in the model; Sobel test to assess whether the indirect effect of the mediator on the relationship was significantly different from 0		
Zupo, 2020 (75) Castellana Grotte (Apulia) Apulia (from MICOL Study) Very good quality	PCA Percentage of explained variance Varimax rotation NA Descriptive labelling Factorability checks NA DP internal consistency NA DP reproducibility NA	NA% (5)	ENERGY-RICH: cured meat, sausages, lean ham, bacon, desserts, chocolate, and packaged/fried foods; FARM-HOUSE DIET: dairy products, vegetables, legumes, fruits, and semolina-type bread; SWEETS: desserts, chocolate, and package products; WINTER PATTERN: whole grains, poultry, fish, seafood, and legumes; ELDERLY PATTERN: whole milk, semolina-type bread, legumes, and vegetables	Multiple Cox proportional hazards model for overall mortality, including factor scores of each DPs as continuous independent variables in separate models; Multiple Cox proportional hazards model for cause-specific mortality including factor scores of FARM-HOUSE DIET DP as continuous independent variable	(Mutually) adjusted for sex, age, BMI, education level, smoking, multimorbidity, wine consumption, and olive oil consumption	ENERGY-RICH: overall mortality was borderline inversely related to factor scores (HR: 0.96, 95%CI: 0.92–1.01); FARM-HOUSE DIET: overall mortality was positively related to factor scores (HR: 1.05, 95%CI: 1.00–1.10); WINTER PATTERN: overall mortality was borderline inversely related to factor scores (HR: 0.97, 95%CI: 0.92–1.02); Other DPs did not provide additional evidence on overall and cause-specific mortality

Tatoli, 2022 (76)<br>Castellana Grotte (Apulia)<br>Apulia (including also a major part of MICOL Study participants)<br>Poor quality	Separate PCAs by diabetic status<br>Standardization NA<br>Subjective criteria (higher loadings in each group)<br>Varimax rotation NA<br>|FL|≥0.1<br>Factorability checks NA<br>DP internal consistency NA<br>DP reproducibility NA	NA%<br>(1 for each separate PCA)	DIABETIC/VEGETARIAN: dairy products, eggs, vegetables, nuts, legumes, potatoes, olive oil, fruits, sweets, and sugary foods;<br>NOT DIABETIC: white, red and processed meat, seafood, grains, sweets, sugary foods, caloric drinks, ready-to-eat dishes, wine, beer, and spirits	Not applicable	Not applicable	Not applicable<br>Internal reproducibility:<br>From visual inspection of PCA loadings across solutions, older subjects with diabetes had a healthier diet than their non-diabetic counterparts
Giontella, 2019 (77)<br>Verona (Veneto)<br>Good quality	PCA<br>Standardization<br>EIG>1, Scree plot NA<br>Varimax rotation<br>|FL|≥0.2<br>Factorability checks<br>DP internal consistency NA<br>DP reproducibility NA	45.5%<br>(2)	HEALTHY: vegetables, fresh and dried fruit, legumes, fish, dairy products, cereals and tubers, eggs, and meat;<br>UNHEALTHY: meat, fast food, sweets, cereals and tubers, eggs, fish, and dairy products	Spearman correlation coefficient to assess the association between one of hemodynamic or metabolic variables (BMI, z-score BMI, WHR, z-score WHR, brachial SBP, z-score brachial SBP, brachial DBP, z-score brachial DBP, central SBP, z-score central SBP,	Adjusted for age, sex, ethnicity, BMI, quartiles of total EI, and quartiles of Children-Physical Activity Questionnaire scores	HEALTHY: capillary glucose was inversely associated with factor scores in the Spearman analysis (r=-0.190, p<0.01) and in the multiple regression model (beta=-0.016, 95%CI: -0.027 to -0.005, p-value<0.01);<br>UNHEALTHY: brachial DBP (r=0.130, p<0.05) and z-score brachial DBP (r=0.130, p<0.05) were directly associated with factor scores in the Spearman analysis; multiple regression model confirmed borderline the results on brachial DBP (beta=0.911, 95%CI: -0.150–1.971, p-value=0.092), while results on z-score brachial DBP were not reported

				PWV (m/s), z-score PWV, capillary cholesterol, capillary TGs, and capillary glucose) and factor scores of each DP;<br>Multiple linear regression models with variables significantly related to DPs (based on Spearman correlation test) as dependent variables in separate models and factor scores of the interested DP as independent variable		
Turroni, 2021 (78)<br>Emilia-Romagna (Italy)<br>Good quality	PCFA<br>Standardization<br>EIG>1, Scree plot, and interpretability<br>Varimax rotation<br>|FL|≥0.63<br>Factorability checks<br>DP internal consistency	80.36% (3)	ANIMAL PRODUCTS: animal protein, cholesterol, niacin, zinc, SFAs, phosphorus, vitamin D, sodium, vitamin B6, retinol, riboflavin, thiamin, calcium, and LA;<br>VITAMINS AND FIBER: vitamin C, beta-carotene, total fiber, total folate,	Pearson correlation coefficients between EFA-based DP scores and daily amount of 37 selected food groups and condiments derived from the original FIs on the same	Not applicable	ANIMAL PRODUCTS: factor score was positively correlated with red meat (especially, beef and pork), offal, processed meat, fish, eggs, coffee, cheese, and olive oil;<br>VITAMINS AND FIBER: factor score was positively correlated with root vegetables, other (than citrus) fruit, olive oil, leafy vegetables (raw and cooked), cabbages, soups and bouillon;<br>REGIONAL: factor score was positively correlated with grains (whole meal), tea

	NA<br>DP reproducibility<br>NA		vitamin E, potassium, MUFAs, and soluble carbohydrates;<br>REGIONAL: vegetable protein, other PUFAs, and starch	subjects;<br>0.45 cut-off for identifying most correlated food groups		(including herbal tea), and leafy vegetables (raw and cooked)
Donati Zeppa, 2020 (79)<br>Urbino (Marche)<br>Fair quality	Principal Axis Factor Analysis<br>Standardization NA<br>Variables are expressed in terms of difference between values at time 3 (mean of the third mesocycle of training) and values at time 0 (mean of the 2 wks-before period)<br>EIG ≥ 1, variance explained<br>Descriptive labelling<br>Factorability checks<br>DP internal consistency<br>NA<br>DP reproducibility<br>NA	71.61% (3)	FACTOR 1: fat, protein, carbohydrate, energy, MUFAs, SFAs, and vitamin E;<br>FACTOR 2: PUFAs, omega 6, and omega 3;<br>FACTOR 3: soluble fiber, insoluble fiber, vitamin C, vitamin A, starch, and iron	Not applicable	Not applicable	Not applicable<br>Description of DPs found on the difference between the end and the beginning of the trial protocol, from visual inspection of Principal Axis Factor Analysis loadings

Colica, 2017 (80) Catanzaro (Calabria) Fair quality	PCA (not clear which dietary assessment tool is used) Standardization EIG≥1, Scree plot Varimax rotation |FL|>0.4 Factorability checks NA DP internal consistency NA DP reproducibility NA	55% (6)	PATTERN 1: meat, grains, olive oil, and potatoes; PATTERN 2: fish, vegetables, and milk; PATTERN 3: cheese, cakes, and fruit; PATTERN 4: cheese and animal-based fats; PATTERN 5: eggs, legumes, and wine; PATTERN 6: cakes, biscuits, and sugary drinks	Pearson's correlation coefficient to identify factor scores (as continuous variables) correlated to WB-BMD, to single and to multiple fractures; Multiple linear models to derive mean WB-BMD according to tertiles of factor scores for each DP, and post-hoc analysis to test the presence of differences in mean WB-BMD among pairs of tertiles; tests for trend were calculated by using DPs scores as a continuous variable after control for possible confounding factors; stratified analyses by sex; Multiple logistic	Multiple linear models were adjusted for BMI, glucose, creatinine, sex, medications, and current smoking; adjusted for age, current smoking, and medications when analyses restricted to Ms, and for current smoking, and medications only when analyses restricted to Fs; multiple logistic regression models on fractures were adjusted for BMI, sex, medications, and current smoking, but all previous variables were finally excluded from the model for multiple fractures	PATTERN 1: continuous factor scores were directly associated with WB-BMD (r=0.19, p=0.009); mean WB-BMD in the 3rd tertile of factor scores (mean: 1.070±0.01) was higher compared to the 1st (mean: 1.021±0.01), with p from post-hoc analyses equal to 0.040, and to the 2nd tertile (mean: 1.013±0.01), with p from post-hoc analyses equal to 0.019; and p-trend=0.043; in Fs mean WB-BMD in the 3rd tertile of factor scores (mean: 1.087±0.02) is higher compared to the 1st (mean: 1.016±0.02, p=0.009) and 2nd tertile (mean: 1.013±0.02, p=0.006), and p-trend of borderline significant equal to 0.08; continuous factor scores were inversely associated with multiple fractures (r=-0.32, p=0.038) as reported in the text; a one unit increase in factor scores was associated to a reduced risk of multiple fractures (OR: 0.28, 95%CI: 0.08–0.89);<br>PATTERN 2: continuous factor scores were borderline but directly associated with WB-BMD (r=0.14, p=0.064); mean WB-BMD in the 3rd tertile of factor scores (mean: 1.081±0.01) was higher compared to the 1st (mean: 1.023±0.01), with p from post-hoc analyses equal to 0.018, and to the 2nd tertile (mean: 1.000±0.01), with p from post-hoc analyses equal to 0.001), and p-trend=0.003; in Ms, mean WB-BMD in the 3rd tertile of factor scores (mean: 1.162±0.02) was higher compared to the 1st (mean: 1.088±0.03), with p from post-hoc analyses equal to 0.041, and non-significant p-trend; in Fs, mean WB-BMD in the 3rd tertile of factor scores (mean: 1.078±0.02) was higher compared to the 2nd (mean: 1.002±0.02), with p from post-hoc analyses

				regression models to estimate ORs of having at least one fracture (vs. no fractures) for the 1st or 2nd compared to the 3rd tertile of the factor scores for each DP;<br>Multiple logistic regression models to estimate ORs of having multiple fractures (vs. single fracture) according to increases in factor scores for each DP		equal to 0.006, and p-trend=0.023;<br>PATTERN 5: continuous factor scores were borderline but directly associated with WB-BMD (r=0.13, p=0.096); continuous factor scores were inversely associated with at least one fracture (r=-0.16, p=0.01); compared to the 3rd, being in the 1st or in the 2nd tertile of factor scores was associated with a reduced risk of at least one fracture (OR: 0.213, 95%CI: 0.065–0.703 and OR: 0.209, 95%CI: 0.064–0.675, respectively);<br>Other DPs did not provide additional evidence for the investigated outcomes
Mazza, 2017 (81)<br>Catanzaro (Calabria)<br>Good quality	Separate PCA on FGs and NUTs (not clear which dietary assessment tool is used)<br>Standardizatio n<br>EIG≥1, Scree plot<br>Varimax rotation<br>|FL|>0.40<br>Factorability checks (authors'	NA% (4+4)	FOOD-BASED PATTERNS:<br>CEREALS/MEAT/FIS H/OLIVE OIL PATTERN: cereals, meat, fish, and olive oil;<br>CAKES/FRUIT PATTERN: cakes and fruit;<br>ANIMAL FATS/MARGARINES PATTERN: animal fats and margarines;<br>LEGUMES PATTERN: legumes;<br>NUTRIENT-BASED	Pearson's correlations to identify food-based and nutrient-based DPs correlated with MMSE and ADAS-Cog (both at baseline and at follow-up);<br>Multiple linear regression models to assess the association of MMSE and	Multiple linear regression adjusted via stepwise based on correlations (excluded age, education level, and SBP for MMSE at follow-up for the LEGUMES PATTERN; included age and education level, but excluded waist circumference and glucose for ADAS-Cog at follow-up for the LEGUMES	FOOD-BASED DPs:<br>LEGUMES PATTERN: factor scores were directly associated with MMSE at baseline (r=0.15, p=0.062) and at follow-up (r=0.21, p=0.01), and inversely associated with ADAS-Cog at baseline (r=-0.12, p=0.068) and at follow-up (r=-0.23, p=0.004); a unit increase in factor scores was associated with improved MMSE at follow-up (B=0.23, 95%CI: 0.04–0.42, p=0.01) and with decreased ADAS-Cog at follow-up (B=-0.10, 95% CI: -1.79 to -0.30, p=0.006);<br>Other DPs did not provide additional evidence<br><br>NUTRIENT-BASED DPs:<br>PLANT PROTEINS AND PUFAS PATTERN:

	information: not reported in the article)<br>DP internal consistency NA<br>DP reproducibility NA		PATTERNS:<br>ANIMAL PROTEIN PATTERN: animal protein;<br>VEGETAL OILS PATTERN: vegetal oils;<br>FATS PATTERN: fats;<br>PLANT PROTEINS/POLYUNSATURATED FATS PATTERN: plant proteins, PUFAs	ADAS-Cog (both after 1 y) as separate dependent variables with each DP selected through correlation coefficients and related statistical tests on correlation coefficients;<br>Multiple logistic regression models to estimate ORs of ADAS-Cog improvement at 1 y (vs. non-improvement) according to continuous factor scores of each DP as independent variable	PATTERN);<br>Multiple logistic regression adjusted for education level, but excluded SBP, DBP, and waist circumference for PLANT PROTEINS AND PUFAS PATTERN	factor scores were directly associated with improved ADAS-Cog (r=0.18, p=0.030); a unit increase in factor scores was associated with improved ADAS-Cog with a significant OR=1.79 and corresponding beta=0.589 (95%CI: 0.04–0.42, p=0.045);<br>Other DPs did not provide additional evidence
Palli, 2001 (82)<br>Florence (Tuscany)<br>Good quality	EFA<br>Energy adjustment (residual method)<br>EIG>NA,<br>Scree plot NA,<br>interpretability NA<br>Varimax rotation<br>|FL|≥0.40	75.3% (4)	VITAMIN-RICH: sugar, fiber, vitamin C, vitamin E, beta-carotene, and nitrates;<br>TRADITIONAL: total protein, starch, alcohol, nitrite, and N-nitrosodimethylamine;<br>REFINED: total protein, SFAs, other PUFAs, cholesterol, sugar, retinol, vitamin	Multiple logistic regression models to derive ORs of gastric cancer according to tertiles computed among controls of factor scores for each DP in separate	Adjusted for age, sex, social class, family history of gastric cancer, area of residence, BMI tertiles, and EI	VITAMIN-RICH: OR 0.5 (95% CI: 0.4–0.7) of gastric cancer for 3rd vs. 1st tertile, p-trend=0.0003;<br>TRADITIONAL: OR 3 (95% CI: 1.8–4.8) of gastric cancer for 3rd vs. 1st tertile, p-trend=0.0001;<br>Other DPs did not provide additional evidence;<br><br>Attributable fraction: 25.9% (95% CI: 8.8–43.0) of cases was attributable to the VITAMIN-RICH DP; 38.9% (95% CI: 21.7–

	Factorability checks NA<br>DP internal consistency NA<br>DP reproducibility NA		E, vitamin D, and N-nitrosodimethylamine;<br>FAT-RICH: SFA, oleic acid, MUFAs, LA, ALA, cholesterol, and vitamin E	models; attributable risks computed by the method of Bruzzi et al. and the corresponding 95% CI estimated, as described by Benichou and Gail		56.1) of cases was attributable to the TRADITIONAL DP; when considered together, 44% (95% CI: 18.7–69.5) of cases was attributable to VITAMIN RICH and TRADITIONAL DPs
Anelli, 2022 (83)<br>Milan (Lombardy), Naples (Campania)<br>GIFt Study<br>Very good quality	PCA on the overall sample<br>Energy adjustment (NA method) on FGs from FFQ<br>EIG≥1.1,<br>Scree plot NA<br>Rotation NA<br>Descriptive labelling<br>Factorability checks NA<br>DP internal consistency NA<br>DP reproducibility NA	33,4% (3)	HIGH MEAT, ANIMAL FATS, GRAIN: meat, animal fats, and grains;<br>HIGH FISH, FRUIT, NUTS: fish, fruit, and nuts;<br>HIGH EGGS AND SWEETS, LOW LEGUMES: high on eggs and sweets; low on legumes	Multiple linear models with log-transformed variables if non-normally distributed, to derive biomarker (red blood cells folate (ng/mL), serum vitamin D (in µg/L), plasma hepcidin mature form (ng/mL), and plasma total antioxidant capacity (mM) concentrations at 29±2 gwks and delivery outcomes (maternal GWG in kg, gestational age at delivery in wks, placental	Adjusted for maternal age, pre-gestational BMI, education, working status, parity, geographical area, folic acid/multivitamin supplement use, and total EI; further adjustment for gestational age at blood sampling in case of biomarkers analyses, and for gestational age at delivery and GWG whenever possible in case of delivery outcomes analyses	HIGH MEAT, ANIMAL FATS, GRAIN: factor scores were associated directly with plasma hepcidin concentrations (beta=0.3, 95%CI: 0.0–0.5, p-value<0.05) and inversely with serum vitamin D concentrations (beta=-3.9, 95%CI: -6.9 to -0.9, p-value<0.05) and with gestational age at delivery in pregnancies carrying F fetuses only (beta=-0.5, 95%CI: -0.9–0.0, p-value<0.05); stratified analyses for geographical subgroups provided comparable results;<br>Other DPs did not provide additional evidence<br>Cross-study reproducibility:<br>HIGH EGGS AND SWEETS, LOW LEGUMES: participants from Naples showed a different adherence compared to Milan (Mann–Whitney U test: p=0.01), reported to be higher for Naples in the text

				weight in grams, neonatal ponderal index in grams/cm^3, neonatal lengths in cm, and neonatal head circumference in cm) according to continuous factor scores of each DP as independent variable in separate models; stratified analyses by geographical area and by fetus sex for gestational age at delivery; Cross-study reproducibility of DPs: hypothesis test on (continuous) component scores by geographical area with Mann–Whitney U test		
Ruggieri, 2022 (84) Crotone (Calabria), Milazzo and	PCA Standardization NA EIG>NA, Scree plot NA, interpretability	24.9% (3)	PRUDENT: stem-leafy-cooked-raw vegetables, cauliflower, blue fish, fresh caught-farmed fish, fruit, legumes,	Not applicable	Not applicable	Not applicable

Augusta-Priolo (Sicily) NEHO Study Good quality	NA Rotation NA Descriptive labelling Factorability checks NA DP internal consistency NA DP reproducibility NA		beef, and yogurt; HIGH ENERGY: high on salty snacks, bakery products, cold meats, fries, mayonnaise, soft drinks, bread, butter, pasta, fresh and aged cheese, potatoes, and pork; low on cereals; VEGETARIAN: high leafy-cooked-raw vegetables, lamb and mutton, tubers, fries, cereals, dried fruit, eggs, oil, butter, and potatoes; low on beef and fresh farmed fish			

ABBREVIATIONS: AA, arachidonic acid; ACPA, anti-citrullinated protein antibodies; ADAS-Cog, Alzheimer's Disease Assessment Scale - Cognitive sub-scale; ALA, alpha-linolenic acid; ANOVA, analysis of variance; ATBC, Alpha-Tocopherol Beta-Carotene; AUFA, Animal Unsaturated Fatty Acids; BMI, body mass index; CHD, coronary heart disease; CI, confidence interval; CIN, cervical intraepithelial neoplasia; COVID-19, Coronavirus disease 2019; CRP, C-reactive protein; CVD, cardiovascular disease; DAFNE, Data Food Networking; DAS28-CRP, Disease Activity Score on 28 joints and C-reactive protein; DBP, diastolic blood pressure; DHA, docosahexaenoic acid; DIETSCAN, Dietary Patterns and Cancer; DMARDs, disease modifying anti-rheumatic drugs; DP, dietary pattern; DPA, docosapentaenoic acid; EFA, Exploratory Factor Analysis; EI, energy intake(s); EIG, eigenvalue; EPA, eicosapentaenoic acid; EPIC, European Prospective Investigation into Cancer and Nutrition; F, female(s); FA, fatty acid(s); FAC, food antioxidant content; FG, food group(s); FI, food item(s); FL, factor loading(s); FSIQ, full scale intelligence quotient; GIFt, Gestational Intake of Food towards healthy outcomes; GPA, Grade Point Average; GWG, gestational weight gain; gwk, gestational week(s); HDL, high-density lipoprotein; HER2, human epidermal growth factor receptor 2; HR, hazard ratio; hrHPV, high-risk Human Papilloma Virus; HRT, hormone replacement therapy; IDEFICS, Identification and prevention of Dietary- and lifestyle-induced health EFfects In Children and infantS; LA, linoleic acid; LDL, low-density lipoprotein; LINE-1, Long Interspersed nuclear elements-1; M, male(s); MAMI-MED, Multisettoriale Alla salute Materno-Infantile Mediante valutazione dell'Esposoma nelle Donne; MBP, mean blood pressure; MICOL, Multicenter Italian Study on Epidemiology of Cholelithiasis; MMSE, Mini Mental State Examination; MUFA, monounsaturated fatty acid(s); NA, not available; NEHO, Neonatal Environment and Health Outcomes; NLSC, Netherlands Cohort Study; NUT, nutrient(s); OC, oral contraceptive; OR, odds ratio; ORDET, Ormoni e Dieta nell'Eziologia del Tumore della Mammella; PA, physical activity; PAL, physical activity level; PC, principal component; PCA, Principal Component Analysis; PCFA, Principal Component Factor Analysis; PE, Physical Education; PRI, perceptual reasoning index; PSI, processing speed index; PUFA, polyunsaturated fatty acid(s); PWV, pulse wave velocity; RF, rheumatoid factor; ROCAV, Risk Of Cardiovascular diseases and abdominal aortic Aneurysm in Varese; RR, relative risk; SBP, systolic blood pressure; SD, standard deviation; SDAI, Simplified Disease Activity Index; SE, standard error; SES, socioeconomic status; SFA, saturated fatty acid(s); SMC, Swedish Mammography Cohort; TG, triglyceride(s); VCI, verbal comprehension index; vs., versus; VUFA, Vegetable Unsaturated Fatty Acids; WB-BMD, whole-body bone mineral density; WHR, waist-to-hip ratio; WISC-IV, Wechsler Intelligence Scale of Children; wk, week(s); WMI, working memory index; y, year(s)

Supplemental Figure 1. Roadmap and specific research questions from the systematic review on *a posteriori* dietary patterns identified with principal component analysis and/or exploratory factor analysis in Italy, as organized across the two companion articles dealing with this topic. The grey boxes highlight results that have already been introduced in the companion article[1]

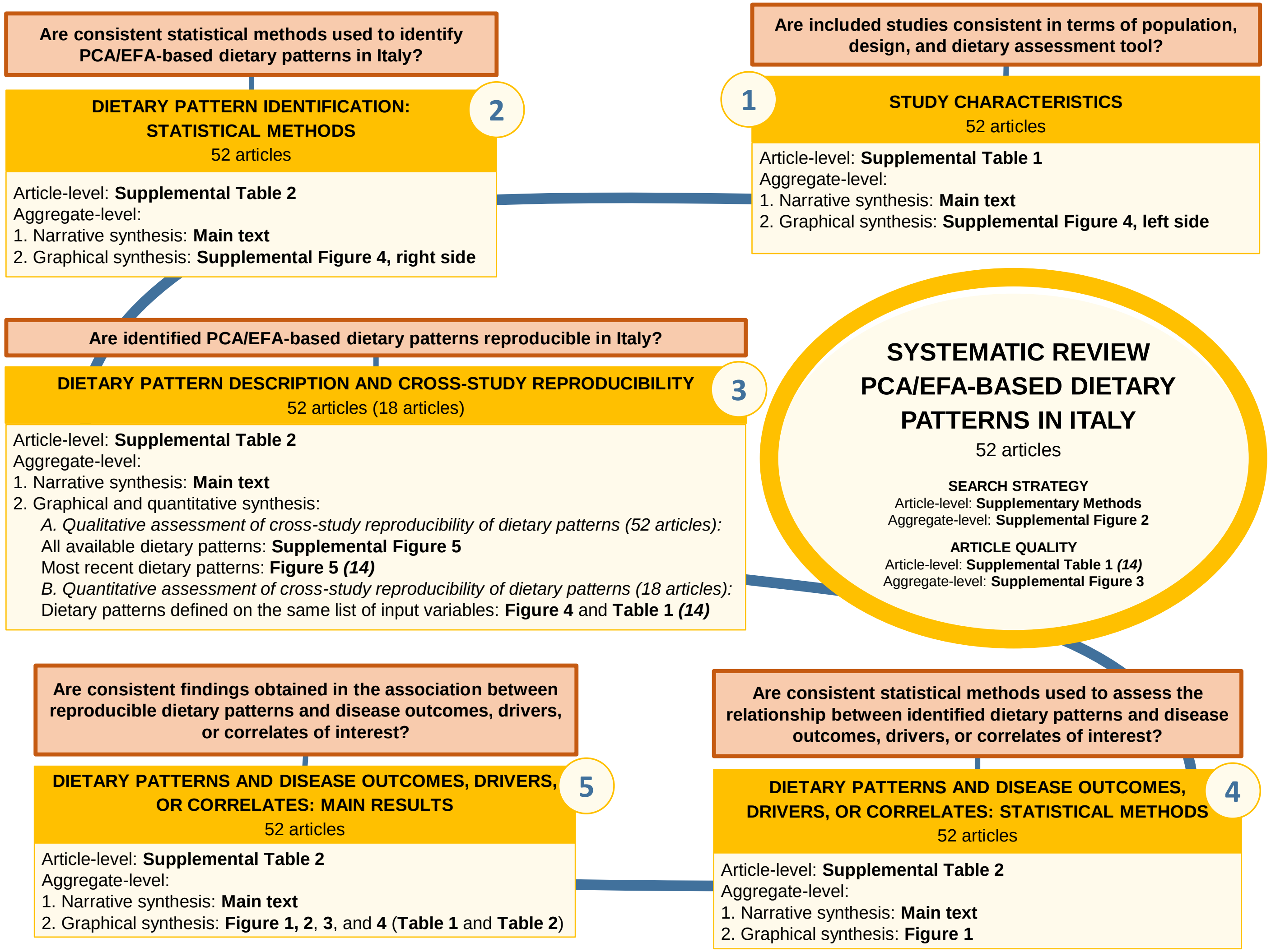

[1]In the blocks we described the single aspects summarized in the systematic review: study characteristics, dietary pattern identification method, dietary pattern description and *cross-study reproducibility*, statistical methods used to assess the relationships between identified dietary patterns and disease outcomes/drivers/correlates of interest, and the main results concerning these relationships. For each block, we reported the number of articles contributing to the analysis, along with the article-level and aggregate-level information gathered across those articles. The specific research questions were summarized at the top of each box and presented in a logical flow (indicated by a solid arrow), to highlight how all research questions contributed to the final evaluation of the consistency of associations between identified dietary patterns and disease outcomes/dietary pattern drivers/correlates of interest
ABBREVIATIONS: PCA, principal component analysis; EFA, exploratory factor analysis

Supplemental Figure 2. Flow diagram of the study selection process

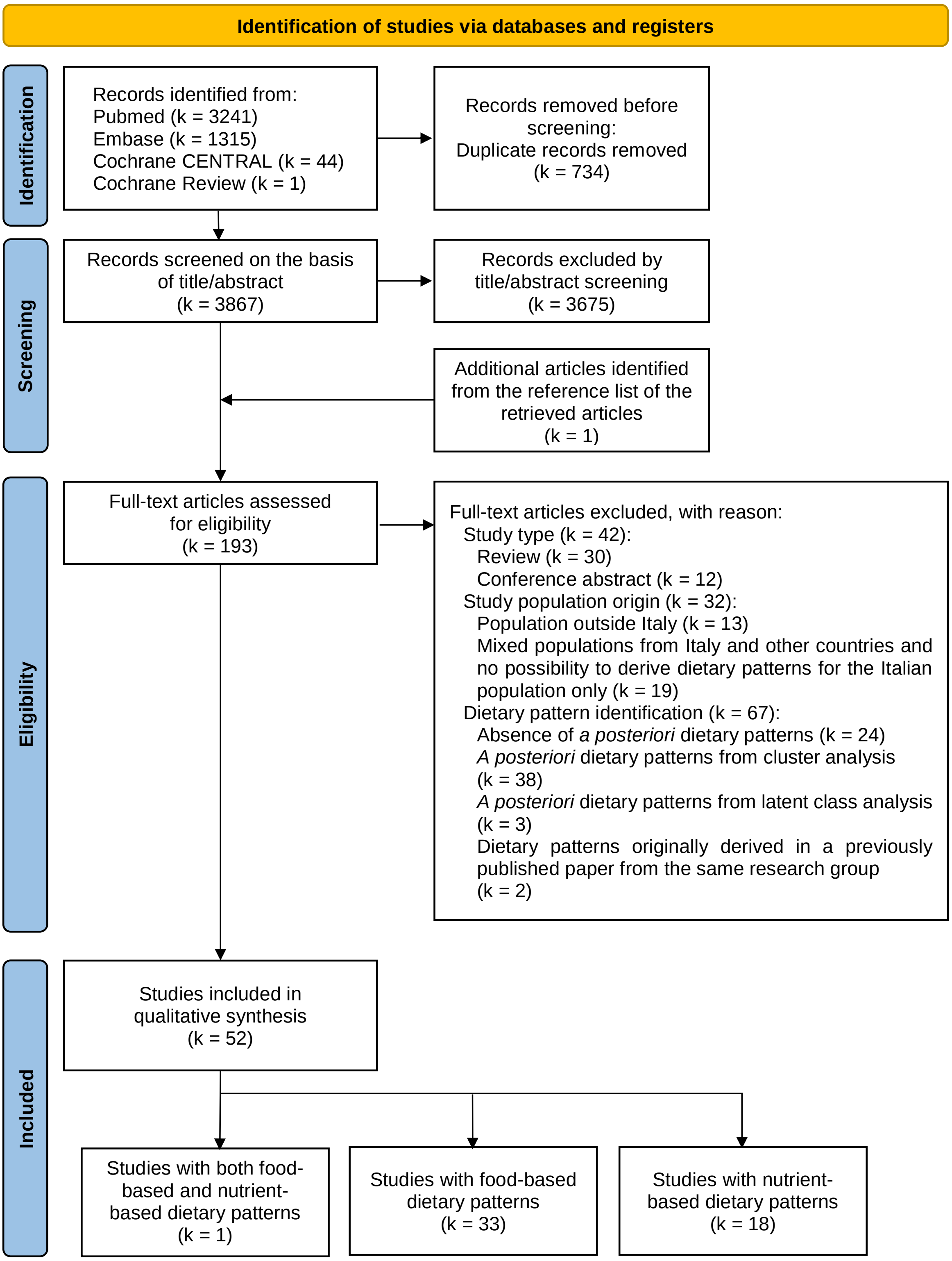

Page MJ, McKenzie JE, Bossuyt PM, Boutron I, Hoffmann TC, Mulrow CD, et al. The PRISMA 2020 statement: an updated guideline for reporting systematic reviews. BMJ 2021;372:n71. doi: 10.1136/bmj.n71

ABBREVIATIONS: EMBASE, Excerpta Medica Database; PRISMA, Preferred Reporting Items for Systematic Reviews and Meta-Analyses

Supplemental Figure 3. Summary of quality assessment for studies included in the systematic review by single rating tool available from the National Institutes of Health, National Heart, Lung, and Blood Institute

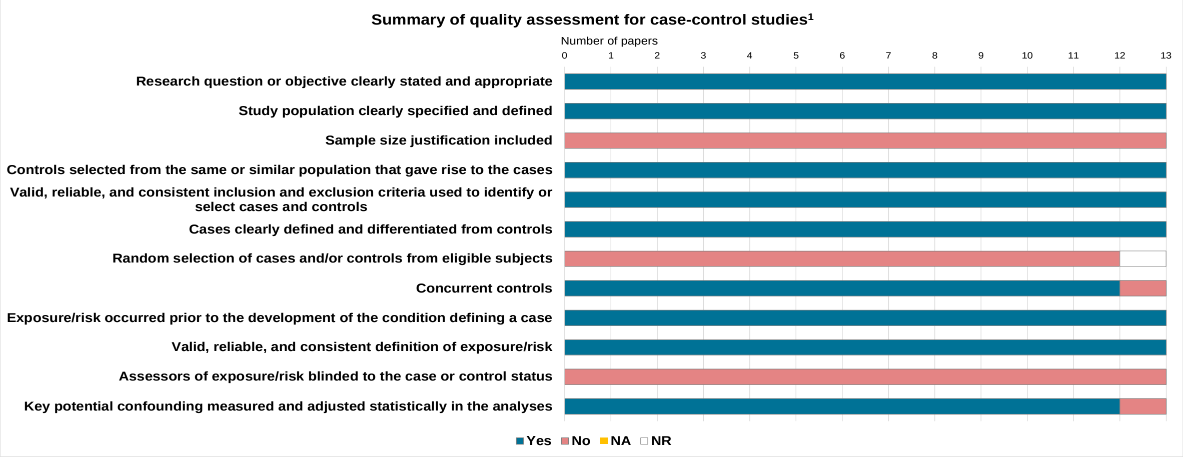

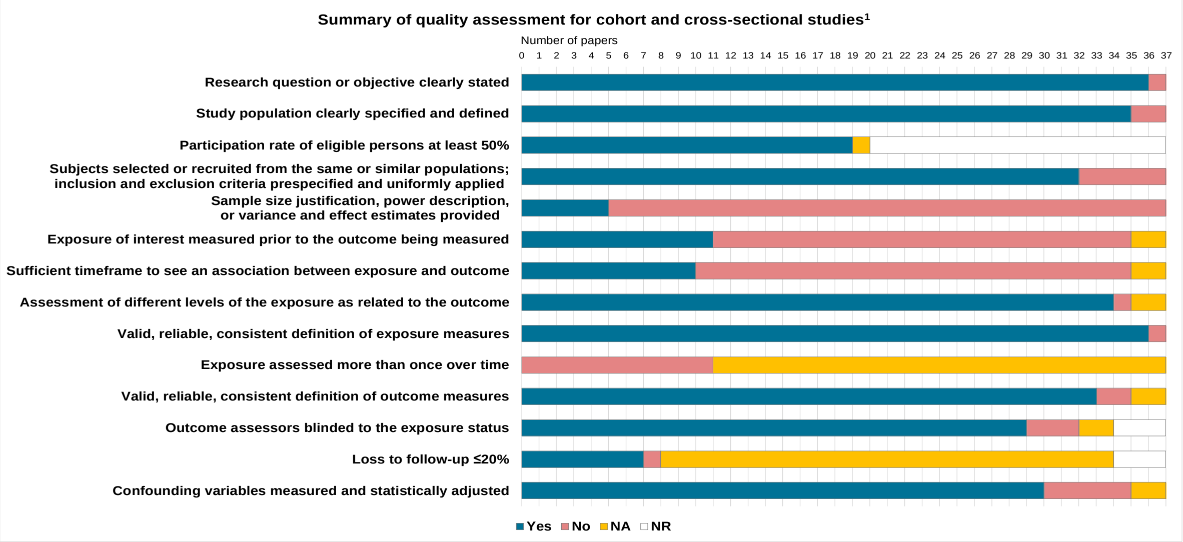

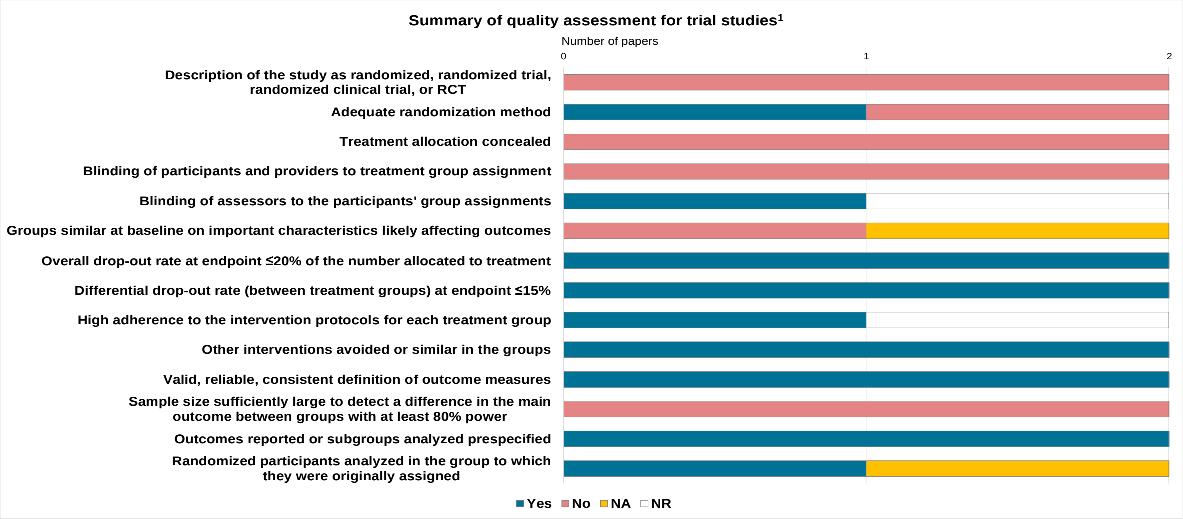

[1]For each quality assessment tool, each row reported the distribution of replies (“Yes”, “No”, “Not applicable”, and “Not reported”) to single questions. The “Cannot determine” reply was never used during this quality assessment.
ABBREVIATIONS: NA, Not Applicable; NR, Not reported

Supplemental Figure 4. General characteristics of the studies included in the systematic review and main steps in the dietary pattern identification process: a summary of findings from the systematic review

STUDY CHARACTERISTICS

STUDY NAME
Moli-sani (k=8)
EPIC-Elderly (k=2)
EPIC-InterAct (k=1)
DIETSCAN (k=2)
ORDET (k=2)[1]
Seven Countries Study (k=2)
Mamma & Bambino (k=2+1)[2]
IDEFICS (k=1)
DAFNE (k=1)
MEDIDIET (k=1)
ROCAV (k=1)
Salus in Apulia Study (k=2)
GIFt Study (k=1)
NEHO Study (k=1)
Not specified (k=24)

STUDY POPULATION
Children (k=3)
Adolescents (k=1)
Pregnant or breastfeeding women (k=6)
Non-pregnant women only (k=9)
Men only (k=3)
General adults (males and females) (k=24)
Elderly only (k=5)
Entire household (0-75 years) (k=1)

STUDY LOCATION
Italy (k=41, distribution by region as displayed below)
International projects including Italy (k=11)

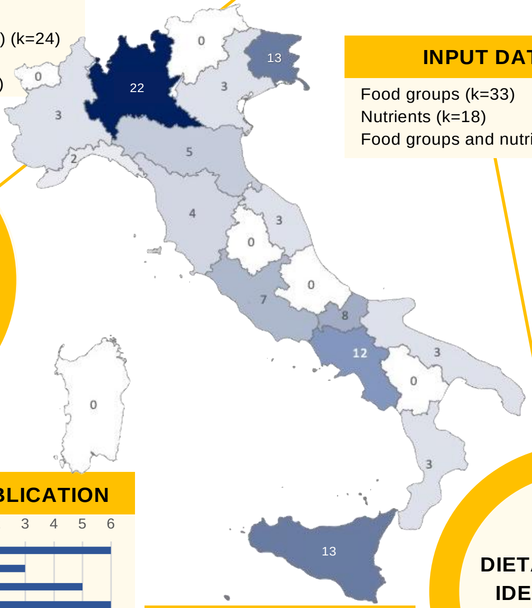

STUDY DESIGN
Trial (k=2)
Prospective cohort (k=19)
Case-control (k=13)
Case-cohort (k=1)
Cross-sectional (k=17)

DIETARY ASSESSMENT TOOL
FFQ (k=44)
Dietary record (k=1)
Diet history (k=2)
24-hour recall (k=1)
Country-specific household survey (k=1)
More than one tool (k=3)

OUTCOMES AND CORRELATES
Incidence of chronic diseases and overall/cause-specific mortality (k=24)
Cardiovascular or metabolic risk factors (k=3)
Pregnancy-related outcomes (k=3)
Cognitive performance (k=2)
Quality of life (k=2)
Fractures (k=1)
Rheumatoid arthritis activity (k=1)
Dietary pattern drivers/correlates (mainly socio-economic ones) (k=10)
No outcomes/correlates (k=6)

STUDY PUBLICATION

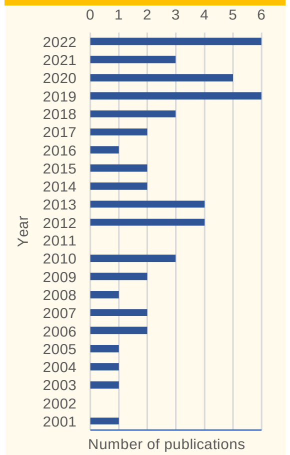

DIETARY PATTERN IDENTIFICATION METHOD

INPUT PREPROCESSING
No (k=11)
Standardization (k=30)
Energy adjustment (any method) (k=3)
Combination of previous pre-processing methods (k=8)

INPUT DATA
Food groups (k=33)
Nutrients (k=18)
Food groups and nutrients (k=1)

METHOD
PCA (k=10)
EFA (k=41)
PCA and EFA (k=1)

ESTIMATION METHOD (FOR EXPLORATORY FACTOR ANALYSIS ONLY)
PCA (k=40)
Principal Axis Factor (k=1)

CRITERIA TO DEFINE THE NUMBER OF COMPONENTS/ FACTORS TO RETAIN
Single criterion (k=6)
Combination of 2 criteria (k=8)
Combination of 3 criteria (k=35)
Not reported (k=3)

ROTATION
No (k=7)
Orthogonal (e.g., varimax) (k=45)

LABELLING
Descriptive (k=4)
Pre-specified quantitative cut-off on loadings (k=48)

CHECKS
FACTORABILITY
No (k=35)
Yes (k=17)

INTERNAL CONSISTENCY
No (k=38)
Yes (k=14)

REPRODUCIBILITY
Internal (k=28)
Cross-study (k=2)
Internal and cross-study (k=2)
Not reported (k=20)

[1]The DIETSCAN project included one Italian cohort – the ORDET one – which recruited women only and it was therefore classified as "non-pregnant women only" instead of "general adults (males and females)"
[2]The Mamma & Bambino birth cohort was also pooled together with MAMI-MED in another study [Magnano San-Lio et al. (66)]
ABBREVIATIONS: DAFNE, Data Food Networking; DIETSCAN, Dietary Patterns and Cancer; EFA, Exploratory Factor Analysis; EPIC, European Prospective Investigation into Cancer and Nutrition; FFQ, Food Frequency Questionnaire; GIFt, Gestational Intake of Food towards healthy outcomes; IDEFICS, Identification and prevention of Dietary- and lifestyle-induced health EFfects In Children and infantS; NEHO, Neonatal Environment and Health Outcomes; ORDET, Ormoni e Dieta nell'Eziologia del Tumore della Mammella; PCA, Principal Component Analysis; ROCAV, Risk Of Cardiovascular diseases and abdominal aortic Aneurysm in Varese

Supplemental Figure 5. Qualitative assessment of reproducibility for all the available dietary patterns: dietary patterns identified using principal component analysis or exploratory factor analysis in Italy from 1965 to 2022, in groups based on original text descriptions and loadings[1]

FOOD-BASED DIETARY PATTERNS

MEDITERRANEAN-STYLE

MIXED-SALAD

- **SALAD VEGETABLES (60, 61): olive oil, raw and cooked leafy vegetables**, mixed vegetables in salad, raw tomatoes, raw carrots, and other fruiting vegetables
- **(SALAD) VEGETABLES (58, 59): oil, raw leafy vegetables**, carrots, tomatoes, dressings
- **OLIVE OIL AND SALAD (55, 56): olive oil, raw leafy vegetables**, raw tomatoes, root vegetables, soup, chicken and turkey
- **PC1 (57): vegetable oils, leafy vegetables**, fruiting vegetables, cabbage, other vegetables, legumes, fish
- **OLIVE OIL AND VEGETABLES (47-54): olive oil, raw and cooked vegetables**, legumes, soups, fruits, fish, potatoes, bouillon, white meat, crustaceans and molluscs, crisp bread and rusks, nuts and dried fruits, yogurt, snacks, and fresh cheese

HEALTHY-PROTEIN FOODS AND SIDE DISH

- **PRUDENT (55, 56): cooked leafy vegetables, legumes**, cabbage, mushrooms, other vegetables, onions and garlic, **fish**, crustaceans and molluscs, seed oil, cooked tomatoes, fresh fruit (non-citrus), and nuts and seeds
- **HIGH FISH, FRUIT, NUTS (83): fish**, fruit, nuts
- **PRUDENT (66): raw and cooked vegetables, legumes, soup, fish**, fruit
- **PRUDENT (68): potatoes, raw and cooked vegetables, legumes, vegetable soups**, olive oil
- **PRUDENT (69): potatoes, raw and cooked vegetables, legumes, soup, fish**
- **LEGUMES, VEGETABLES AND FISH (67): potatoes, raw and cooked vegetables, legumes, vegetable soup, fish**
- **PRUDENT (65): potatoes, raw and cooked vegetables, legumes, soup**, rice
- **PRUDENT (64): potatoes, cooked vegetables, legumes, soup**, pizza
- **COOKED VEGETABLES (58): potatoes,** cabbages, carrots, **cooked leafy vegetables, legumes, fish**, rice, poultry
- **PRUDENT (60, 61):** high: **potatoes, cooked leafy vegetables**, cooked and raw carrots, other fruiting vegetables, **pulses, fish,** rice, poultry, yogurt, olive oil; low: wine and spirits
- **MEDITERRANEAN (PC2) (74):** positive: **potatoes, raw and cooked vegetables, legumes, soups,** olive oil**, fish,** fruits, pasta and other grains, bouillon, cooked tomatoes; negative: soft drinks
- **FACTOR 2/FA2/PC2 (62, 63): potatoes, vegetables, fish, bread, cereals,** oils
- **WINTER PATTERN (75): legumes, fish,** seafood, **whole grains**, poultry
- **PATTERN 2 (80): fish, vegetables,** milk
- **CEREALS/MEAT/FISH/OLIVE OIL PATTERN (81): cereals, fish**, olive oil, meat
- **PRUDENT (84):** stem-leafy-**cooked-raw vegetables**, cauliflower, **legumes**, blue **fish**, fresh caught-farmed **fish**, fruit, beef, yoghurt
- **WIDE RANGE (72): vegetables, fish**, fruits, cereals, meat, dairy products
- **VEGETARIAN (84):** high: **potatoes, tubers,** leafy-**cooked-raw vegetables**, cereals, lamb and mutton, fries, dried fruit, eggs, oil, butter; low: beef and fresh farmed fish
- **DP DIABETIC (76): potatoes, vegetables, legumes, olive oil**, fruits, nuts, dairy products, eggs, sweets and sugary foods
- **PRUDENT (70): potatoes, cooked vegetables, legumes**, fruits, nuts, yogurt, offals, shellfish, tea
- **HEALTHY (77): cereals and tubers, vegetables, legumes, fish**, fresh and dried fruit, dairy products, eggs, meat
- **HEALTHY (71): raw vegetables, cooked vegetables and beans, fresh or frozen fish (not fried)**, fresh fruits without added sugar, fresh meat (not fried)

TRADITIONAL PATTERNS

- **FARM-HOUSE DIET (75): legumes, dairy products, vegetables, semolina type bread**, fruits
- **ELDERLY PATTERN (75): legumes, whole milk, vegetables, semolina type bread**
- **PATTERN 5 (80): legumes**, eggs, wine
- **LEGUMES PATTERN (81): legumes**

WESTERN-STYLE

PASTA-AND-MEAT-ORIENTED

- **PATTERN 1 (80): grains, meat**, olive oil, potatoes
- **HIGH MEAT, ANIMAL FATS, GRAIN (83): grains, meat, animal fats**
- **PC2 (57): pasta and rice, red meat**, processed meat, **other fats**, sugar
- **CANTEEN (60, 61): pasta, cooked tomatoes, veal, wine**, olive oil, pulses, other fruiting vegetables, bread
- **PASTA AND MEAT (47-54):** high: **pasta and other grains, cooked tomatoes, red meat**, white meat, offals, **processed meat, animal fats, bread, wine**, beer, olive oil, other sauces, seasoned cheese; low: breakfast cereals and yogurt
- **PASTA AND MEAT (55, 56):** high: **pasta, cooked tomatoes, beef, pork, processed meat, other animal fats, white bread, wine**; low in yogurt
- **PORK, PROCESSED MEAT, POTATOES (58): butter**, non-fermented whole milk, **cakes, eggs, pasta, beef, potatoes, processed meat**
- **PORK, PROCESSED MEAT, POTATOES (59): butter, pasta, potatoes, beef and veal, processed meat**
- **WESTERN (60, 61): butter, potatoes**, other **pasta, processed meat, veal, eggs, cakes, beef**, seed oils, offal, **pork, cheese**

CARBOHYDRATE (PC3) (74): positive: **pasta and other grains, cooked tomatoes, animal fats,** bread; negative: yogurt, fish, nuts and seeds, breakfast cereals, crustaceans and molluscs, tea, cooked vegetables, fruit juices, fruits, snacks, eggs

DAIRY PRODUCTS AND SWEETS

- **SWEET & DAIRY (55, 56): cakes and puddings, sugar** and honey and jam, **chocolate-based** confectionery, **patisserie and biscuits, milk, cheese**, eggs, butter, ice cream, coffee, processed meat
- **FACTOR 1 (62): pastries, sugar, milk, cheese,** meat, fruit
- **RESIDUAL (PC4) (74):** positive: **milk, coffee**, white meats; **negative**: tea, **wine, spirits**
- **PATTERN 3 (80): cheese, cakes**, fruit
- **PATTERN 6 (80): cakes, biscuits**, sugary drinks
- **CAKES/FRUIT PATTERN (81): cakes**, fruit
- **SWEETS (75): desserts, chocolate, package products**
- **EGGS AND SWEETS (47-54): eggs, margarines, sugar and sweets,** processed meat, vegetable oils, snacks, mayonnaises, **butter**, seasoned and fresh **cheese**, pizza, canned fish, fruit juices, coffee, soft drinks, potatoes, white meat, red meat, animal fats, bread, and beer
- **HIGH EGGS AND SWEETS, LOW LEGUMES (83):** high: **eggs and sweets**; low: legumes
- **FACTOR 3 (62): eggs,** alcoholic beverages
- **PATTERN 4 (80): animal-based fats,** cheese
- **ANIMAL FATS/MARGARINES PATTERN (81): animal fats/margarines**
- **SPREADS (71): butter and/or margarine on bread**, reduced-fat products on bread, **jam and honey, chocolate or nut-based spread**

UNHEALTHY FOODS AND SNACKS

- **SNACK FOODS, PROCESSED MEATS AND OILS (67): red meat, processed meat, chips, dipping sauces, snacks, vegetable oils, sugar and sweets**, fruit juice
- **WESTERN (64, 65): red meat, fries, dipping sauces, salty snacks**, alcoholic drinks
- **WESTERN (68):** high: **processed and red meats, chips, dipping sauces, snacks, plant oils**; low: olive oil
- **WESTERN (66): fries, dipping sauces, salty snacks, vegetable oil, sweets**, white bread
- **WESTERN (69):** high: **processed meat, dipping sauces, salty snacks, vegetable oil**, alcoholic drinks, canned fish; low fruits
- **WESTERN (PC1) (74):** positive: **red meats, processed meats, mayonnaise and other sauces, salty biscuits, vegetable oils, sugar and sweets, soft drinks**, spirits, beer and cider, wine, cheeses, eggs, butter, pizza, crustaceans and molluscs, offals; negative: toasted bread and rusks, fruits
- **HIGH ENERGY (84):** high: **cold meats, fries, mayonnaise, salty snacks, bakery products, soft drinks**, bread, butter, pasta, fresh and aged cheese, potatoes, pork; low: cereals
- **ENERGY-RICH (75): cured meat, sausages, lean ham, bacon, packaged/fried foods, desserts, chocolate**
- **DP NOT DIABETIC (76): white, red and processed meat, sweets, sugary foods, caloric drinks,** ready-to-eat dishes, wine, beer and spirits, seafood, grains
- **BEVERAGE AND CONVENIENCE (72):** high: **beverages (alcoholic and nonalcoholic)** and ready-to-eat dishes; low: plant foods and elaborate-to-cook dishes
- **WESTERN (70): red and processed meat, dipping sauces, fries, vegetable oil,** white bread, shellfish
- **ENERGY DENSE (70): dipping sauces, fries, sweets and refined sugar**, pizza, yogurt, butter and margarine
- **PROCESSED (71): ketchup, mayonnaise and mayonnaise-based products, crisps, corn crisps and popcorn, chocolate and candy bars, sweetened drinks;**
- **UNHEALTHY (77): meat**, fast food, **sweets**, cereals and tubers, eggs, fish, dairy products

ALCOHOL (58): high: **wine and spirits**; low: coffee (with milk), non-fermented lowfat milk, cakes, other fruits

NUTRIENT-BASED DIETARY PATTERNS

VEGETABLE-ORIENTED

VEGETABLE-BASED PATTERNS

- **VITAMINS AND FIBER (33): vitamin C, total fiber, β-carotene equivalents, total folate**, potassium, soluble carbohydrates, vitamin B6
- **VITAMINS AND FIBER (34, 39, 41, 43): vitamin C, total fiber, β-carotene equivalents, total folate**, potassium, soluble carbohydrates
- **VITAMINS AND FIBER (37, 42): vitamin C, total fiber, β-carotene equivalents, total folate**
- **VITAMINS AND FIBER (36, 38, 40): vitamin C, total fiber, β-carotene equivalents, total folate**, soluble carbohydrates
- **VITAMINS AND FIBER (35): vitamin C, total fiber, β-carotene equivalents**, soluble carbohydrates
- **VITAMINS AND FIBER (44): vitamin C, total fiber, β-carotene equivalents, total folate**, potassium, vitamin E
- **VITAMINS, MINERALS AND FIBERS (73): vitamin C, fiber, β-carotene equivalents, folate**, potassium, vitamin E, iron
- **VITAMINS AND FIBER (78): vitamin C, total fiber, β-carotene, total folate**, potassium, vitamin E, MUFA, soluble carbohydrates
- **ANTI-OXIDANT VITAMINS AND FIBER (45): vitamin C, soluble and insoluble fiber**, potassium, soluble carbohydrates, vitamin A (RAE), lignans, flavonoids
- **VITAMIN-RICH (82): vitamin C, fiber, β-carotene**, sugar, vitamin E, nitrates
- **FACTOR 3 (79): vitamin C, soluble fiber, insoluble fiber**, vitamin A, starch, iron
- **PLANT-BASED FOODS (46): vitamin C, total fiber, β-carotene, folate, potassium,** vitamin E, iron

ANIMAL-ORIENTED

ANIMAL-SOURCE FATTY ACIDS

- **AUFA (36, 37, 40, 42, 44): other PUFA, vitamin D**
- **OTHER PUFA AND VITAMIN D (38, 41): other PUFA, vitamin D**, niacin
- **AUFA (45): EPA, DHA, vitamin D**
- **FATTY ACIDS WITH FINS (73): EPA, DHA,** DPA, **vitamin D**
- **FACTOR 2 (79): PUFA, ω6, ω3**
- **RETINOL AND NIACIN (35):** retinol, niacin
- **FATS PATTERN (81):** fats
- **SEAFOOD (46): EPA, DHA**, selenium

VEGETABLE-ORIENTED

STARCHY PATTERNS

- **STARCH RICH (33-44, 73): starch, vegetable protein**, sodium
- **STARCH RICH (45): starch, total protein**, sodium, phosphorus, iron, zinc, magnesium, selenium, thiamin, niacin
- **TRADITIONAL (82): starch, total protein**, alcohol, nitrite, NDMA
- **REGIONAL (78): starch, vegetable protein**, other PUFAs
- **PLANT PROTEINS/POLYUNSATURATED FATS PATTERN (81): plant proteins, PUFAs**

ANIMAL-ORIENTED

ANIMAL-BASED PATTERNS

- **ANIMAL PRODUCTS (33): animal protein**, animal fat, **calcium, cholesterol, SFAs, riboflavin, phosphorus**, zinc
- **ANIMAL PRODUCTS (35): animal protein**, animal fat, **calcium, cholesterol, SFAs, riboflavin, phosphorus**
- **ANIMAL PRODUCTS (37-41, 43, 44, 73): animal protein, calcium, cholesterol, SFAs, riboflavin, phosphorus**, zinc
- **ANIMAL PRODUCTS (36, 42): animal protein, calcium, cholesterol, SFAs, riboflavin, phosphorus**
- **ANIMAL PRODUCTS (34): animal protein, calcium, cholesterol, riboflavin, phosphorus**, zinc
- **ANIMAL PRODUCTS (78): animal protein, calcium, cholesterol, SFAs, riboflavin, phosphorus**, zinc, niacin, vitamin D, sodium, vitamin B6, retinol, thiamin, LA
- **ANIMAL PRODUCTS (45): cholesterol, SFAs**
- **ANIMAL PROTEIN PATTERN (81): animal protein**
- **REFINED (82): total protein, SFAs, cholesterol**, other PUFA**,** sugar, retinol, vitamin E, vitamin D, NDMA
- **MEAT AND POTATOES (46): proteins, zinc, niacin, vitamin B6, thiamin**
- **DAIRY PRODUCTS (46): calcium, riboflavin, phosphorus**, biotin, magnesium, pantothenic acid, iodine

VEGETABLE-ORIENTED

VEGETABLE-SOURCE FATTY ACIDS

- **UNSATURATED FAT (33, 35):** MUFA, PUFA, vegetable fat, **vitamin E**
- **UNSATURATED FATS (39): LA, ALA**, other PUFA, **vitamin E**
- **VUFA (36-38, 40-43, 45): LA, ALA, vitamin E**
- **VUFA (34):** MUFA, **LA, ALA**, other PUFA, **vitamin E**
- **FATTY ACIDS WITH LEAVES (73):** MUFA, **LA, ALA, Vitamin E**, Lycopene
- **FAT RICH (82):** MUFA, oleic acid, **LA, ALA, vitamin E**, cholesterol, SFAs
- **VEGETAL OILS PATTERN (81):** vegetal oils
- **FATS (46):** MUFAs**, LA,** oleic acid**,** SFAs

FACTOR 1 (79): fat, protein, carbohydrate, energy, MUFA, SFA, vitamin E

[1]Dietary patterns that look similar (based on original loadings and text description) were placed one close to the other and consistently indicated with the same color code. When dietary patterns were virtually identical, we synthetized them as one cell. Dietary patterns left in white were too far from the others to be indicated with a color code. Variants of the same color indicate different subgroups of dietary patterns within the same group, with loadings showing modest but nutritionally relevant differences across color-specific subgroups

Results were separately displayed for food-based (left) and nutrient-based (right) patterns and for adults and children/adolescents (consistently indicated in violet). Food-based and nutrient-based patterns were juxtaposed based on correlation coefficients between nutrient-based dietary patterns and selected food groups, as provided in most of the original articles. Arrows linking the different groups indicate stronger (solid line) and weaker (dashed line) similarities between food-based and nutrient-based dietary patterns

ABBREVIATIONS: ALA, alpha-linolenic acid; AUFA, Animal Unsaturated Fatty Acids; DHA, docosahexaenoic acid; DP, dietary pattern; DPA, docosapentaenoic acid; EPA, eicosapentaenoic acid; FA, factor analysis (factor name from original articles); LA, linoleic acid; MUFA, monounsaturated fatty acid; NDMA, N-nitrosodimethylamine; PC, principal component (analysis) (principal component names from original articles); PUFA, polyunsaturated fatty acid(s); RAE, Retinol Activity Equivalent; SFA, saturated fatty acid(s); VUFA, Vegetable Unsaturated Fatty Acids

Supplemental Figure 6. Seventy-six dietary patterns derived from the qualitative and quantitative assessment of their reproducibility (14), as organized in groups based on original text descriptions and loadings, as well as congruence coefficients[1]

FOOD-BASED DIETARY PATTERNS

MEDITERRANEAN-STYLE

MIXED-SALAD

- **SALAD VEGETABLES (60, 61):** olive oil, raw and cooked leafy vegetables, mixed vegetables in salad, raw tomatoes, raw carrots, and other fruiting vegetables; **(SALAD) VEGETABLES (59):** oil, raw leafy vegetables, carrots, tomatoes, dressings
- **(SALAD) VEGETABLES (58):** oil, raw leafy vegetables, carrots, tomatoes, dressings
- **OLIVE OIL AND SALAD (55, 56):** olive oil, raw leafy vegetables, raw tomatoes, root vegetables, soup, chicken and turkey
- **PC1 (57):** vegetable oils, leafy vegetables, fruiting vegetables, cabbage, other vegetables, legumes, fish
- **OLIVE OIL AND VEGETABLES (47-54):** olive oil, raw and cooked vegetables, legumes, soups, fruits, fish, potatoes, bouillon, white meat, crustaceans and molluscs, crisp bread and rusks, nuts and dried fruits, yogurt, snacks, and fresh cheese

HEALTHY-PROTEIN FOODS AND SIDE DISH

- **PRUDENT (55, 56):** cooked leafy vegetables, legumes, cabbage, mushrooms, other vegetables, onions and garlic, fish, crustaceans and molluscs, seed oil, cooked tomatoes, fresh fruit (non-citrus), and nuts and seeds
- **HIGH FISH, FRUIT, NUTS (83):** fish, fruit, nuts
- **PRUDENT (66):** raw and cooked vegetables, legumes, soup, fish, fruit; **PRUDENT (64):** potatoes, cooked vegetables, legumes, soup, pizza; **PRUDENT (65):** potatoes, raw and cooked vegetables, legumes, soup, rice; **PRUDENT (68):** potatoes, raw and cooked vegetables, legumes, vegetable soups, olive oil; **PRUDENT (69):** potatoes, raw and cooked vegetables, legumes, soup, fish
- **LEGUMES, VEGETABLES AND FISH (67):** potatoes, raw and cooked vegetables, legumes, vegetable soup, fish
- **COOKED VEGETABLES (58):** potatoes, cabbages, carrots, cooked leafy vegetables, legumes, fish, rice, poultry
- **PRUDENT (60, 61):** high: potatoes, cooked leafy vegetables, cooked and raw carrots, other fruiting vegetables, pulses, fish, rice, poultry, yogurt, olive oil; low: wine and spirits
- **MEDITERRANEAN (PC2) (74):** positive: potatoes, raw and cooked vegetables, legumes, soups, olive oil, fish, fruits, pasta and other grains, bouillon, cooked tomatoes; negative: soft drinks
- **FACTOR 2/FA2/PC2 (62, 63):** potatoes, vegetables, fish, bread, cereals, oils; **WINTER PATTERN (75):** legumes, fish, seafood, whole grains, poultry
- **PATTERN 2 (80):** fish, vegetables, milk; **CEREALS/MEAT/FISH/OLIVE OIL PATTERN (81):** cereals, fish, olive oil, meat
- **PRUDENT (84):** stem-leafy-cooked-raw vegetables, cauliflower, legumes, blue fish, fresh caught-farmed fish, fruit, beef, yoghurt
- **WIDE RANGE (72):** vegetables, fish, fruits, cereals, meat, dairy products
- **VEGETARIAN (84):** high: potatoes, tubers, leafy-cooked-raw vegetables, cereals, lamb and mutton, fries, dried fruit, eggs, oil, butter; low: beef and fresh farmed fish
- **DP DIABETIC (76):** potatoes, vegetables, legumes, olive oil, fruits, nuts, dairy products, eggs, sweets and sugary foods
- **PRUDENT (70):** potatoes, cooked vegetables, legumes, fruits, nuts, yogurt, offals, shellfish, tea; **HEALTHY (71):** raw vegetables, cooked vegetables and beans, fresh or frozen fish (not fried), fresh fruits without added sugar, fresh meat (not fried)
- **HEALTHY (77):** cereals and tubers, vegetables, legumes, fish, fresh and dried fruit, dairy products, eggs, meat

TRADITIONAL PATTERNS

- **FARM-HOUSE DIET (75):** legumes, dairy products, vegetables, semolina type bread, fruits; **ELDERLY PATTERN (75):** legumes, whole milk, vegetables, semolina type bread
- **PATTERN 5 (80):** legumes, eggs, wine; **LEGUMES PATTERN (81):** legumes

WESTERN-STYLE

PASTA-AND-MEAT-ORIENTED

- **PATTERN 1 (80):** grains, meat, olive oil, potatoes
- **HIGH MEAT, ANIMAL FATS, GRAIN (83):** grains, meat, animal fats
- **PC2 (57):** pasta and rice, red meat, processed meat, other fats, sugar
- **CANTEEN (60, 61):** pasta, cooked tomatoes, veal, wine, olive oil, pulses, other fruiting vegetables, bread; **PASTA AND MEAT (47-54):** high: pasta and other grains, cooked tomatoes, red meat, white meat, offals, processed meat, animal fats, bread, wine, beer, olive oil, other sauces, seasoned cheese; low: breakfast cereals and yogurt; **PASTA AND MEAT (55, 56):** high: pasta, cooked tomatoes, beef, pork, processed meat, other animal fats, white bread, wine; low in yogurt
- **PORK, PROCESSED MEAT, POTATOES (58):** butter, non-fermented whole milk, cakes, eggs, pasta, beef, potatoes, processed meat
- **PORK, PROCESSED MEAT, POTATOES (59):** butter, pasta, potatoes, beef and veal, processed meat; **WESTERN (60, 61):** butter, potatoes, other pasta, processed meat, veal, eggs, cakes, beef, seed oils, offal, pork, cheese

CARBOHYDRATE (PC3) (74): positive: pasta and other grains, cooked tomatoes, animal fats, bread; negative: yogurt, fish, nuts and seeds, breakfast cereals, crustaceans and molluscs, tea, cooked vegetables, fruit juices, fruits, snacks, eggs

DAIRY PRODUCTS AND SWEETS

- **SWEET & DAIRY (55, 56):** cakes and puddings, sugar and honey and jam, chocolate-based confectionery, patisserie and biscuits, milk, cheese, eggs, butter, ice cream, coffee, processed meat; **FACTOR 1 (62):** pastries, sugar, milk, cheese, meat, fruit; **SWEETS (75):** desserts, chocolate, package products
- **RESIDUAL (PC4) (74):** positive: milk, coffee, white meats; negative: tea, wine, spirits
- **PATTERN 3 (80):** cheese, cakes, fruit; **PATTERN 6 (80):** cakes, biscuits, sugary drinks; **CAKES/FRUIT PATTERN (81):** cakes, fruit
- **EGGS AND SWEETS (47-54):** eggs, margarines, sugar and sweets, processed meat, vegetable oils, snacks, mayonnaises, butter, seasoned and fresh cheese, pizza, canned fish, fruit juices, coffee, soft drinks, potatoes, white meat, red meat, animal fats, bread, and beer; **HIGH EGGS AND SWEETS, LOW LEGUMES (83):** high: eggs and sweets; low: legumes; **FACTOR 3 (62):** eggs, alcoholic beverages
- **PATTERN 4 (80):** animal-based fats, cheese; **ANIMAL FATS/MARGARINES PATTERN (81):** animal fats/margarines
- **SPREADS (71):** butter and/or margarine on bread, reduced-fat products on bread, jam and honey, chocolate or nut-based spread

UNHEALTHY FOODS AND SNACKS

- **SNACK FOODS, PROCESSED MEATS AND OILS (67):** red meat, processed meat, chips, dipping sauces, snacks, vegetable oils, sugar and sweets, fruit juice
- **WESTERN (64, 65):** red meat, fries, dipping sauces, salty snacks, alcoholic drinks; **WESTERN (66):** fries, dipping sauces, salty snacks, vegetable oil, sweets, white bread; **WESTERN (68):** high: processed and red meats, chips, dipping sauces, snacks, plant oils; low: olive oil; **WESTERN (69):** high: processed meat, dipping sauces, salty snacks, vegetable oil, alcoholic drinks, canned fish; low fruits
- **WESTERN (PC1) (74):** positive: red meats, processed meats, mayonnaise and other sauces, salty biscuits, vegetable oils, sugar and sweets, soft drinks, spirits, beer and cider, wine, cheeses, eggs, butter, pizza, crustaceans and molluscs, offals; negative: toasted bread and rusks, fruits
- **HIGH ENERGY (84):** high: cold meats, fries, mayonnaise, salty snacks, bakery products, soft drinks, bread, butter, pasta, fresh and aged cheese, potatoes, pork; low: cereals
- **ENERGY-RICH (75):** cured meat, sausages, lean ham, bacon, packaged/fried foods, desserts, chocolate
- **DP NOT DIABETIC (76):** white, red and processed meat, sweets, sugary foods, caloric drinks, ready-to-eat dishes, wine, beer and spirits, seafood, grains
- **BEVERAGE AND CONVENIENCE (72):** high: beverages (alcoholic and nonalcoholic) and ready-to-eat dishes; low: plant foods and elaborate-to-cook dishes
- **WESTERN (70):** red and processed meat, dipping sauces, fries, vegetable oil, white bread, shellfish; **ENERGY DENSE (70):** dipping sauces, fries, sweets and refined sugar, pizza, yogurt, butter and margarine; **PROCESSED (71):** ketchup, mayonnaise and mayonnaise-based products, crisps, corn crisps and popcorn, chocolate and candy bars, sweetened drinks
- **UNHEALTHY (77):** meat, fast food, sweets, cereals and tubers, eggs, fish, dairy products

ALCOHOL (58): high: wine and spirits; low: coffee (with milk), non-fermented lowfat milk, cakes, other fruits

NUTRIENT-BASED DIETARY PATTERNS

VEGETABLE-ORIENTED

VEGETABLE-BASED PATTERNS

- **VITAMINS AND FIBER (33):** vitamin C, total fiber, β-carotene equivalents, total folate, potassium, soluble carbohydrates, vitamin B6; **VITAMINS AND FIBER (34, 39, 41, 43):** vitamin C, total fiber, β-carotene equivalents, total folate, potassium, soluble carbohydrates; **VITAMINS AND FIBER (37, 42):** vitamin C, total fiber, β-carotene equivalents, total folate; **VITAMINS AND FIBER (36, 38, 40):** vitamin C, total fiber, β-carotene equivalents, total folate, soluble carbohydrates; **VITAMINS AND FIBER (35):** vitamin C, total fiber, β-carotene equivalents, soluble carbohydrates; **VITAMINS AND FIBER (44):** vitamin C, total fiber, β-carotene equivalents, total folate, potassium, vitamin E
- **VITAMINS, MINERALS AND FIBERS (73):** vitamin C, fiber, β-carotene equivalents, folate, potassium, vitamin E, iron
- **VITAMINS AND FIBER (78):** vitamin C, total fiber, β-carotene, total folate, potassium, vitamin E, MUFA, soluble carbohydrates
- **ANTI-OXIDANT VITAMINS AND FIBER (45):** vitamin C, soluble and insoluble fiber, potassium, soluble carbohydrates, vitamin A (RAE), lignans, flavonoids
- **VITAMIN-RICH (82):** vitamin C, fiber, β-carotene, sugar, vitamin E, nitrates
- **FACTOR 3 (79):** vitamin C, soluble fiber, insoluble fiber, vitamin A, starch, iron
- **PLANT-BASED FOODS (46):** vitamin C, total fiber, β-carotene, folate, potassium, vitamin E, iron

ANIMAL-ORIENTED

ANIMAL-SOURCE FATTY ACIDS

- **AUFA (36, 37, 40, 42, 44):** other PUFA, vitamin D; **OTHER PUFA AND VITAMIN D (38, 41):** other PUFA, vitamin D, niacin
- **AUFA (45):** EPA, DHA, vitamin D
- **FATTY ACIDS WITH FINS (73):** EPA, DHA, DPA, vitamin D
- **FACTOR 2 (79):** PUFA, ω6, ω3
- **RETINOL AND NIACIN (35):** retinol, niacin
- **FATS PATTERN (81):** fats
- **SEAFOOD (46):** EPA, DHA, selenium

VEGETABLE-ORIENTED

STARCHY PATTERNS

- **STARCH RICH (33-44, 73):** starch, vegetable protein, sodium
- **STARCH RICH (45):** starch, total protein, sodium, phosphorus, iron, zinc, magnesium, selenium, thiamin, niacin
- **TRADITIONAL (82):** starch, total protein, alcohol, nitrite, NDMA
- **REGIONAL (78):** starch, vegetable protein, other PUFAs
- **PLANT PROTEINS/POLYUNSATURATED FATS PATTERN (81):** plant proteins, PUFAs

ANIMAL-ORIENTED

ANIMAL-BASED PATTERNS

- **ANIMAL PRODUCTS (33):** animal protein, animal fat, calcium, cholesterol, SFAs, riboflavin, phosphorus, zinc; **ANIMAL PRODUCTS (35):** animal protein, animal fat, calcium, cholesterol, SFAs, riboflavin, phosphorus; **ANIMAL PRODUCTS (37-41, 43, 44, 73):** animal protein, calcium, cholesterol, SFAs, riboflavin, phosphorus, zinc; **ANIMAL PRODUCTS (36, 42):** animal protein, calcium, cholesterol, SFAs, riboflavin, phosphorus; **ANIMAL PRODUCTS (34):** animal protein, calcium, cholesterol, riboflavin, phosphorus, zinc
- **ANIMAL PRODUCTS (78):** animal protein, calcium, cholesterol, SFAs, riboflavin, phosphorus, zinc, niacin, vitamin D, sodium, vitamin B6, retinol, thiamin, LA
- **ANIMAL PRODUCTS (45):** cholesterol, SFAs
- **ANIMAL PROTEIN PATTERN (81):** animal protein
- **REFINED (82):** total protein, SFAs, cholesterol, other PUFA, sugar, retinol, vitamin E, vitamin D, NDMA
- **MEAT AND POTATOES (46):** proteins, zinc, niacin, vitamin B6, thiamine; **DAIRY PRODUCTS (46):** calcium, riboflavin, phosphorus, biotin, magnesium, pantothenic acid, iodine

VEGETABLE-ORIENTED

VEGETABLE-SOURCE FATTY ACIDS

- **UNSATURATED FAT (33, 35):** MUFA, PUFA, vegetable fat, vitamin E; **UNSATURATED FATS (39):** LA, ALA, other PUFA, vitamin E; **VUFA (36-38, 40-43, 45):** LA, ALA, vitamin E; **VUFA (34):** MUFA, LA, ALA, other PUFA, vitamin E
- **FATTY ACIDS WITH LEAVES (73):** MUFA, LA, ALA, Vitamin E, Lycopene
- **FAT RICH (82):** MUFA, oleic acid, LA, ALA, vitamin E, cholesterol, SFAs
- **VEGETAL OILS PATTERN (81):** vegetal oils
- **FATS (46):** MUFAs, LA, oleic acid, SFAs

FACTOR 1 (79): fat, protein, carbohydrate, energy, MUFA, SFA, vitamin E

[1]Dietary patterns that look similar (based on original loadings and text description) were placed one close to the other and consistently indicated with the same color code. When dietary patterns were virtually identical, we synthetized them as one cell; their names were reported with the corresponding references, and separated by a ";" symbol. Variants of the same color across rows indicate different subgroups of dietary patterns within the same group, with loadings showing modest but nutritionally relevant differences across color-specific subgroups. Dietary patterns left in white were too far from the others to be indicated with a color code.
Results were separately displayed for food-based (left) and nutrient-based (right) patterns and for adults and children/adolescents (consistently indicated in violet). Food-based and nutrient-based patterns were juxtaposed based on correlation coefficients between nutrient-based dietary patterns and selected food groups, as provided in most of the original papers. Arrows linking the different groups indicate stronger (solid line) and weaker (dashed line) similarities between food-based and nutrient-based dietary patterns
ABBREVIATIONS: ALA, alpha-linolenic acid; AUFA, Animal Unsaturated Fatty Acids; DHA, docosahexaenoic acid; DP, dietary pattern; DPA, docosapentaenoic acid; EPA, eicosapentaenoic acid; FA, factor analysis (factor name from original papers); LA, linoleic acid; MUFA, monounsaturated fatty acid; NDMA, N-nitrosodimethylamine; PC, principal component (analysis) (principal component names from original papers); PUFA, polyunsaturated fatty acid(s); RAE, Retinol Activity Equivalent; SFA, saturated fatty acid(s); VUFA, Vegetable Unsaturated Fatty Acids
